# Hypothalamic-amygdala gating of caregiving-like prosocial behavior in mice

**DOI:** 10.1172/JCI204317

**Published:** 2026-10-01

**Authors:** Kai-Wen Geng, Rui-Rui Wang, Yan Yang, Yan Wang, Ting He, Chun-Li Li, Chong-Shun Xu, Jun Chen

**Affiliations:** Institute for Biomedical Sciences of Pain, Tangdu Hospital, The Fourth Military Medical University, Xi’an, Shaanxi, China.

**Keywords:** Cell biology, Neuroscience, Behavior

## Abstract

Distress sharing within an ingroup instinctively motivates caregiving-like prosocial behavior in social animals and humans, yet how aversive social stimuli paradoxically trigger such behavior remains unclear. Here, we show that stress-state matching in observer mice elicits caregiving-like behaviors (allogrooming and injury-targeted allolicking) toward cagemates in pain, resulting in synchronized social buffering. Importantly, we identify that the excitation of vasopressin receptor 1a–expressing (V1aR-expressing) neurons in the central medial amygdala (CeM) underlies both emotional-state matching and subsequent allogrooming/allolicking. This excitation is accomplished by arginine vasopressin (AVP) release from presynaptic terminals of the hypothalamic paraventricular nucleus (PVN) to act specifically on postsynaptic V1aR in the CeM. The CeM^V1aR^ neurons then project to the ventral tegmental area (VTA) to drive caregiving-like actions through positive reinforcement, manifested as prosocial preference and recurrent allogrooming/allolicking, and to produce anxiolysis in the caregiver. Collectively, we unravel a dedicated trisynaptic PVN^AVP^-CeM^V1aR^-VTA circuit driving and gating caregiving-like behaviors and advance the mechanistic understanding of AVP-V1aR signaling in prosociality.

## Introduction

Caregiving toward companions in distress or need, manifested as acts of assistance, comfort, and support, is a common feature of social animals (1–3). General caregiving, primarily involving affiliative social touch for emotional support, and targeted caregiving, directed at alleviating others’ local pain or injury, are essential for cohesion, survival, and well being of social species, including humans (1, 4–7). Conversely, deficits in caregiving behaviors are prominent hallmarks of several neuropsychiatric disorders, including autism spectrum disorders (ASDs), schizophrenia, and depression (8–10). Extensive empirical evidence has indicated that caregiving toward companions in pain or distress is attributed to empathic concern as a motivating factor (1, 11, 12) and can be seen and rapidly developed in infant chimpanzee and human children in their early years (13–16). The early onset, untrained, and autonomic nature of caregiving-like behavior across species suggests that it is presumably mediated by ancestral subcortical circuitry involving bottom-up emotional empathy (1, 17, 18). Meanwhile, caregiving-like behavior is highly adaptive in response to social experience, external contexts, and internal states (4-6, 19–22), implicating the engagement of cortical mechanisms related to top-down cognitive empathy (1, 4, 7, 17, 18).

Caregiving-like prosocial behavior encompasses an array of social components, including sharing of negative feelings, motivated approaching for physical investigation, comforting, and targeted helping (1–3). The complexity and richness of these partner-directed prosocial responses raise an open question regarding whether they share common or distinct circuit mechanisms in evolution. Recent insights into the neural mechanisms of prosocial behaviors toward distressed partners stem primarily from studies in laboratory rodents using different social stressors (4–6, 23–25). However, it still remains unknown how negatively valenced social stimuli are perceived and integrated into a prosocial desire to initiate and fulfill the caregiving-like behavior, even at the expense of self interests in response to others’ distress or need (26, 27). As altruistic helping and comforting often occur repeatedly and lead to personal relief or joy in the caregiver as well as the recipient, these behaviors may represent a positive adaptation to adverse situations (2, 28–30). Nevertheless, little is known about the neural mechanisms underlying this psychophysiological adaptation.

To address the above questions, we modified our previously established behavioral paradigm (6, 25) from intraplantar injection of melittin (31) to nape injection in order to produce ongoing inflammatory pain in the demonstrator. In this refined model, we observed that, although demonstrator mice experienced distress from being unable to groom or lick their nape, the melittin injection site nonetheless served as a salient cue for observer mice to direct social approach, allogrooming, and targeted allolicking. Leveraging this behavioral paradigm alongside optogenetics, fiber photometry, and patch-clamp recordings, we uncovered a molecularly specified, evolutionarily wired subcortical trisynaptic circuitry encoding emotional-state matching, motivational drive, and motor outputs, thereby driving and gating the caregiving-like behavior.

## Results

### Caregiving, driven by emotional-state matching, dynamically downregulates its driver.

As we proposed, subjecting a demonstrator mouse (e.g., care recipient) to nape melittin, a major pain-inducing peptide found in bee venom (31), resulted in rapid social investigation (approach and explore), followed by 15-min robust allogrooming and injury-targeted allolicking from the cagemate observer (e.g., caregiver) after reunion in comparison with naive and saline-treated groups (Figure 1, A–C, and Supplemental Video 1; supplemental material available online with this article; https://doi.org/10.1172/JCI204317DS1). Among caregiving-like behaviors directed toward the pain-suffering cagemate, 72% were allogrooming and 28% were allolicking (Figure 1D). In addition, observer mice spent significantly more time with pain-suffering cagemates than their naive counterparts in the 2-choice social affective preference (SAP) test, suggesting that mice are capable of not only recognizing a pain-affected emotional state but also demonstrating a prosocial preference toward distressed cagemates (Supplemental Figure 1). To confirm that caregiving-like behavior from the observer is prompted by pain-affected emotional state in the demonstrator, we coinjected bupivacaine (BPV) with melittin into the nape of the demonstrator to block peripheral pain signals and found that the observer performed less allogrooming/allolicking than the control (Figure 1E). To explore whether caregiving-like behaviors alleviate stress in the caregiver, we physically separated the observer from the pain sufferer using a transparent divider, which allowed visual/olfactory/auditory contact but prevented physical touch (Figure 1F). The results showed that the observers in the divider condition had significantly higher plasma corticosterone (CORT) levels than those in the undivided condition (Figure 1G), indicating that observers may reduce their shared stress through allogrooming/allolicking toward the pain sufferer. This interpretation was supported by a follow-up experiment, in which the amount of allogrooming and allolicking performed by caregivers in the divider-reunion group was comparable with that in the no-divider group (Figure 1, F and H), implying that social stress may accumulate in the observer until caregiving-like behaviors are engaged. To investigate whether stress-state matching in the observer drives allogrooming/allolicking, we pretreated observer mice with CP-154526 (32), a selective antagonist of the corticotropin-releasing factor receptor 1 (CRFR1), which is believed to initiate the physiological stress responses, including anxiety (33). Consistent with established evidence that anxiolytic treatment impaired helping behavior in rats (34), CP-154526 significantly decreased allogrooming/allolicking in mice relative to vehicle treatment (Figure 1I), demonstrating that empathic anxiety could be elicited by social stress exposure and drives subsequent caregiving-like actions.

Is the caregiving-like behavior sustainable or not? In our behavioral paradigm, allogrooming/allolicking peaked within the initial 5 minutes before progressively declining (Figure 1B), raising 3 possibilities from both sides of the social interaction: (a) emotionally reluctant to continue or (b) physical inability to continue due to exhaustion in caregivers, or (c) significantly diminished distress of the recipients. To explore the answer, we designed a new experiment in which each observer was successively exposed to 2 melittin-treated cagemates (mouse 1 and mouse 2) in consecutive 30-minute dyadic interaction sessions. The results showed no significant difference in allogrooming/allolicking duration toward 2 pain-suffering demonstrators, indicating that caregiving-like behavior functions as an active stress-coping strategy that correlates with demonstrators’ emotional states and may, in turn, alleviate their stress levels (Figure 1, J–L). To characterize the effect of different behaviors on stress regulation in both observers and demonstrators, we next systematically correlated the duration of various social/nonsocial behaviors (social investigation, allogrooming/allolicking, huddling and self grooming) with plasma CORT levels in both sides following 30-minute social interactions. In contrast with other behaviors showing no significant correlation with CORT levels in either observers or demonstrators (Figure 1, N–P, and Supplemental Figure 2, A–C), the amount of allogrooming/allolicking exhibited a strong negative correlation with CORT levels in both sides at the 30-minute endpoint (Figure 1, M and Q), suggesting that caregiving-like behavior per se plays a key role in achieving mutual social buffering. The strong positive correlation in the CORT levels between the recipients and the caregivers even after a 30 minute prosocial interaction highly supported synchronized stress buffering between the dyads (Figure 1R), indicating that emotional-state matching may both drive and be downregulated by the caregiving-like behavior.

### V1aR in the CeM is required for caregiving-like prosocial behavior.

Since AVP/OXT systems have been implicated in different prosocial behaviors (35-37), it is essential to know how they function in caregiving-like behaviors in mice. To determine this, we examined both vasopressin receptor 1a (*V1aR*) and *OXT*-null mutant male mice in a range of behavioral tests. Interestingly, *V1aR*-null mutants displayed impairments in both stress-state matching and allogrooming/allolicking, with the performances in general sociability, anxiety, and pain sensory tests being unchanged (Figure 2, A–G, and Supplemental Figure 3, A–L). To our surprise, congenital loss of V1aR resulted in social avoidance in the SAP test (Figure 2, H–J). These results suggest that V1aR signaling plays a crucial role in transforming distress sharing into caregiving-like prosocial responses. However, *OXT*-null mutants exhibited normal allogrooming/allolicking and prosocial preference (Supplemental Figure 3, M–T), indicating that functions of OXT in prosocial behaviors could differ across social context, familiarity, species, and sex (4, 23, 38–40).

To identify neural correlates of caregiving-like behavior, we performed c-Fos mapping of the caregiver and found robust c-Fos signals in multiple subcortical regions, including the central amygdala (CeA), ventral tegmental area (VTA), and hypothalamic paraventricular nucleus (PVN) (Figure 2, K–N, Supplemental Figure 4, and Supplemental Table 1). The most robust c-Fos change within the CeA, alongside the indispensable role of *V1aR* in caregiving-like behaviors, impelled us to examine *V1aR* expression in the CeA, a crucial hub for emotional processing (41). RNAscope in situ hybridization (ISH) showed that the majority of *V1aR* were concentrated in the CeM, with a sporadic few signals in the central lateral and capsular amygdala (CeL and CeC) (Supplemental Figure 5), consistent with the results of an early autoradiographic study (42). Next, we adopted siRNA-mediated *V1aR* knockdown in the CeM of observer mice to check its function in caregiving-like behavior (Figure 2, O and P). Notably, *V1aR* knockdown in the CeM impaired prosocial preference, stress-state matching, and allogrooming/allolicking, indicating a crucial role of CeM *V1aR* in mediating caregiving-like prosocial responses (Figure 2, Q–W). Additionally, we further examined the functional role of the *V1aR* in the PVN and VTA of observer mice during the prosocial interaction. In contrast, siRNA-mediated *V1aR* knockdown in either the PVN or the VTA does not affect allogrooming/allolicking (Supplemental Figure 6), suggesting that V1aR signaling in these nuclei is dispensable for the caregiving-like behavior.

### CeM^V1aR^ neuronal activation during caregiving-like behavior.

To associate CeM^V1aR^ neuronal activities with caregiving-like behaviors, we utilized *V1aR*^iCre^ knock-in mice and confirmed extensive colocalization between *iCre* and *V1aR* expressions in the CeM (Figure 3, A–D). We next performed in vivo fiber photometry recordings of CeM^V1aR^ neurons expressing AAV-hSyn-DIO-jGCaMP7s (*V1aR*^jGCaMP7s^ neurons) in *V1aR*^iCre^ mice, capturing both 470 nm (Ca²^+^-dependent) and 410 nm (isosbestic control) signals during social interactions (Figure 3, E and F). Confronting a demonstrator in pain triggered a rapid and sustained increase in Ca^2+^ transients of *V1aR*^jGCaMP7s^ neurons in the observer, lasting for dozens of seconds during social investigation (Figure 3G and Supplemental Video 2), followed by sharp increases, in alignment with the onset and cessation of allogrooming/allolicking episodes, respectively (Figure 3G, and Supplemental Videos 3 and 4). Interestingly, *V1aR*^jGCaMP7s^ neurons of both male and female observers showed a significantly stronger and more persistent pattern of activity in response to a cagemate in pain compared with naive controls in the initial social interaction, suggesting that these neurons encode the distressed state of pain sufferers rather than general social investigation (e.g., sniffing) (Figure 3, H–J, and Supplemental Figure 7, A–C). In addition, indistinguishable activation patterns of *V1aR*^jGCaMP7s^ neurons during allogrooming and allolicking indicated that both behaviors may share common motor components encoded by CeM^V1aR^ neurons (Figure 3, G and K–Q, and Supplemental Figure 7, D–J). By contrast, *V1aR*^jGCaMP7s^ neurons in both sexes became less active during self grooming (Figure 3, R–T, and Supplemental Figure 7, K–M). Together, these results demonstrate that CeM^V1aR^ neuronal activity are associated with caregiving-like behaviors in terms of emotional-state matching, prosocial approach, and engagement of allogrooming and allolicking.

### Gate control of caregiving-like behavior by CeM^V1aR^ neurons.

To determine the necessity of CeM^V1aR^ neurons in caregiving-like behavior, we ablated bilateral CeM^V1aR^ neurons by expressing TEVp-activated caspase-3 (taCasp3) in *V1aR*^iCre^ mice for behavioral tests (Figure 4, A and B). The taCasp3 observers exhibited prolonged latency and reduced time and bouts of allogrooming/allolicking toward the pain-suffering cagemates compared with eYFP controls, indicating an indispensable role of CeM^V1aR^ neurons in fulfilling caregiving-like behavior (Figure 4, C–F). Additionally, taCasp3 observers showed impaired prosocial preference to the pain sufferers in the SAP test while the performances in open field, elevated zero maze, spontaneous self-grooming, sociability and novelty tests remained unchanged (Figure 4, G–I, and Supplemental Figure 8, A–I). Moreover, chemogenetic inhibition of bilateral CeM^V1aR^ neurons expressing hM4Di in *V1aR*^iCre^ mice also resulted in impaired allogrooming/allolicking (Supplemental Figure 8, J–M), corroborating the findings in taCasp3 mice.

To confirm that CeM^V1aR^ neurons are capable of driving and controlling caregiving-like behavior, we next performed optogenetic activation of bilateral CeM^V1aR^ neurons expressing channelrhodopsin-2 (ChR2) in *V1aR*^iCre^ mice (Figure 4J and Supplemental Figure 9, A and B). Opto-activation of bilateral CeM^V1aR^ neurons in ChR2 observers of both sexes robustly elicited time-locked approaching and allogrooming toward both pain-suffering and naive cagemates, while injury-targeted allolicking was specifically elicited when ChR2 observers interacted with pain sufferers (Figure 4, K–M, Supplemental Figure 9, C–E, and Supplemental Video 5). In contrast, virtually no allogrooming or allolicking was elicited by laser stimulation in eYFP controls regardless of sex (Figure 4K and Supplemental Figure 9C). These results indicate that CeM^V1aR^ neurons sufficiently drive and maintain allogrooming/allolicking in both sexes. Intriguingly, opto-activation of CeM^V1aR^ neurons in ChR2 mice also evoked time-locked fictive licking, manifesting as rhythmic licking-like sequences in the absence of companions (Supplemental Figure 8N), suggesting that CeM^V1aR^ neurons directly control the licking action of caregiving-like behavior. Moreover, this opto-activation was behaviorally specific, as it failed to evoke aggression during social interaction or self grooming in a nonsocial setting (the same testing box without companions) (Supplemental Figure 8, O and P). Complementarily, opto-inhibition of bilateral CeM^V1aR^ neurons expressing Guillardia θ anion-conducting channelrhodopsin-2 (*Gt*ACR2) in *V1aR*^iCre^ observers promptly suppressed spontaneous allogrooming/allolicking toward pain sufferers; however, no such inhibitory effect occurred in eYFP controls (Figure 4, N–R, and Supplemental Video 6).

Since allogrooming has also been observed in bystander mice toward distressed conspecifics that experienced electric foot shocks (FS) (4, 5), we examined the role of CeM^V1aR^ neurons in the FS test as well. Ablation of CeM^V1aR^ neurons in taCasp3 observers also reduced allogrooming toward FS-treated cagemates, while no such effect was observed in eYFP controls (Supplemental Figure 10), implicating that CeM^V1aR^ neurons constitute the common neural substrate underlying prosocial behaviors in response to various social distresses. Collectively, these results suggest that CeM^V1aR^ neurons may serve as a hub integrating negative social stimuli for emotional-state matching, encoding motivational drive for social approach and preference, and governing subsequent allogrooming/allolicking.

### PVN^AVP^-CeM^V1aR^ circuitry initiates emotional-state matching and caregiving.

AVP has crucial roles in social behaviors via V1aR (35). However, it remains unknown whether AVP neurons form monosynaptic connections with CeM^V1aR^ neurons. To validate this, we conducted rabies virus–mediated monosynaptic retrograde tracing (Figure 5, A and B). We found that DsRed-labeled AVP neurons, but not OXT neurons, within the PVN (Figure 5, C and D), while the supraoptic nucleus (SON) showed few DsRed signals (Figure 5, E and F), demonstrating monosynaptic innervations of PVN^AVP^ neurons onto CeM^V1aR^ neurons (referred to as PVN^AVP^- CeM^V1aR^ circuitry). PVN is a key hub for integrating substantial physiological stimuli and mediating emotional arousal and adaptive behaviors (36). As such, it is tempting to speculate that the PVN is upstream of the CeM, receiving prosocial signals and encoding emotional-state matching for triggering subsequent caregiving. To confirm our hypothesis, we next validated *Avp*^Cre^ mice and ablated PVN^AVP^ neurons by expressing taCasp3 in *Avp*^Cre^ mice for behavioral tests (Figure 5, G and H, and Supplemental Figure 11, A–E). Consistently, taCasp3 mice displayed impairments in prosocial preference, stress-state matching, and allogrooming/allolicking compared with eYFP controls (Figure 5, I–P). We also conducted chemogenetic inhibition of bilateral PVN^AVP^ neurons expressing hM4Di in *Avp*^Cre^ mice for behavioral tests (Supplemental Figure 11F). Likewise, this neuronal inhibition led to impaired allogrooming/allolicking in terms of latency, time, and bouts relative to eYFP controls, thereby supporting the observations in taCasp3 mice (Supplemental Figure 11, G–I).

To corroborate the role of PVN^AVP^ neurons in triggering caregiving-like behavior, we optogenetically activated bilateral PVN^AVP^ somas or their axonal terminals in the CeM using ChR2 in *Avp*^Cre^ observers, respectively (Figure 5, Q and U). Opto-activation of either PVN^AVP^ somas or their CeM-projecting terminals evoked time-locked allogrooming/allolicking toward melittin-treated cagemates (Figure 5, R–T and V–X, and Supplemental Video 7); however, the evoked behavior was delayed and weaker compared with that elicited by opto-activation of CeM^V1aR^ somas (Supplemental Figure 12). This difference was particularly evident in the minimal fictive licking elicited by PVN^AVP^ (soma or terminal) stimulation (Supplemental Figure 11, J and M) versus the robust fictive licking evoked by CeM^V1aR^ soma stimulation (Supplemental Figure 8N). Furthermore, neither PVN^AVP^ soma nor terminal manipulation elicited attacking during social interaction or self grooming/self licking in the nonsocial context (Supplemental Figure 11, K, L, N, and O), demonstrating the behavioral specificity of the PVN^AVP^-CeM^V1aR^ circuitry. Together, these results suggest that PVN^AVP^-CeM^V1aR^ circuitry is sufficient and selective for initiating caregiving-like behavior.

### The specificity of CeM AVP-V1aR signaling for caregiving-like behavior.

Given the anatomical proximity of OXTR and V1aR in the CeA (42) and their bidirectional interactions with AVP or OXT (43), we further investigated the specificity of AVP-mediated activation of CeM^V1aR^ neurons in vitro by injecting AAV-DIO-mCherry in the CeM of *V1aR*^iCre^ mice and obtaining the brain slices containing mCherry-expressing CeM^V1aR^ (*V1aR*^mCherry^) neurons for whole-cell patch-clamp recording (Figure 6, A and B). Application of AVP using a low dose (0.2 μM) that could avoid nonspecific activation of OXTR (42) evoked a progressive membrane depolarization and persistent firing of *V1aR*^mCherry^ neurons (Figure 6, C, G, and H). 84.6% (22 out of 26) of recorded *V1aR*^mCherry^ neurons showed increased firing activity following AVP application, suggesting widespread excitability of CeM^V1aR^ neurons to exogenous AVP (Figure 6, C and H). By contrast, OXT application using a higher dose (1 μM) induced neither membrane depolarization nor firing increase of *V1aR*^mCherry^ neurons (Figure 6, D, G, and H), indicating that OXT has no detectable effect on V1aR. Moreover, both membrane depolarization and firing response of *V1aR*^mCherry^ neurons in response to AVP application were completely blocked by V1aR antagonist (Manning, 1 μM) (42), but not OXTR antagonist (L-368899, 1 μM) (44) (Figure 6, E–H). Collectively, these results strongly demonstrate the sufficiency and necessity of exogenous AVP in activating CeM^V1aR^ neurons without cross reactivity between OXT and V1aR in CeM^V1aR^ neurons.

The specific activation of CeM^V1aR^ neurons by AVP-V1aR signaling in patch-clamp recording prompted us to check whether this specificity operates in vivo. To confirm this, we equipped mice with bilateral cannula implantations for local drug delivery into the CeA (Figure 6, I–K). Consistently, allogrooming and allolicking were significantly suppressed by pumping manning, but not L-368899, into bilateral CeA (Figure 6, L–Q), suggesting a specific requirement of local AVP-V1aR signaling in mediating caregiving-like behavior.

### Postsynaptic activation of CeM^V1aR^ neurons by AVP release from the PVN.

We next investigated whether AVP release from the PVN into the CeM could evoke membrane depolarization and firing increase of CeM^V1aR^ neurons as exogenous AVP did by expressing AAV-mAVP-ChR2-eGFP under the control of a mouse AVP promoter (45) in the PVN and AAV-DIO-mCherry in the CeM of *V1aR*^iCre^ mice, respectively (Figure 7, A–C). Whole-cell patch-clamp recording of *V1aR*^mCherry^ neurons was made under burst opto-activation of PVN^AVP^ axonal terminals in the CeM (Figure 7, A–C). Consistent with our hypothesis, *V1aR*^mCherry^ neurons exhibited rapid membrane depolarization and firing increase in response to burst opto-activation (Figure 7, D–F), both of which were completely blocked by Manning (1 μM) (Figure 7, G–I). To test whether glutamate transmission is required for this postsynaptic activation of CeM^V1aR^ neurons, we blocked both AMPA and NMDA receptors by coapplication of CNQX (20 μM) (46) and AP5 (50 μM) (46) onto *V1aR*^mCherry^ neurons under opto-activation of PVN^AVP^-CeM^V1aR^ circuit. Interestingly, coapplication of CNQX and AP5 abolished neither membrane depolarization nor firing increase of *V1aR*^mCherry^ neurons in response to burst opto-activation (Figure 7, J–L). Taken together, these findings suggest that CeM^V1aR^ neurons could be directly and specifically activated by presynaptic AVP release from the PVN via postsynaptic V1aR.

### CeM^V1aR^-VTA projection promotes caregiving-like behavior by encoding its positive valence.

Owing to the CeM as an output station of the CeA (47), the downstream target of CeM^V1aR^ neurons underlying caregiving-like behavior needs to be determined. Given robust c-Fos activation in the VTA after prosocial interaction (Figure 2K) and the crucial role of VTA in integrating social motivations and reinforcing their respective behavioral outputs (48, 49), we proposed that the VTA should be a downstream candidate for motivating and reinforcing caregiving-like prosocial responses. To address our hypothesis, we first optogenetically silenced the axonal terminals of CeM^V1aR^ neurons in the VTA by expressing AAV-DIO-mCherry or AAV-DIO-eNpHR3.0-mCherry virus in the CeM of *V1aR*^iCre^ mice and implanted the optic fibers above the VTA (Figure 8, A and B). Opto-inhibition in eNpHR3.0 observers significantly prolonged the latency and reduced the time and bouts of allogrooming/allolicking compared to mCherry controls, suggesting requirement of the CeM^V1aR^-VTA projection in driving and reinforcing caregiving-like behavior (Figure 8, C–F). To ascertain whether VTA projections of CeM^V1aR^ neurons sufficiently promote caregiving-like behavior, we next optogenetically activated ChR2-expressing axonal terminals of CeM^V1aR^ neurons in the VTA (Figure 8G). Like soma stimulation, terminal activation robustly evoked time-locked allogrooming/allolicking toward both naive and pain-suffering cagemates without attacking, indicating that CeM^V1aR^ neurons convey motivational drive for caregiving via projections to the VTA (Figure 8, H–J, and Supplemental Figure 13C). Furthermore, in the nonsocial context, the same terminal activation evoked robust fictive licking but virtually no self grooming/self licking in ChR2 mice (Supplemental Figure 13, A and B), suggesting that CeM^V1aR^-VTA projection encodes the motor sequence for the licking action of caregiving-like behavior. To rule out antidromic or collateral activation, we inhibited the CeA by lidocaine (5) infusion before optogenetically activating VTA projecting CeM^V1aR^ terminals (Figure 8K). Pharmacological inhibition of CeM^V1aR^ somas did not prevent the evoked allogrooming/allolicking by their VTA terminal activation, whereas this inhibition by lidocaine abolished spontaneous allogrooming/allolicking (Figure 8, L–O). These results indicate that CeM^V1aR^ neurons promote caregiving-like behavior by relaying prosocial information from the PVN^AVP^-CeM^V1aR^ circuitry to the VTA, which encodes motivational drive and motor sequences for caregiving.

Why do caregiver mice repeatedly perform allogrooming and allolicking toward the pain-suffering cagemate? Although it is well documented that prosocial preference (preferentially approaching the pain sufferer in the SAP test) leads to affiliative touch that calms the recipient (4, 5, 28), the neural mechanisms driving this recurrent performance in the caregiver remain unclear. To investigate whether the CeM^V1aR^-VTA projection underlies this prosocial preference, we combined the optogenetics with the real-time place preference test. Opto-activation of the CeM^V1aR^-VTA projection in ChR2 mice induced a significant preference for the laser-paired chamber, whereas eYFP controls showed no preference, suggesting that this projection encodes positive valence (Figure 8, P–R). Given that the CeM^V1aR^-VTA projection drives caregiving, thereby reducing stress transmission in the caregiver, we hypothesize that engaging in caregiving inherently elicits positive affective states through stress relief. To test this, we exposed ChR2 or eYFP observers to naive or pain-suffering cagemates separated by a transparent divider for 25 minutes, followed by measuring plasma CORT levels of each observer with or without opto-activation (laser: 5 minutes off and 20 minutes on) of the CeM^V1aR^-VTA projection (Figure 8S). Strikingly, after exposure to pain-suffering cagemates, ChR2 observers with opto-activation displayed plasma CORT levels similar to baseline, which were significantly lower than those in eYFP controls under identical conditions. This indicates that activation of the CeM^V1aR^-VTA projection itself confers resilience to the stress transmission from the pain sufferer on the caregiver (Figure 8, S and T). Together, these results provide further evidence that the CeM^V1aR^-VTA circuitry endows caregiving-like behavior with positive valence, explaining why caregiver mice proactively and repeatedly engage in this behavior toward distressed conspecifics.

## Discussion

As caregiving toward others in distress is crucial for group survival and continuity in both social animals and humans, elucidating its molecular and neural basis holds great biological significance for understanding the evolution of reciprocal altruism and social cooperation and may provide valuable insights for treating mental disorders in humans (1, 2, 50). Here, using multidisciplinary approaches, our study reveals key neural principles underlying this behavior.

To induce caregiving-like prosocial behaviors, a caregiver must perceive and share the altered affective state of a distressed conspecific, a process known as affective empathy (1, 2, 17, 18, 51). Although cortical mechanisms for emotional perception and contagion have been investigated (52–56), subcortical neural substrates encoding the shared state that motivates caregiving-like behavior remain elusive. In the current study, we present the first evidence that this resonating stress response is initiated by PVN^AVP^ neurons and then transmitted to CeM^V1aR^ neurons via synaptic AVP-V1aR signaling to elicit emotional-state matching for motivating prosocial actions. Consistently, fiber photometry results indicate that CeM^V1aR^ neurons responded robustly to the cagemate experiencing pain during initial social investigation. This response may represent the formation of emotional-state matching, which occurs prior to the sharp increases in neural activity denoting caregiving-like actions (Figure 3G). Given the anxiogenic role of AVP system (36) and the involvement of PVN-CeA connections in one’s own anxiety and stress regulation (57, 58), it is conceivable from an evolutionary standpoint that empathic anxiety may recruit the same neural mechanisms identified in this study. A recent study strongly argued that OXTR signaling is not genetically essential for either pair bonding or parenting in both genders of the prairie vole, indicative of other mechanisms substituting for the loss of OXTR functions in prosocial behaviors (59). Our work complements this pending issue by identifying V1aR and its expressing neurons in the CeM as indispensable substrates for converting distress sharing to caregiving-like prosocial behavior in mice. Although OXT could also be released from the PVN into the CeA to process emotional information (40, 58, 60), the lack of its involvement in caregiving-like behavior toward cagemates in pain suggests that separate, parallel neurocircuits from the PVN to the CeA utilizing either AVP or OXT may encode different emotional valences depending on the social context.

The neural substrates generating caregiving toward companions in distress or pain remain largely unknown. Utilizing *AVP*^Cre^ and *V1aR*^iCre^ mice, we have obtained long-sought access to central AVP-V1aR neural circuitry. We established the causal role of the PVN^AVP^-CeM^V1aR^ circuitry in initiating caregiving-like behavior while the CeM^V1aR^ soma and its VTA projection in governing motivational drive and motor sequences of this behavior. Besides, we also demonstrated the specificity of the PVN^AVP^-CeM^V1aR^-VTA circuitry for this behavior by following key findings: (a) the duration of evoked caregiving-like behavior in ChR2 observers of this circuitry is longer when demonstrators are in pain than naive (Figures 4L, 5S, 5W, and 8I), consistent with increased spontaneous caregiving-like behavior toward pain-suffering cagemate (Figure 1C); (b) any manipulation in this circuitry affects neither aggression nor spontaneous self grooming. A prior study identified distinct neural mechanisms in the ACC that regulate allogrooming and allolicking (7). However, our results indicate that, at both the motivational and executive levels, the 2 behaviors may share common subcortical mechanisms mediated by CeM^V1aR^ neuronal circuitries. This discrepancy suggests that cortical and subcortical mechanisms are not redundant but rather operate at different levels of behavioral control: the CeM serves as a permissive gate for motivational drive and behavioral execution, while the ACC contributes to the selection of behavior-specific patterns. The CeA is one of the most evolutionarily conserved amygdala subnuclei (61) and is well known for processing defensive and appetitive responses (41, 42, 62–64); however, our findings unravel a previously unappreciated coding capacity of the CeA, showing that CeM^V1aR^ neurons encode emotional-state matching, motivational drive, and motor sequences of caregiving-like behavior. Unlike the MeA primarily receiving olfactory inputs for inducing general allogrooming toward distressed conspecifics (5, 65), the CeA is well positioned as a converging node for integrating salient signals from multisensory pathways (62, 66, 67), which may explain the remarkable precision in allolicking toward the injury site involving visual inputs to achieve maximal benefit (25). Taken together, we provide direct evidence supporting the notion that the CeA, a striatum-like structure, serves as a command center executing motivational integration and behavioral control (41, 62, 63, 68, 69), while the PVN is hierarchically positioned above the CeA for processing salient stimuli, stress coping, and initiating subsequent behavioral responses (36, 38, 70, 71).

What underlies the psychophysiological significance of caregiving-like behavior? Gentle social touch during allogrooming and allolicking is anxiolytic and rewarding in the recipient through alleviating both psychological and physical distress (4, 5, 7, 28, 72). This, in turn, results in reduced stress transfer back to the caregiver (1, 2). However, it is not yet well understood why the caregiver repeatedly engages in caregiving, manifested as prosocial preference and recurrent allogrooming/allolicking, and how caregiving inherently affects the caregiver. A growing body of evidence suggests that prosocial behaviors per se could generate intrinsic emotional rewards in the caregiver and helper (29, 30). Consistently, utilizing optogenetics in different behavioral tests, our study uncovered diverse functional roles of the CeM^V1aR^-VTA circuitry in promoting caregiving-like behavior, encoding positive valence, and producing anxiolysis in the caregiver. Alternatively, this compelling evidence indicates that caregiving-like behavior may signal positive adaptation and confer resilience to social distresses by the CeM^V1aR^-VTA outputs, thereby rapidly leading to emotional relaxation in the caregiver (73). The VTA is a heterogeneous brain region that not only encodes reward and reinforcement but also regulates susceptibility and resilience to chronic social stress via discrete neural mechanisms (74–77). Given its multifunctional properties, the CeM^V1aR^-VTA circuitry may comprise segregated projections that relay distinct behavioral dimensions to different subtypes of VTA neurons. The detailed neural mechanisms regulating the prosocial information from the CeM to the VTA to interact with reward system require further investigations. Although we still don’t know how the CeM^V1aR^-VTA outputs modulate the hypothalamic-pituitary-adrenal (HPA) axis, direct modulation of the HPA axis by the VTA have already been reported (78, 79).

Preceding studies of AVP signaling in social behaviors have primarily employed pharmacological manipulations and genetic knockouts, which are limited by lack of receptor specificity, cross reactivity, or potential genetic compensation (43, 80–82). Furthermore, the diverse release paths and enriched receptors with sparse innervations have further obscured the precise role of AVP signaling in specific social contexts, including prosociality (43, 83). In this study, we demonstrate the remarkable specificity of PVN^AVP^-CeM^V1aR^ circuitry in caregiving-like behavior, including (a) non-overlapping expression of AVP and OXT in the PVN; (b) PVN^AVP^ but not PVN^OXT^ innervations onto CeM^V1aR^ neurons; (c) lack of cross reactivity between OXT and V1aR in the CeM; (d) postsynaptic activation of CeM^V1aR^ neurons by AVP^PVN^ independent of glutamate transmission; (e) evoked allogrooming/allolicking but neither self grooming/self licking nor aggression by opto-activations in this circuitry; (f) normal prosocial responses of *OXT-*null mutant mice; (g) impaired caregiving-like behavior by Manning but not L-368899 infusion into the CeA. These results suggest a molecular-defined neural circuitry from PVN to CeM, employing AVP as a neurotransmitter acting specifically at V1aR, specializes for encoding empathic sharing and initiating caregiving-like prosocial behavior.

In conclusion, we have delineated a specific trisynaptic PVN^AVP^-CeM^V1aR^-VTA circuitry that enables caregiver mice to share and match the stress state of suffering cagemates while driving and gating subsequent caregiving-like behaviors. This circuitry offers a candidate mechanism for prosocial deficits in humans. First, it resonates with human neuroimaging studies of extraordinary altruists, which show an enlarged, hyper-responsive amygdala to distress cues (84) and a correlation between VTA activity and empathic concern (27). Second, deficits in prosocial motivation are core features of several neuropsychiatric conditions (ASD, schizophrenia, and major depression) (8–10), raising the possibility that dysfunction within this circuitry may underlie these deficits. Notably, V1aR is a recognized drug target for sociability deficits (85). Our findings suggest that CeM-targeted V1aR modulation could be explored to enhance prosocial motivation. Given the evolutionary conservation of AVP system and amygdala across mammals (35, 43, 64, 86), it warrants investigation whether impairment of the PVN^AVP^-CeM^V1aR^-VTA circuitry underlies reduced empathic responsiveness in humans. Although direct translation from rodent allogrooming/allolicking to human prosocial behaviors requires caution, the conserved circuitry we have identified provides a mechanistic entry point for future translational studies.

## Methods

### Sex as a biological variable

Both sexes were included. The sex of the animals used was specified in each main figure legend. Specifically, all main figures represent data from males only, except for the patch-clamp experiment, which used male and female data equally. In addition, male and female mice were used in fiber photometry recording and optogenetic activation of CeM^V1aR^ neurons.

### Mice

C57BL/6J mice (10 to 16 weeks old) for experiments were purchased from the Beijing Vital River Laboratory Animal Technology Co., Ltd. *Avp*^Cre^ (023530, The Jackson Laboratory), *Oxt*^Cre^ (024234, The Jackson Laboratory), *V1aR* null mutant (005776, The Jackson Laboratory) and their respective wild-type littermates with age ranging from 12 to 16 weeks old were also used for experiments. 4 to 5 mice were co-housed per cage in standard conditions under 12/12-h light/dark cycle with food and water available *ad libitum*. All experiments were approved by the Animal Studies Committee at Fourth Military Medical University (FMMU IACUC 250138), and were performed in accordance with the guidelines of the National Institutes of Health and the International Association for the Study of Pain.

### Generation of V1aRiCre knock-in mice

The mouse *V1aR* gene is located on chromosome 10 with 2 exons. To generate the *V1aR*^iCre^ knock-in allele, an IRES-iCre cassette was integrated into the 3’UTR immediately downstream of the stop codon (TGA) in exon 2 using CRISPR/Cas9 technology which could minimize the possibility of disruption of *V1aR* expression and achieve iCre expression under the control of the regulatory elements of *V1aR* gene. The positive F1 progeny was validated by PCR, Southern blot and sequencing analysis, and subsequently bred to generate heterozygous *V1aR*^iCre^ for experiments.

### Behavior

#### Caregiving-like behavior elicited by nape injection of melittin.

4 to 5 mice per cage were cohoused for over 3 weeks to establish the familiarity and moved to the testing room the day before the test for overnight habituation. On the test day, each randomly designated observer was acclimated in a plastic testing box (20 cm × 20 cm × 15 cm) for 30 to 60 min. Each cagemate demonstrator was then either directly placed in the testing box without pain (naive) or immediately after receiving a subcutaneous injection of saline or melittin in the right nape which is well characterized as a pain-producing substance (31). The entire 30-min or 60-min dyadic social interaction was recorded by a digital camera (Canon). Videos from all experiments were viewed and quantified by experimenters blinded to the treatments and mouse genotypes. Social investigation is defined as partner-directed approaching and subsequent anogenital-, head- or body-oriented sniffing within a half head distance. Allogrooming is defined as head contact with the body or head of distressed demonstrators, accompanied by rhythmic head movements (4). Despite mainly focusing on the body or head of the recipient, allogrooming constantly changes the target area. Allolicking is defined as site-specific licking behavior toward the local pain or injury of the demonstrator (6, 25). It focuses on the specific site with pain or injury and normally remains there for a while. Allogrooming/allolicking latency is defined as the time from the placement of a demonstrator in the testing box to the onset of the first either allogrooming or allolicking from the observer. Allogrooming/allolicking time is defined as the actual time a mouse grooms and licks the other. Interval over 1 s is counted as separate bouts. Huddling is defined as low-motion close social contact between the observer and demonstrator (87). Self-grooming is defined as a repetitive stereotypic behavior comprising a sequence of stroking movements directed toward its own body with the front paws (88). All tests were performed between 8:00-12:00 am.

#### Divider paradigm.

For this behavioral test, transparent plastic testing boxes (20 cm × 20 cm × 15 cm) were used, with grid dividers placed in the middle to equally separate the testing space. The dividers had equally spaced gaps of 0.8 cm (width) × 9.0 cm (depth). On the test day, each observer was acclimated in the right half of the box with a divider for 40 to 60 min. Each cagemate demonstrator with or without nape melittin injection was then placed in the left half of the testing box. Each observer was anesthetized and immediately taken out for blood extraction after 25-30 min social interaction. In divider-reunion experiment, each observer underwent 25-30 min social interaction with its pain-suffering cagemate in the divider paradigm, followed by a 30-min reunion period under video recording.

#### Comforting-like behavior elicited by foot shock.

The behavioral assay was performed as previously described (5). Pairs of cagemates or littermates (co-housed over 3 weeks) with similar age and body weight were put into testing room for overnight habituation. On the test day, each randomly appointed demonstrator was removed from its home cage, transferred to a foot-shock chamber in a separate room, and subsequently given 20 foot-shocks (0.7 mA, 1 s) with a random interval between 20 s and 40 s. After returning demonstrators with foot shocks to their home cages, the 30-min dyadic prosocial interactions between observers and demonstrators were recorded by a digital camera (Canon) for further behavioral analysis. Latency, time and bouts of allogrooming were counted by experimenters blinded to the treatments and mouse genotypes. All tests were performed between 8:00-12:00 am.

#### Social affective preference (SAP) test.

Each observer was acclimated in a 3-chamber apparatus (63 cm × 25 cm × 30 cm) for 10 min before formal test. After confining a naive and a melittin-treated cagemate demonstrators into separate wire cylinders (20 cm high × 8 cm diameter; vertical bars, 0.5 cm apart) in each ending chamber (25 cm × 25 cm × 30 cm), each observer mouse was replaced into the middle (neutral) chamber (13 cm × 25 cm × 30 cm) and subsequent 30-min triadic social interaction was digitally recorded and analyzed by ANYMAZE 10 software (Stoelting). The wire cylinder allows sniffing and nose contact. Exploration time indicates the duration of observer mice spent in each chamber. Preference index was calculated as the percentage of time exploring the chamber with melittin-treated demonstrators over naive ones in the combined time spent in both chambers. All tests are performed between 8:00-12:00 am.

#### Sociability test and social novelty test.

Each observer was acclimated in a 3-chamber apparatus (63 cm × 25 cm × 30 cm) for 10 min before formal test. In sociability test, each observer was allowed to explore the apparatus with an empty wire cylinder and a wire cylinder confining a stranger mouse in each ending chamber for 10 min. In the subsequent social novelty test, we put the second stranger mouse in the empty wire cylinder and each observer was allowed to explore the apparatus for another 10 min. The preference index for sociability test was calculated as the percentage of time approaching and exploring the chamber with the stranger mouse over the empty chamber. The preference index for social novelty test was calculated as the percentage of time approaching and exploring the chamber with the second stranger mouse over the first stranger mouse.

#### Open field test.

Briefly, mice were individually placed in plastic open top arenas (40 x 40 x 30 cm) and then recorded for 10 min by an overhead camera connected to a dedicated computer equipped with ANYMAZE 10 software (Stoelting). ANYMAZE 10 software was used to analyze the time spent in the central area of the arena.

#### Elevated zero maze test.

The circular track of the maze is 5.5 cm wide, 35 cm in inner diameter, 46 cm in outer diameter and elevated 52 cm from the floor. The maze was divided into four quadrants of equal lengths with two opposing open quadrants and two opposing closed quadrants. 1 cm high curbs along open quadrants were to prevent falls and 15 cm high walls along the closed quadrants were to block the vision. Each 10-min trial began with the animal placed in the center of a closed quadrant and was recorded by an overhead camera (Canon). Offline analysis was completed later using ANYMAZE 10 software (Stoelting). The time spent in the open arms was measured and analyzed.

#### Acute pain measurements.

Mice were moved to the testing room and habituated in nontransparent plastic boxes (11 cm x 11cm x 18 cm) on the wire mesh stand for 1 h on the day before the formal tests. Mechanical pain sensitivity was measured using a set of calibrated von Frey filaments (North Coast, USA). Each filament was applied five consecutive times with 10 s intervals, and the minimum stimulus intensity that evoked three reflexive flinches out of five trials on the hind paw was taken as paw withdrawal threshold. Thermal pain sensitivity was assessed by a Hargreaves apparatus. The hind paw withdrawal latency was measured three times with 10-min intervals and averaged for analysis.

#### Spontaneous self-grooming.

Briefly, mice were individually placed in plastic boxes (11 cm x 11cm x 18 cm) and then digitally recorded for 30 min. The time of spontaneous self-grooming of each mouse was measured and analyzed.

### Code availability

Toolbox *cfosns* can be found on this website: https://psych.cas.cn/sourcedb/cn/expert/201602/t20160224_6369862.html

### Statistics

Statistical analyses were performed using Prism 8 (GraphPad). Data are presented as mean ± SEM or box and whiskers plots (center line: median; box limits: upper and lower quartiles; whiskers: 1.5 × interquartile range; points: outliers). Before statistical comparisons, normality test (Shapiro-Wilk test) was performed to all datasets. For non-Gaussian distribution data, 2-tailed Mann-Whitney test or Wilcoxon matched-pairs test was used to compare 2 groups; Kruskal-Wallis test with Dunn’s multiple comparisons was used to compare multiple groups. For Gaussian distribution data, 2-tailed unpaired/paired *t* test (Welch correction was applied, if variances are not equal between 2 groups) was used depending on the results of F test, which determines the similarity in the variances between 2 groups. Brown-Forsythe and Welch ANOVA tests with Dunnett T3 test, or parametric 1- or 2-way ANOVA with Tukey test multiple comparisons was used depending on the results of Bartlett’s test, which determines the equal variances among multiple groups. Significance levels indicated are as follows: *P* < 0.05 was considered statistically significant. Detailed information on statistical analyses is provided in Supplemental Tables 2 and 3.

Additional methodological details, including molecular biology experiments, chemogenetic and optogenetic experiments, fiber photometry recordings, and whole-cell patch-clamp recordings, are described in the Supplemental Methods.

### Data availability

All data required to evaluate the conclusions of this study are presented in the Supporting Data Values file.

## Author contributions

KWG participated in experimental design, performed most tests and analyzed the data. RRW and KWG participated in patch-clamp experiments. KWG and YY participated in IHC and RNAscope staining. KWG and YW participated in social affective preference tests. CSX, YY, TH, and CLL participated in mice breeding and genotyping. KWG and JC wrote the manuscript, conceived of, and supervised the project.

## Conflict of interest

The authors have declared that no conflict of interest exists.

## Funding support

- National Natural Science Foundation of China: 32100834 (KWG), 81571072 (JC), 82001180 (YY).
- Young Science and Technology Rising Star Project of Shaanxi Province: 2024ZC-KJXX-112 (KWG).
- Shaanxi Provincial Natural Science Foundation: 2025JC-YBQN-323 (KWG).
- University Hospital grant: 2019HJHZ001 (JC), 2023ATDQN015 (KWG), 2025JCRH044 (KWG).

## Supplementary Material

Supplemental data

Unedited blot and gel images

Supplemental video 1

Supplemental video 2

Supplemental video 3

Supplemental video 4

Supplemental video 5

Supplemental video 6

Supplemental video 7

Supporting data values

## Figures and Tables

**Figure 1:**
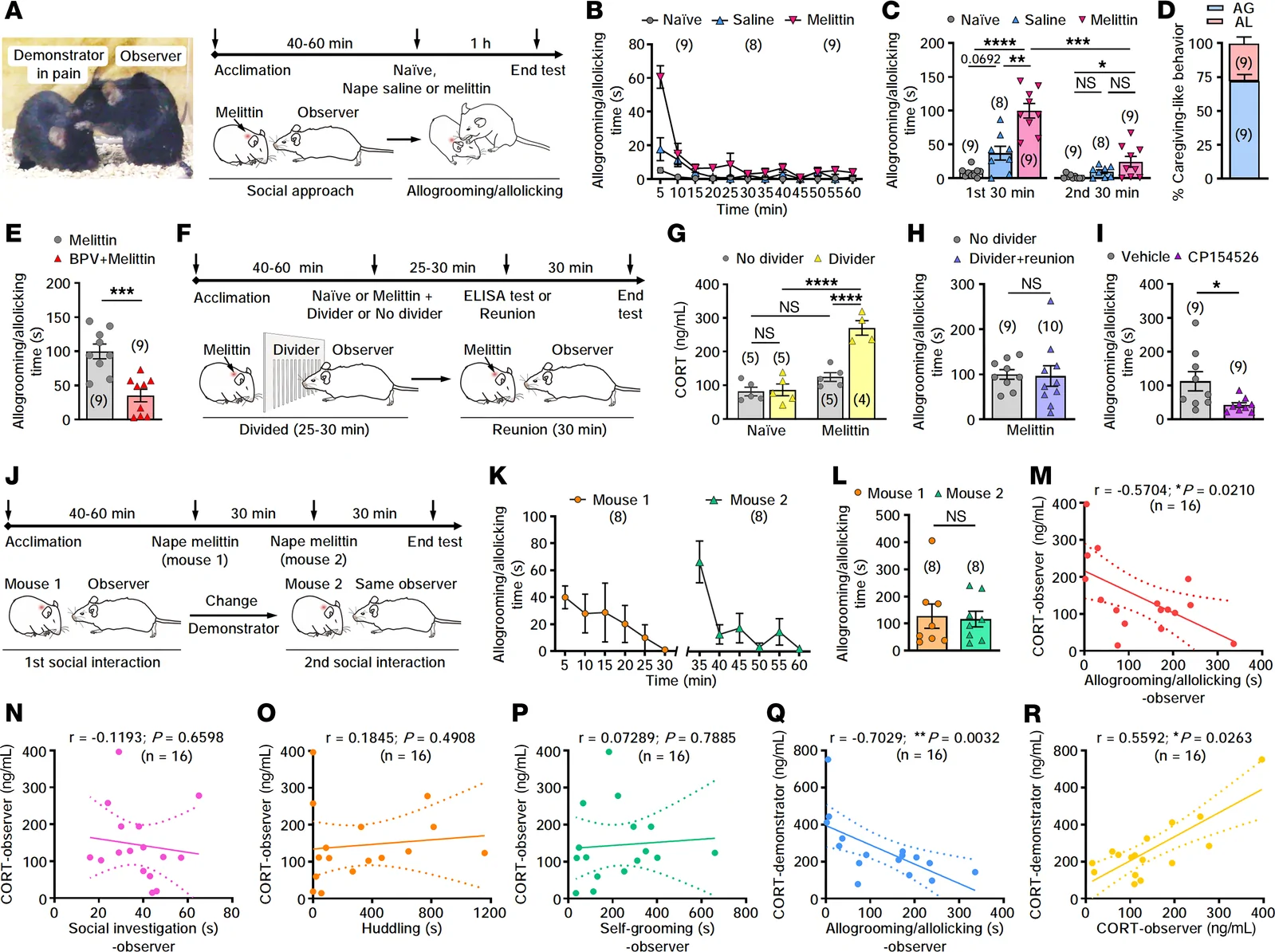
Stress coping and buffering nature of caregiving-like behavior in mice. (**A**) Left: a representative image of a mouse licking the injured right nape of its cagemate. Right: schematic of caregiving-like behavior test. (**B** and **C**) Time courses (**B**) and total durations (**C**) of allogrooming/allolicking toward naive, saline- and melittin-treated cagemates. (**D**) Proportions of allogrooming (AG) versus allolicking (AL) in total caregiving-like behavior toward pain-suffering cagemates. (**E**) Allogrooming/allolicking time toward melittin-treated demonstrators with or without bupivacaine (BPV) coinjection. (**F**) Schematic of divider paradigm followed by ELISA or caregiving-like behavior test. (**G**) Plasma CORT levels after 25–30 minutes social exposure to naive or melittin-treated demonstrator (with or without divider). (**H**) Observer allogrooming/allolicking duration toward melittin-treated cagemates: direct contact (no divider) versus reunion after 25–30 minutes divider separation (divider-reunion). (**I**) Allogrooming/allolicking time of observers pretreated with CRFR1 antagonist or saline toward pain-suffering cagemates. (**J**) Schematic of 2 consecutive caregiving-like behavior tests per observer. (**K** and **L**) Time courses (**K**) and total durations (**L**) of allogrooming/allolicking across 2 consecutive prosocial interactions. (**M**–**P**) Correlations between observers’ plasma CORT levels and duration of their allogrooming/allolicking (**M**), social investigation (**N**), huddling (**O**), and self-grooming (**P**) after 30-minute prosocial interactions. (**Q**) Correlation between demonstrators’ plasma CORT levels and received allogrooming/allolicking durations after 30-minute prosocial interactions. (**R**) Correlation between plasma CORT levels of observers and their demonstrators after 30-minute prosocial interaction. The control groups in **E** and **H** are the same dataset as the melittin group (first 30 minutes) in **C**. **M**–**R**, Solid lines, linear regression; dashed lines, 95% confidence intervals. **B**–**E**, **G**–**I**, **K**, **L**, mean ± SEM. All mice are male. Exact *n* values are indicated in the figure. AG, allogrooming; AL, allolicking; CRFR1, corticotropin-releasing factor receptor 1. 2. Brown-Forsythe and Welch ANOVA tests with Dunnett’s T3 multiple comparisons (1st 30 min) **C**; Kruskal-Wallis test with Dunn’s multiple comparisons (2nd 30 min) for **C**; Paired *t* test (Melittin, 1st vs 2nd) for **C**; Unpaired *t* test for **E**; 2-way ANOVA with Tukey’s multiple comparisons for **G**; Unpaired *t* test with Welch’s correction for **H** and **I**; Mann Whitney U test for **L**; Linear regression (Pearson correlation) for **M**, **N**, **P**; Linear regression (Spearman correlation) for **O**, **Q**, **R**.

**Figure 2:**
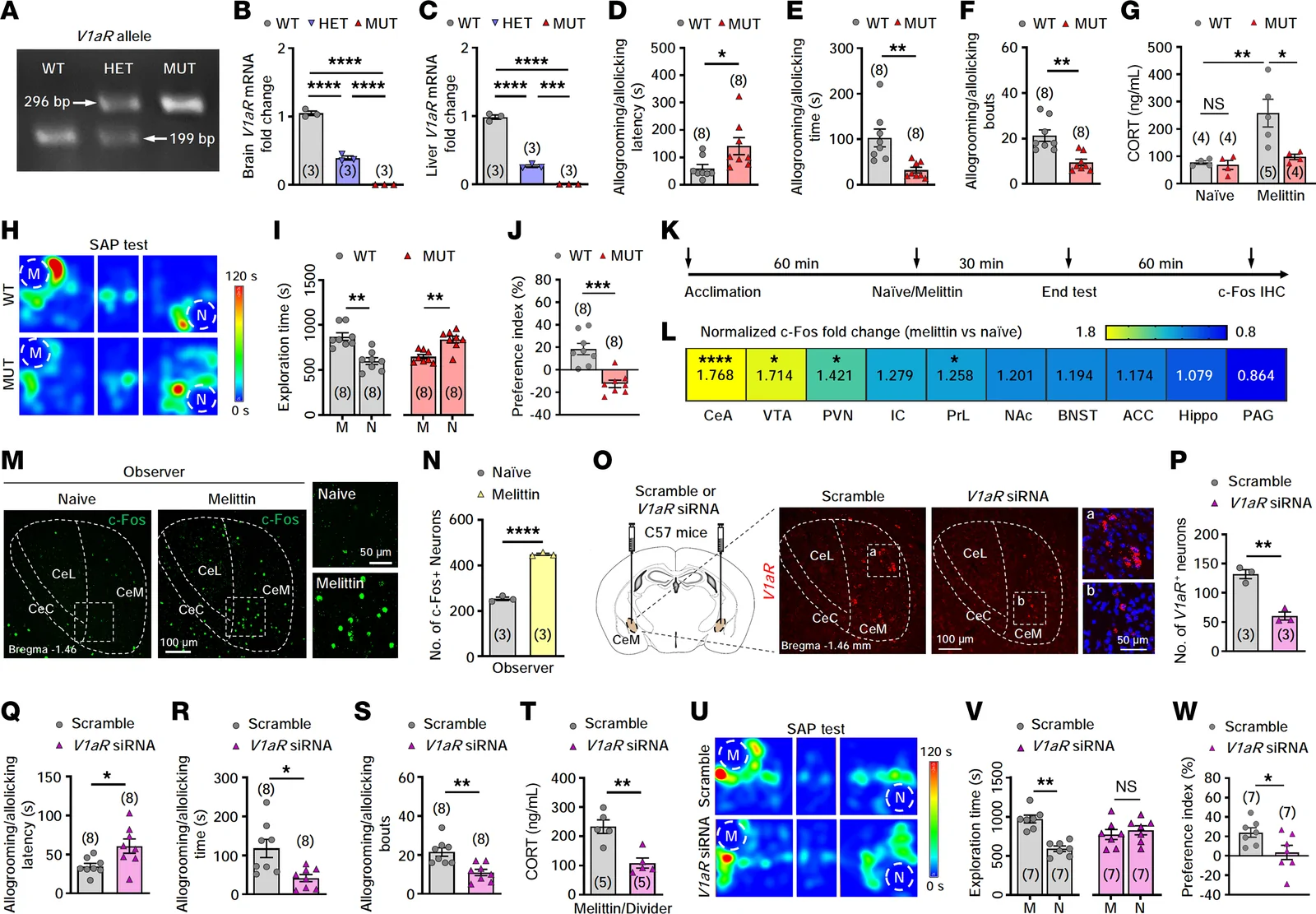
CeM V1aR is essential for caregiving-like behavior. (**A**) Genotyping of *V1aR*^wt/wt^ (WT), *V1aR*^null/wt^ (HET), and *V1aR*^null/null^ (MUT) mice. (**B** and **C**) Relative *V1aR* mRNA expression in the brain (**B**) and liver (**C**) of WT, HET, and MUT mice. (**D**–**F**) Latency (**D**), duration (**E**), and bouts (**F**) of allogrooming/allolicking toward pain-suffering cagemates in *V1aR* WT versus *V1aR* MUT observers. (**G**) Plasma CORT levels of *V1aR* WT and *V1aR* MUT observers after exposure to naive or melittin-treated demonstrator in divider paradigm. (**H**–**J**) Heatmaps of locomotor activity (**H**), exploration time (**I**), and preference index (**J**) of both *V1aR* WT and *V1aR* MUT mice in social affective preference (SAP) test. (**K** and **L**) Schematic of brain c-Fos mapping (**K**) and heatmap of normalized fold change in c-Fos^+^ cells across brain regions in observers exposed to melittin-treated versus naive cagemates (**L**). (**M** and **N**) Representative c-Fos images (**M**) and quantification (**N**) in the CeA of observers interacting with naive versus melittin-treated cagemates. (**O** and **P**) Schematic of CeM injection with scramble or *V1aR* siRNA (left), followed by measurements of *V1aR* mRNA expression (**O**) and statistics (**P**). (**Q**–**S**) Latency (**Q**), duration (**R**), and bouts (**S**) of allogrooming/allolicking in control and CeM *V1aR* knockdown observers toward pain-suffering cagemates. (**T**) Plasma CORT levels of control and CeM *V1aR* knockdown mice after 25–30 minutes social exposure to melittin-treated demonstrators in divider paradigm. (**U**–**W**) Heatmaps of locomotor activity (**U**), exploration time (**V**), and preference index (**W**) of control and CeM *V1aR* knockdown mice in SAP test. Scale bars: 100 μm (original), 50 μm (magnified) in **M** and **O**. Data are mean ± SEM. All mice are male. Exact *n* values are indicated in the figure. Statistical results are included in Supplemental Table 2. ACC, anterior cingulate cortex; BNST, bed nucleus of the stria terminalis; CeA, central amygdala; CeC, central capsular amygdala; CeL, central lateral amygdala; CeM, central medial amygdala; Hippo, hippocampus; IC, insular cortex; NAc, nucleus accumbens; PAG, periaqueductal gray; PrL, prelimbic cortex; PVN, hypothalamic paraventricular nucleus; VTA, ventral tegmental area. M, melittin; N, naive. SAP test, social affective preference test. One-way ANOVA with Tukey test multiple comparisons for **B** and **C**; Mann Whitney U test for **D** and **F**; 2-way ANOVA with Tukey’s multiple comparisons for **G**; Paired *t* test for **I** and **V**; Unpaired *t* test for **J**, **N**, **P**, **S**, **T** and **W**; Unpaired *t* test with Welch’s correction for **E**, **Q**, and **R**.

**Figure 3:**
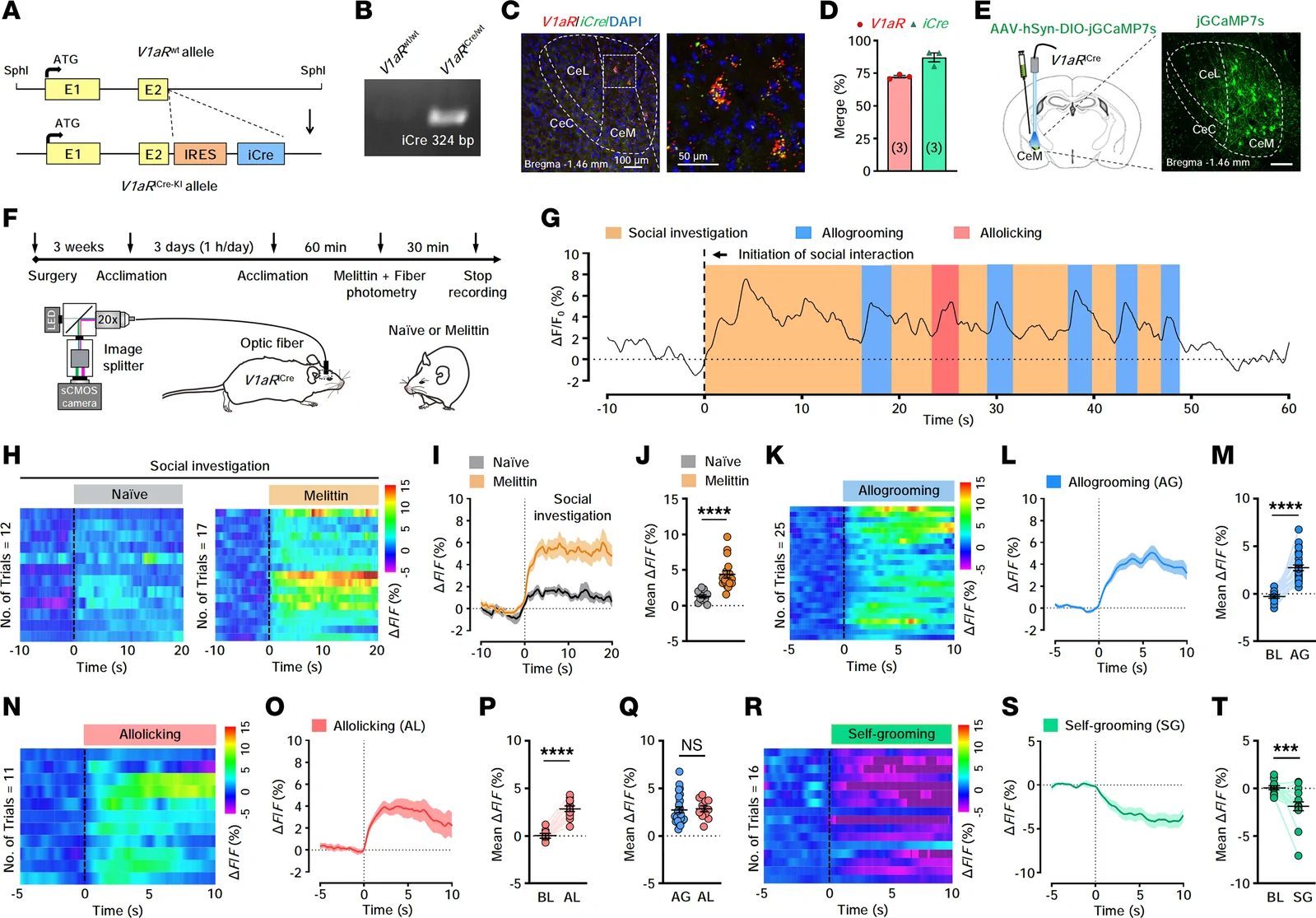
The calcium dynamics of CeM^V1aR^ neurons during different behavioral states. (**A**) Targeting strategy for generating *V1aR*^iCre^ knock-in mouse line. (**B**) Genotyping confirmation of wild-type and iCre positive mice. (**C** and **D**) Expression patterns (**C**) and coexpressing analysis (**D**) of *V1aR* mRNA and *iCre* mRNA in the CeM of *V1aR*^iCre^ mice. (**E**) Schematic of viral injection, fiber implantation, and jGCaMP7s expression in the CeM. (**F**) Schematic of fiber photometry recording during social interaction. (**G**) Continuous Ca^2+^ signals of *V1aR*^jGCaMP7s^ neurons during the initial social investigation (yellow shadowed), allogrooming (blue shadowed) and allolicking (pink shadowed). (**H**–**J**) Heatmaps (**H**), average traces (**I**), and mean Δ*F*/*F* (**J**) showing Ca^2+^ dynamics of *V1aR*^jGCaMP7s^ neurons in response to naive and melittin-treated cagemates. (**K**–**M** and **N**–**P**) Heatmaps (**K** and **N**), average traces (**L** and **O**), and mean Δ*F*/*F* (**M** and **P**) showing Ca^2+^ dynamics of *V1aR*^jGCaMP7s^ neurons before (baseline) and during allogrooming (**K**–**M**) or allolicking (**N**–**P**) episodes. (**Q**) Comparison of mean Δ*F*/*F* Ca^2+^ signals in *V1aR*^jGCaMP7s^ neurons during allogrooming and allolicking episodes. (**R**–**T**) Heatmap (**R**), average trace (**S**), and mean Δ*F*/*F* (**T**) showing Ca^2+^ dynamics of *V1aR*^jGCaMP7s^ neurons before (baseline) and during self-grooming episodes. Scale bars: 100 μm (original in **C** and **E**), 50 μm (magnified in **C**). Data are mean ± SEM. All mice are male. *n* = 3 mice in **D**; *n* = 12 or 17 trials from 7 mice in **H**–**J**; *n* = 25 trials from 5 mice in K–M; *n* = 11 trials from 5 mice in **N**–**P**; *n* = 16 trials from 5 mice in **R**–**T**. Statistical results are included in Supplemental Table 2. Mann Whitney U test for **J** and **Q**; Wilcoxon matched-pairs test for **M** and **T**; Paired *t* test for **P**.

**Figure 4:**
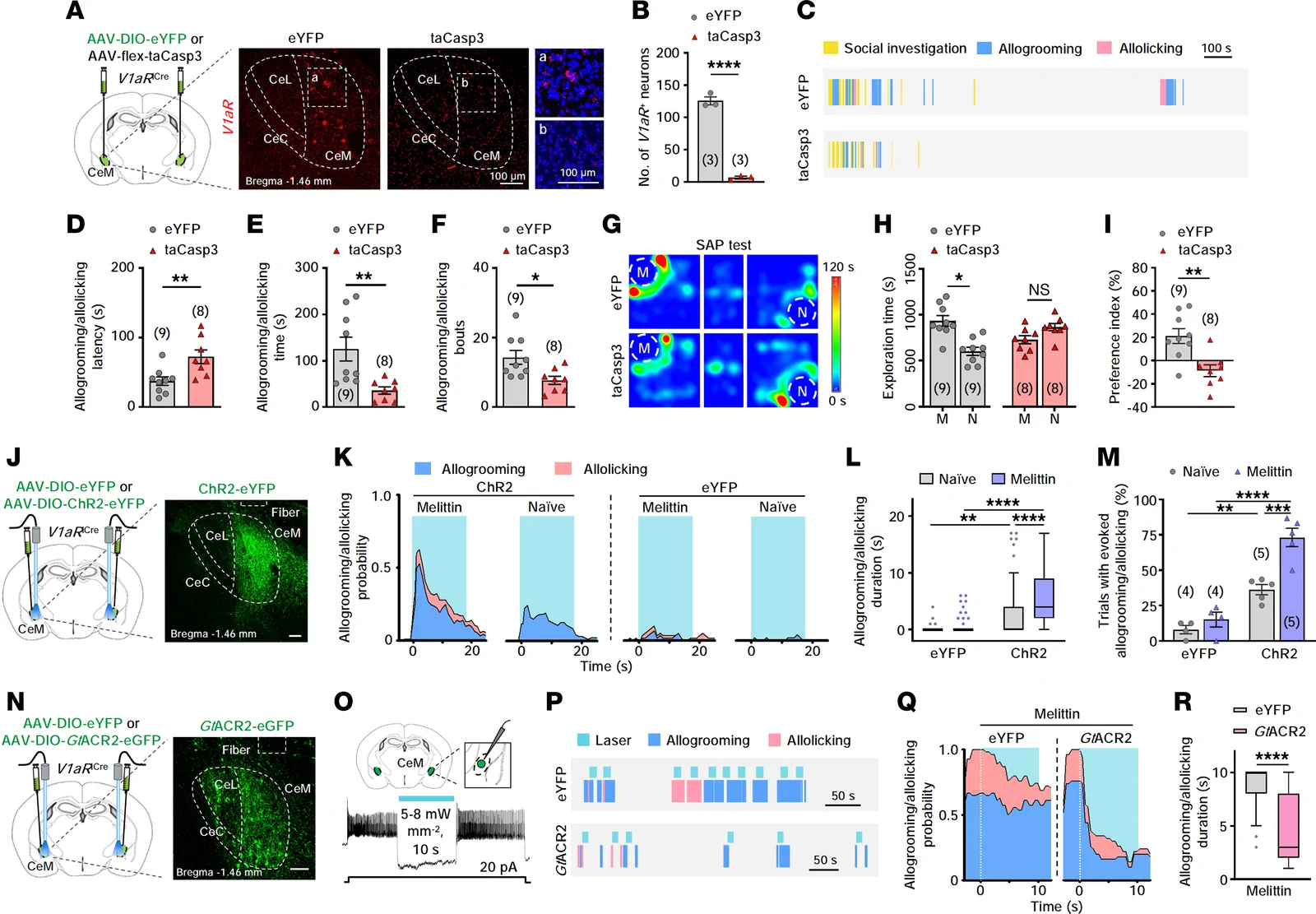
CeM^V1aR^ neurons drive general and targeted caregiving-like behavior. (**A** and **B**) Schematic of viral injections and representative images showing *V1aR* mRNA expression (**A**) and statistics (**B**). (**C**) Representative raster plots depicting social investigation, allogrooming, and allolicking in eYFP and taCasp3 mice during the prosocial interaction. (**D**–**F**) Latency (**D**), time (**E**), and bouts (**F**) of allogrooming/allolicking toward pain-suffering cagemates of both eYFP and taCasp3 observers. (**G**–**I**) Heatmaps of locomotor activity (**G**), exploration time (**H**), and preference index (**I**) of both eYFP and taCasp3 mice in social affective preference (SAP) test. (**J**) Schematic for CeM injections and fiber implantations (left), and a representative image showing ChR2-eYFP expression (right) in the CeM. (**K** and **L**) Probability (**K**) and duration (**L**) of evoked allogrooming/allolicking toward naive or melittin-treated cagemates during opto-activation (17 s) in eYFP versus ChR2 mice. (**M**) Percentage of trials in each eYFP and ChR2 mice with evoked allogrooming/allolicking during opto-activation. (**N**) Schematic for CeM injections and fiber implantations (left), and a representative image showing *Gt*ACR2-eGFP expressions (right) in the CeM. (**O**) Whole-cell voltage clamp showing light-mediated silencing of CeM^V1aR^ neurons in an acute slice preparation. (**P**–**R**) Example raster plots (**P**), probability (**Q**), and duration (**R**) of spontaneous allogrooming/allolicking toward pain-suffering cagemates during opto-inhibition (10 s) in eYFP and *Gt*ACR2 observers. Scale bar: 100 μm (**A**, **J** and **N**). All mice are male. *n* = 52–98 trials from 4–5 mice in **K–M** and **P–R**. Other *n* values are indicated in the figure. Statistical results are included in Supplemental Table 2. Mean ± SEM in **B**, **D**–**F**, **H**, **I** and **M**. Box and whiskers (center line, median; box limits, quartiles; whiskers, 1.5 × interquartile range; points, outliers) in **L** and **R**. Unpaired *t* test **B**, **D**, **F**, and **I**; Mann Whitney U test for **E** and **R**; Paired *t* test for **H**; Kruskal-Wallis test with Dunn’s multiple comparisons for **L**; 2-way ANOVA with Tukey’s multiple comparisons for **M**.

**Figure 5:**
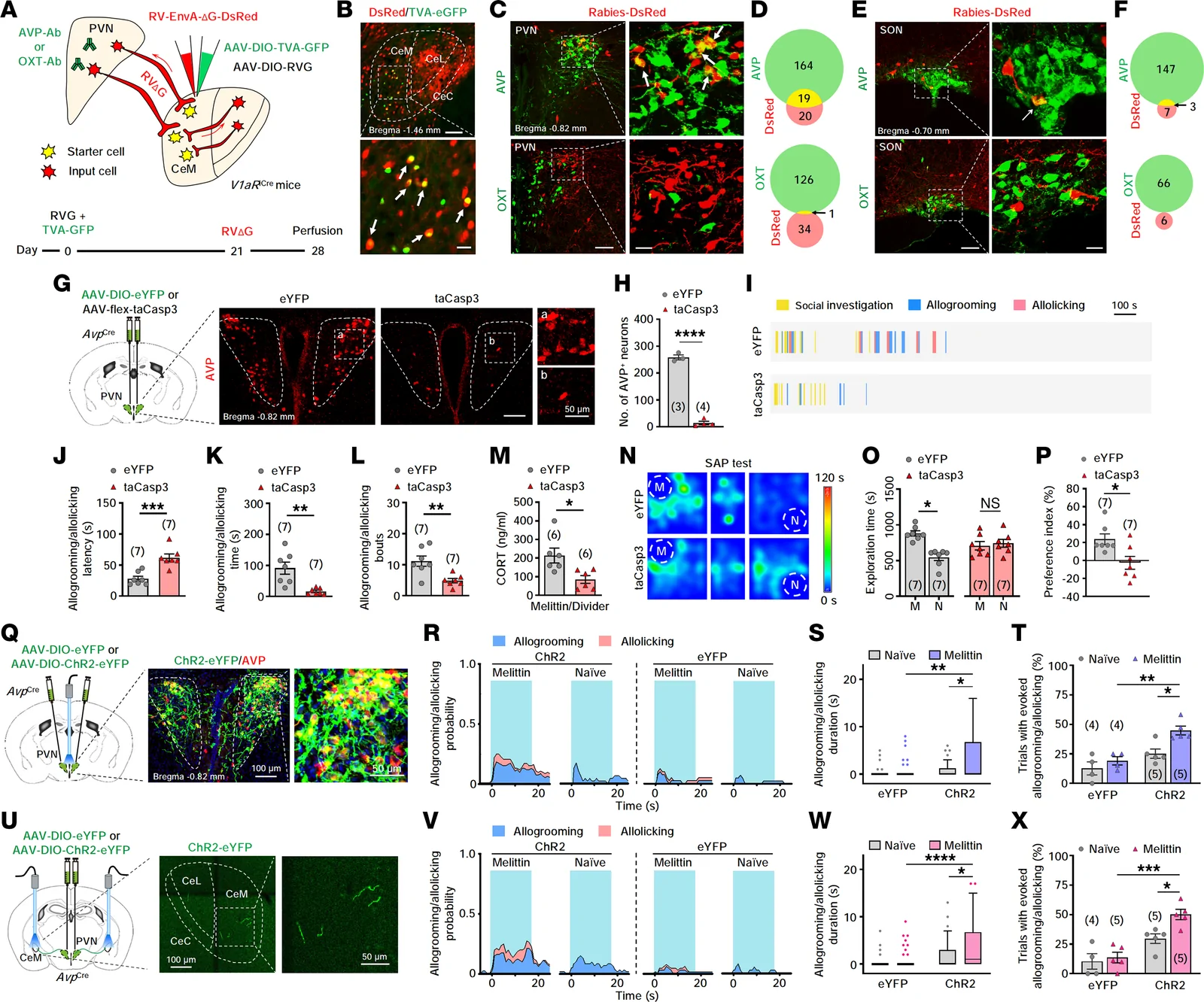
PVN^AVP^-CeM^V1aR^ neural pathway initiates caregiving for others in pain. (**A**) Schematic of rabies virus retrograde tracing of PVN^AVP^-CeM^V1aR^ monosynaptic projection. (**B**) Representative images of *V1aR*-expressing starter neurons in the CeM. (**C**–**F**) Representative images and venn diagrams showing colocalizations of rabies-DsRed with AVP (top) and OXT (bottom) in the PVN (**C** and **D**) and the SON (**E** and **F**), respectively. (**G** and **H**) Schematic of viral injections and representative images showing AVP expression in the PVN (**G**) and statistics (**H**). (**I**) Representative raster plots of social investigation, allogrooming, and allolicking in eYFP and taCasp3 mice during prosocial interaction. (**J**–**L**) Latency (**J**), duration (**K**), and bouts (**L**) of allogrooming/allolicking toward pain-suffering cagemates of both eYFP and taCasp3 observers. (**M**) Plasma CORT levels of eYFP and taCasp3 mice after 25–30 minutes social exposure to melittin-treated demonstrators in divider paradigm. (**N**–**P**) Heatmaps of locomotor activity (**N**), exploration time (**O**), and preference index (**P**) of both eYFP and taCasp3 mice in social affective preference (SAP) test. (**Q**) Schematic for PVN injections and fiber implantations (left), and a representative image showing ChR2-eYFP/AVP coexpression (right) in the PVN. (**R** and **S**) Probability (**R**) and duration (**S**) of evoked allogrooming/allolicking toward naive or melittin-treated cagemates during PVN opto-activation (17 s) in eYFP versus ChR2 mice. (**T**) Percentage of trials in each eYFP and ChR2 mice with evoked allogrooming/allolicking during PVN opto-activation (17 s). (**U**) Schematic for PVN injections and CeM fiber implantations (left), and a representative image showing ChR2-eYFP expression (right) in the CeM. (**V** and **W**) Probability (**V**) and duration (**W**) of evoked allogrooming/allolicking toward naive or melittin-treated cagemates during CeM opto-activation (17 s) in eYFP versus ChR2 mice. (**X**) Percentage of trials in each eYFP and ChR2 mice with evoked allogrooming/allolicking during CeM opto-activation (17 s). Arrows indicate double-labeled cells. Scale bar: 100 μm (original in **B**, **C**, **E**, **G**, **Q** and **U**), 50 μm (magnified in **G**, **Q** and **U**), 25 μm (magnified in **B**, **C**, and **E**). All mice are male. *n* = 3 mice in **C**–**F**; *n* = 38–92 trials from 4–5 mice in **R**–**T** and **V**–**X**. Other n values are indicated in the figure. Statistical results are included in Supplemental Table 2. Mean ± SEM in **H**, **J**–**M**, **O**, **P**, **T** and **X**. Box and whiskers (center line, median; box limits, quartiles; whiskers, 1.5 × interquartile range; points, outliers) in **S** and **W**. SON, supraoptic nucleus. Unpaired *t* test for **H**, **J**, **L**, and **M**; Unpaired *t* test with Welch’s correction for **K**; Wilcoxon matched-pairs test for **O** (eYFP); Paired *t* test for **O** (taCasp3); Mann Whitney U test for **P**; Kruskal-Wallis test with Dunn’s multiple comparisons for **S** and **W**; 2-way ANOVA with Tukey’s multiple comparisons for **T** and **X**.

**Figure 6:**
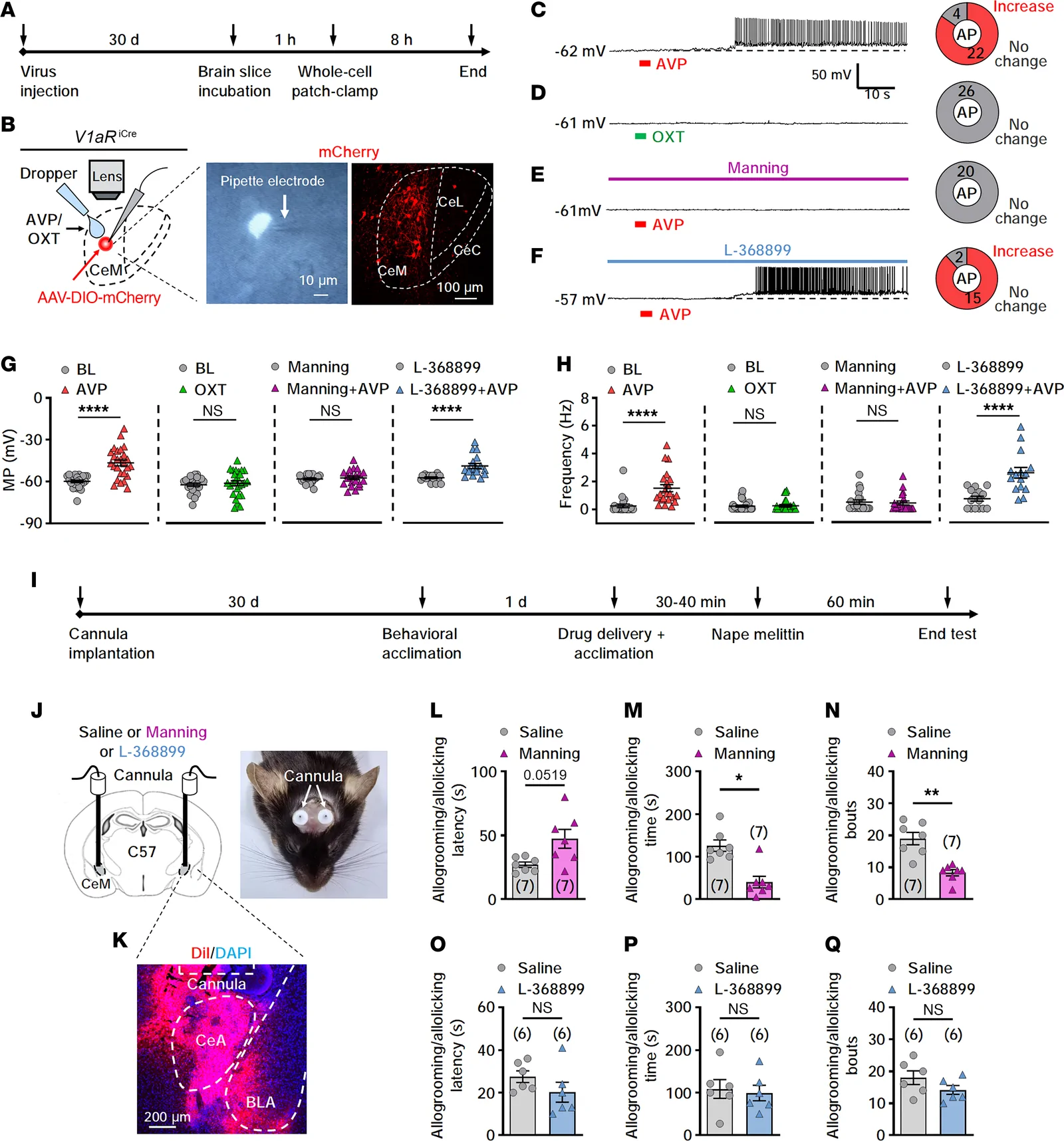
Selective activation of CeM^V1aR^ neurons by AVP-V1aR signaling in caregiving-like behavior. (**A** and **B**) Timeline (**A**) and schematic (**B**) of whole-cell patch-clamp recording on CeM *V1aR*^mCherry^ neurons in *V1aR*^iCre^ mice. (**C**–**F**) Representative traces of membrane depolarizations with action potentials (AP) (left) and incidences showing AP increase (right) of CeM *V1aR*^mCherry^ neurons before and after bath application of AVP (0.2 μM) (**C**), OXT (1 μM) (**D**), AVP + Manning (1 μM) (**E**), and AVP + L-368899 (1 μM) (**F**). (**G** and **H**) Membrane potentials (**G**) and firing rates (**H**) of CeM *V1aR*^mCherry^ neurons before and after bath application of AVP, OXT, AVP + Manning, and AVP + L-368899. (**I**) Timeline of pharmacological interventions in caregiving-like behavior test. (**J**) Schematic and a representative image showing cannula implantations. (**K**) Validation of cannula implantation above the CeA. (**L**–**N**) Latency (**L**), duration (**M**), and bouts (**N**) of allogrooming/allolicking toward pain-suffering cagemates of both saline- and Manning-treated observers. (**O**–**Q**) Latency (**O**), duration (**P**), and bouts (**Q**) of allogrooming/allolicking toward pain-suffering cagemates of both saline- and L-368899-treated observers. 5 mice were shared between 2 experiments in **L**–**N** and **O**–**Q**. Scale bars: 10 μm (**B**, middle); 100 μm (**B**, right); and 200 μm (**K**). *n* = 17–26 recorded cells in each group from 13 male and 10 female mice in **C**– **F**. Other *n* values are indicated in the figure. Statistical results are included in Supplemental Table 2. Data are mean ± SEM. Wilcoxon matched-pairs test for **G** (AVP, L368899+AVP), **H**, and **M**; Paired *t* test for **G** (OXT, Manning+AVP), **L**, **N**–**Q**.

**Figure 7:**
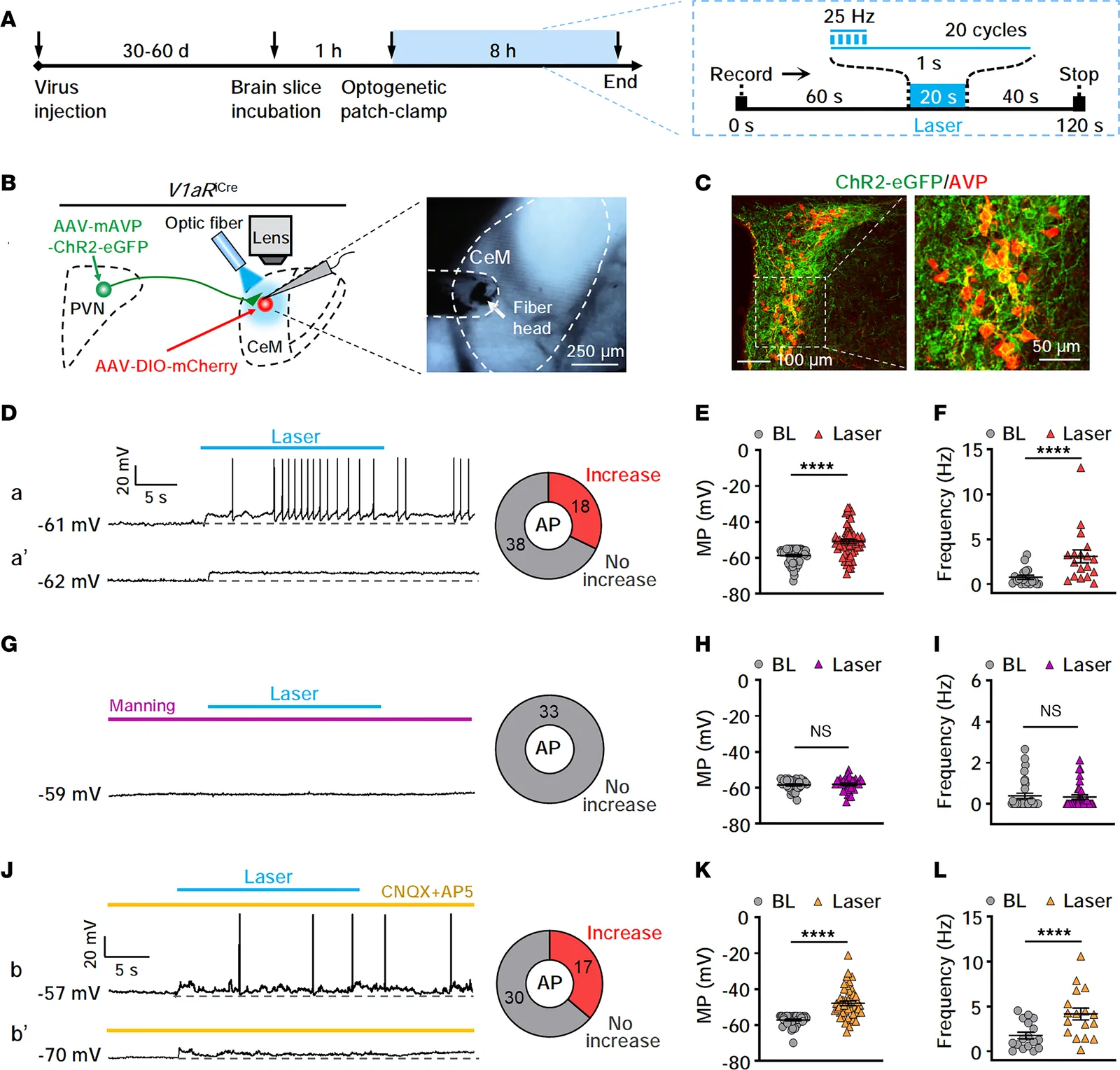
Direct activation of CeM^V1aR^ neurons by AVP release from the PVN. (**A**) Timeline (left) and protocols (right) for patch-clamp recording under burst opto-activation. (**B**) Schematic of whole-cell patch-clamp recording on CeM *V1aR*^mCherry^ neurons under opto-activation of PVN *AVP*^ChR2^ terminals in the CeM. (**C**) Representative images showing ChR2-eYFP/AVP coexpression in the PVN. (**D**) Representative traces of membrane depolarizations with (a) or without (a’) APs (left) and an incidence showing AP increase (right) of *V1aR*^mCherry^ neurons before and during opto-activation of CeM-projecting *AVP*^ChR2^ terminals. (**E** and **F**) Membrane potentials (**E**) and firing rates (**F**) of *V1aR*^mCherry^ neurons before and during opto-activation of CeM-projecting *AVP*^ChR2^ terminals. (**G**) A representative trace of membrane potential (left) and an incidence showing AP increase (right) of *V1aR*^mCherry^ neurons before and during opto-activation of CeM-projecting *AVP*^ChR2^ terminals in the presence of Manning. (**H** and **I**) Membrane potentials (**H**) and firing rates (**I**) of *V1aR*^mCherry^ neurons before and during opto-activation of CeM-projecting *AVP*^ChR2^ terminals in the presence of Manning. (**J**) Representative traces of membrane depolarizations with (b) or without (b’) APs (left) and an incidence showing AP increase (right) of *V1aR*^mCherry^ neurons before and during opto-activation of CeM-projecting *AVP*^ChR2^ terminals in the presence of both CNQX and AP5. (**K** and **L**) Membrane potentials (**K**) and firing rates (**L**) of *V1aR*^mCherry^ neurons before and during opto-activation of CeM-projecting *AVP*^ChR2^ terminals in the presence of both CNQX and AP5. Scale bars: 50 μm (**C**, right), 100 μm (**C**, left) and 250 μm (**B**, right). *n* = 33–56 recorded neurons from 10 male and 9 female mice. Statistical results are included in Supplemental Table 2. Data are mean ± SEM. Wilcoxon matched-pairs test for **E**, **F**, **H**, **I**, **K**, and **L**.

**Figure 8:**
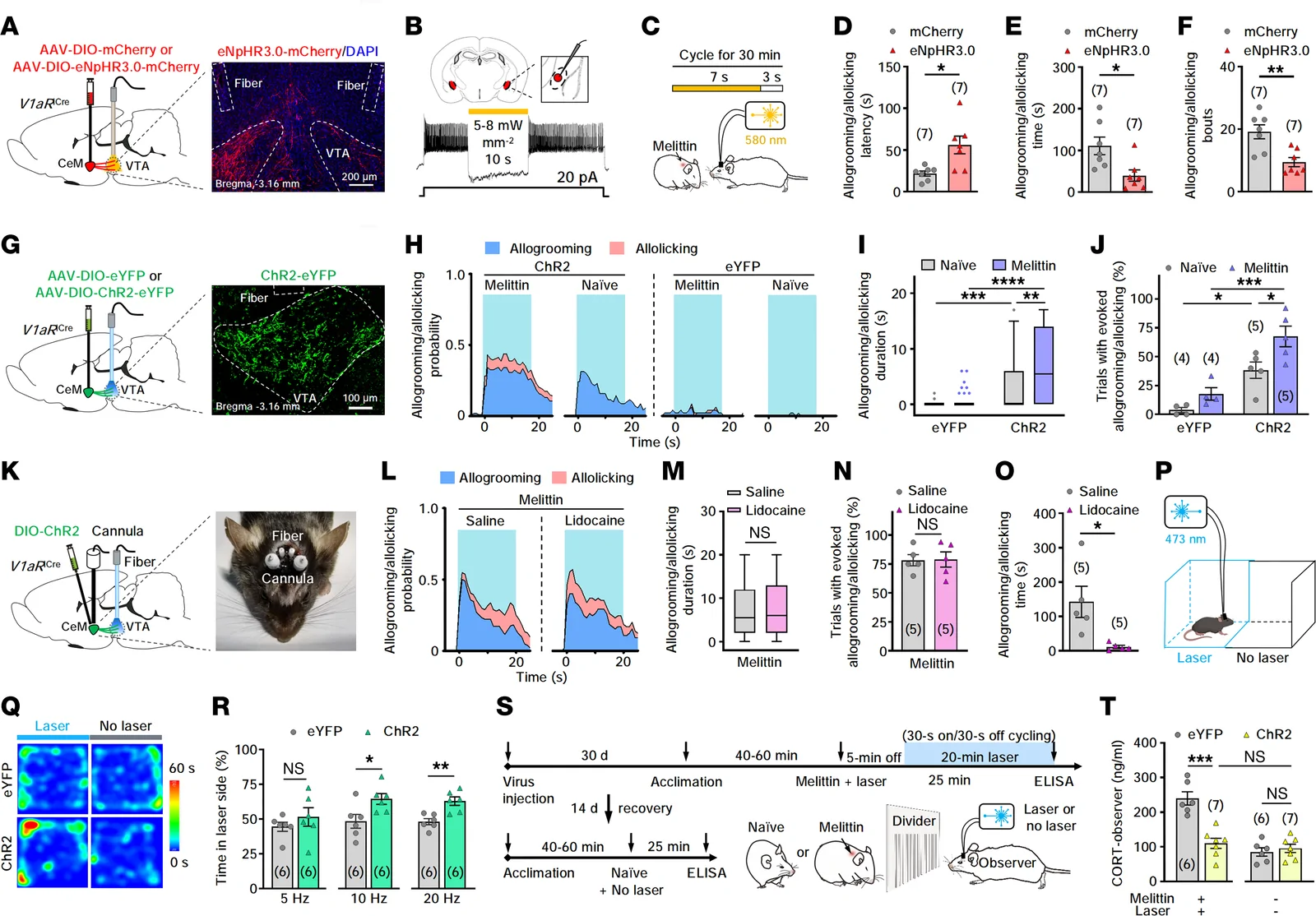
VTA-projecting CeM^V1aR^ neurons promote caregiving-like behavior. (**A**) Schematic of CeM injections and VTA fiber implantations (left), and a representative image showing eNpHR3.0-mCherry expression (right) in the VTA. (**B**) Whole-cell voltage clamp showing light-mediated silencing of CeM^V1aR^ neurons by eNpHR3.0. (**C**) Schematic for opto-inhibition during caregiving-like behavior test. (**D**–**F**) Latency (**D**), duration (**E**), and bouts (**F**) of allogrooming/allolicking toward pain-suffering cagemates under opto-inhibition in the VTA of mCherry and eNpHR3.0 mice. (**G**) Schematic of CeM injections and VTA fiber implantations (left), and a representative image showing ChR2-eYFP expression (right) in the VTA. (**H** and **I**) Probability (**H**) and duration (**I**) of evoked allogrooming/allolicking toward naive or melittin-treated cagemates during VTA opto-activation (17 s) in eYFP versus ChR2 mice. (**J**) Percentage of trials in each eYFP and ChR2 mice with evoked allogrooming/allolicking during VTA opto-activation. (**K**) Schematic and a representative image showing the viral injection, cannula, and fiber implantations in the CeA and VTA, respectively. (**L** and **M**) Probability (**L**) and duration (**M**) of evoked allogrooming/allolicking toward melittin-treated cagemates during VTA opto-activation (17 s) in ChR2 mice with preapplication of saline or lidocaine in the CeA. (**N**) Percentage of trials in each ChR2 mouse with evoked allogrooming/allolicking during VTA opto-activation with preapplication of saline or lidocaine in the CeA. (**O**) Allogrooming/allolicking time toward melittin-treated cagemates with preapplication of saline or lidocaine in the CeA during 60-minute prosocial interaction. (**P**) The setup of RTPP test. (**Q** and **R**) Heatmaps (20 Hz) of locomotor activity (**Q**) and percentage of time spent in the chamber paired with opto-activation (**R**) in the RTPP test. (**S**) Experimental design. Divider paradigm allows visual/olfactory/auditory exposure but bodily untouchable between the observer and demonstrator (which is either naive or melittin-treated). Timeline of the experiment: during the social interaction, CeM^V1aR^-VTA projection was optogenetically activated (or not activated) in observer mice expressing either ChR2 (experimental group) or eYFP (control group). Following the behavioral test, blood was collected from all observer mice for ELISA measurement. (**T**) Plasma CORT levels in eYFP and ChR2 observers after 25-minute social interaction in divider paradigm. Observer mice were exposed to either naive or melittin-treated cagemates, and CeM^V1aR^-VTA projection was optogenetically activated or not activated during the interaction. Scale bars: 100 μm (**G**), 200 μm (**A**). All mice are male. *n* = 48–114 trials from 4–5 mice in **H**–**J** and **L**–**O**. Other *n* values are indicated in the figure. Statistical results are included in Supplemental Table 2. Mean ± SEM in **D**–**F**, **J**, **N**, **O**, **R**, and **T**. Box and whiskers (center line, median; box limits, quartiles; whiskers, 1.5 × interquartile range; points, outliers) in **I** and **M**. Unpaired *t* test for **E**, **F**, **R**, and **T** (eYFP/Melittin/Laser versus ChR2/Melittin/Laser; eYFP/Naive/No laser versus ChR2/Naive/No laser); Unpaired *t* test with Welch’s correction for **D**; Kruskal-Wallis test with Dunn’s multiple comparisons for **I**; 2-way ANOVA with Tukey’s multiple comparisons for **J**; Wilcoxon matched-pairs test for **M**; Paired *t* test for **N**, **O**, and **T** (ChR2/Melittin/Laser versus ChR2/Naive/No laser).

## Version 1 10/01/2026

Electronic publication

## Version 2 10/02/2026

Corrected funding number to 82001180 (YY)
