# Supplementary material for: Hypothalamic-amygdala gating of caregiving-like prosocial behavior in mice
Source: J Clin Invest. 2026 Oct 1;136(19):e204317. doi: 10.1172/JCI204317 (PMC13626834; doi:10.1172/JCI204317)
Supplement: Supplementary file 1 [file jci-136-204317-s009.pdf]

Supplemental Materials for

Hypothalamic-amygdala gating of caregiving-like prosocial behavior in mice

Kai-Wen Geng[1]†*, Rui-Rui Wang[1]†, Yan Yang[1], Yan Wang[1], Ting He[1], Chun-Li Li[1], Chong-Shun Xu[1], and Jun Chen[1]‡*

[1]Institute for Biomedical Sciences of Pain, Tangdu Hospital, Fourth Military Medical University, Xi'an 710038, Shaanxi, P. R. China

‡Present address: Sanhang Institute for Brain Science and Technology, Northwestern Polytechnical University, Xi'an 710129, Shaanxi, P. R. China

†These authors contributed equally.

*e-mail: gengkw@fmmu.edu.cn (K.-W. G), junchen_sibst@nwpu.edu.cn (J. C.)

This file includes:

Supplemental Methods

Supplemental Figures 1 to 13

Captions for Supplemental Videos 1 to 7

Other Supplementary Material for this manuscript includes the following:

Supplemental Videos 1 to 7

Supplemental Tables 1 to 3

Supplemental Methods

Drugs and Reagents

Melittin (30 µl, 1 mg/mL, GenScript, s.c.) (31), clozapine (0.05 to 0.1 mg/kg, Tocris, i.p.) (89), CP154526 (10 mg/kg, Sigma-Aldrich, i.p.) (32), Manning (500 nl, 1 µM, Tocris, intra-cannula) (42), L-368899 (500 nl, 1 µM, Tocris, intra-cannula) (44), and lidocaine (500 nl, 0.1 mg/5 ml, Shanghai Harvest Pharmaceutical CO., intra-cannula) (5) were used as previously described. Bupivacaine (30 µl, 37.5 mg/5 ml, Shanghai Harvest Pharmaceutical CO., s.c.) was subcutaneously co-injected with melittin at a 1:1 volume ratio. *V1aR* siRNA (400 µl for each injection, Sigma-Aldrich) was incubated with RVG-9R peptide (GenScript) at a 1:1 volume ratio (end concentration 2 µg/µl) for 10 min and then delivered intracranially into the target site by neuroinjector (90).

Viruses

Adeno-associated viruses (AAV) used in this study were purchased from the BrainVTA Co., Ltd (Wuhan, China) and Taitool Bioscience (Shanghai, China), including AAV9-hSyn-DIO-hM4Di-eYFP, AAV9-Ef1α-DIO-hChR2-eYFP, AAV9-Ef1α-DIO-eYFP, AAV9-hSyn-DIO-st*Gt*ACR2-eGFP, AAV9-hSyn-DIO-jGCaMP7s, AAV9-hSyn-DIO-eNpHR3.0-mCherry, AAV9-Ef1α-flex-taCasp3, AAV9-mAVP:promotor-hChR2-eGFP, AAV9-hSyn-DIO-mCherry. Rabies virus (RV) was purchased from BrainVTA Co., Ltd (Wuhan, China), including RV-ENVA-ΔG-dsRed (2.0×10^8 IFU/mL), AAV9-Ef1α-DIO-RVG (2.0×10^{12} vg/ml), and AAV9-Ef1α-DIO-His-EGFP-2A-TVA (2.0×10^{12} vg/ml).

Plasma collection and ELISA assay

Immediately after the behavioral test (performed between 8:00-12:00 am), each mouse was anesthetized with isoflurane and underwent blood extraction from the cheek by syringe. The blood was quickly collected into heparin sodium-coated tubes placed on ice and centrifuged at 3000 rpm for 5 min at 4 ºC. The supernatant was collected and re-centrifuged at 12000 rpm for another 10 min at 4 ºC. The final supernatant was stored at -80 ºC until the assay. CORT concentrations were measured using ELISA kits (Enzo life Sciences, USA) following the manufacturer's protocols. The level of CORT was analyzed by Prism 8 (v8.3.1).

Real-time quantitative polymerase chain reaction (RT-qPCR)

RT-qPCR was performed as previously described (91). All samples were assayed with FastStart Universal SYBR Green Master (Roche) in duplicate. Data were acquired and analyzed using LightCycler 96 (Roche), and the expression of target mRNA was normalized to the expression of *Gapdh*. The following primers were used: *V1aR*: 5'- TGCTGGCGGTGATTTTCGT -3', 5'- GAGGTGTCGGATGAAGAGATGC -3', amplicon size, 111 bp; *Gapdh*: 5'- GCCTAGTTGGCTTCATCTCAAGG -3', 5'- TGAGGGCAAAGAGAGACCCATC -3'; amplicon size, 77 bp.

Immunohistochemistry (IHC)

Mice were intracardially perfused with 4% paraformaldehyde (PFA) in 0.01 M PBS. Brain tissues were then dissected and postfixed for 8 h and cryoprotected in 20% sucrose in PBS overnight at 4°C. Brain tissues were coronally sectioned (30 μm thickness) into 3 suites and stored in PBS until IHC. Free-floating sections containing target brain area from 1 of the 3 suites were placed in blocking buffer (0.4% Triton X-100 and 2% donkey serum in 0.01 M PBS) for 1 h at room

temperature. Afterwards, sections were incubated in primary antibodies overnight at 4°C, washed three times with PBS, incubated again in secondary antibodies for 3 h at room temperature, and washed another 3 times with PBS. After immunostaining, sections were mounted, coverslipped with FluoromountG (Southern Biotech) and imaged on a Nikon C2+ confocal microscope system (Nikon Instruments Inc.). The following primary antibodies were used in this study: rabbit anti-c-Fos (abcam), rabbit anti-OXT (Immunostar), rabbit anti-AVP (Immunostar), chicken anti-GFP (abcam), chicken anti-mCherry (abcam). The following secondary antibodies were used: Cy3-conjugated donkey anti-rabbit (1:1000, Jackson Immuno-Research), Cy3-conjugated donkey anti-chicken (1:1000, Jackson Immuno-Research), Alexa 488-conjugated donkey anti-rabbit (1:1000, Jackson Immuno-Research), and Alexa 488-conjugated donkey anti-chicken (1:1000, Jackson Immuno-Research). Images were analyzed by an observer blinded to the group or genotype using Image J software from NIH Image.

c-Fos IHC

For c-Fos staining, mice were perfused 90 min after the onset of the behavioral test. One of the 3 suites of each brain was randomly selected for c-Fos immunofluorescent staining and comparison. Brain anatomical regions were specified according to the brain morphology and DAPI staining. Images of the anterior cingulate cortex (ACC), bed nucleus of the stria terminalis (BNST), central amygdala (CeM), hippocampus (Hippo), insular cortex (IC), nucleus accumbens (NAc), periaqueductal gray (PAG), prelimibic cortex (PrL), hypothalamic paraventricular nucleus (PVN), ventral tegmental area (VTA) were collected using NanoZoomer slide scanning system (Hamamatsu Photonics Co., Japan) and Nikon C2+ confocal microscope system (Nikon Instruments Inc., Japan). The c-Fos counting was performed with the toolbox based on MATLAB.

The core algorithm of the toolbox is designed to recognize the c-Fos puncta based on the principle of topology and achieved by the *bwareaopen* function in MATLAB. Before automatic counting, all images of comparable regions were preprocessed using the same parameters to increase the contrast (default) and reduce the background (default). The contrast adjustment option in the toolbox utilizes the *imadjust* function of MATLAB to enable users to adjust the contrast intensity. The background adjustment option applies the *ImageHiPass* function in the toolbox to achieve high pass filtering with 2-D fast Fourier transform and inverse transform.

RNAscope in situ hybridization

Fluorescence in situ hybridization (FISH) was performed using the RNAscope kit (Advanced Cell Diagnostics, Cat. #323110). Briefly, mice were deeply anesthetized and perfused intracardially with 0.01 M diethylpyrocarbonate (DEPC) treated phosphate-buffered saline (0.1% DEPC-PBS) followed by 4% PFA in 0.01 M DEPC-PBS. The brain tissues were removed and postfixed with the same fixative solution for 24 hours at 4 °C, and then dehydrated in turn in 20% and 30% (w/v) sucrose in 0.01 M DEPC-PBS respectively at 4 °C. Each brain tissue was coronally sectioned into 3 suites with each section of 20 μm thickness using a cryostat microtome (Leica, Germany). Coronal brain sections containing CeM from 1 of the 3 suites were mounted onto SuperFrost Plus glass slides (Fisher Scientific, #12-550-15) and stored at -80 °C until use. On the test day, specified brain sections were sequentially processed with pretreatment steps including postfixation in 4% PFA in 0.01 M PBS for 15 min, hydrogen peroxide incubation for 10 min, target retrieval reagents (1 x) treatment at 100 °C for 5 min, dehydration in 50%, 75% and 100% ethanol for 2 min, respectively, and finally incubation with Protease III solution at 40 °C for 30 min. Sections were then incubated with the following target probes of mouse *V1aR* (418061-C1) and *iCre* (423321-

C3) at 40 °C for 2 h in a humidified oven (Isotemp, Thermo Fisher Scientific). These probes were diluted 50: 1: 1 (C1: C2: C3) based on their channel properties. Following probe hybridization, sections were rinsed in 1 x washing buffer twice, and then treated with a series of probe signal amplification steps including Amp 1 for 30 min, Amp 2 for 30 min and Amp 3 for 15 min at 40 °C with 1 x washing buffer rinses twice between each incubation. After signal amplification, fluorophores Opal 520 and Opal 570 were used to label the target probes. Sections were finally stained with DAPI for 5 min and coverslipped with FluoromountG (Electron Microscopy Sciences, 17984). Fluorescent images were obtained by Nikon C2+ confocal microscope system (Nikon Instruments, Inc., Japan) or FV3000 confocal microscope (Olympus, Japan), and analyzed using ImageJ software from NIH Image (version 1.34e). A cell was considered positive for a given mRNA transcript if it showed four or more fluorescent puncta and clusters for the corresponding channel (red or green) around DAPI-labeled nucleus and/or cytoplasm.

For characterization of the *V1aR* mRNA expression pattern in male C57 mice, 8 sequential brain slices containing CeM (from bregma -0.82 mm to -1.70 mm) were selected per mouse and 4 mice were counted. For validating *V1aR* siRNA knockdown in the CeM of C57 mice, CeM *V1aR*/*iCre* co-expression and CeM^{V1aR} neuronal ablation of $V1aR^{iCre}$ mice, 4 brain slices containing the CeM (from bregma -1.06 mm to -1.46 mm) were selected per mouse and 3 mice were counted for statistical analysis. For validating *V1aR* siRNA knockdown in the PVN or VTA of C57 mice, 2 brain slices containing the PVN (from bregma -0.82 mm to -0.94 mm) or VTA (from bregma -3.08 mm to -3.16 mm) were selected per mouse and 3 mice were counted for statistical analysis.

Stereotaxic surgeries

Mice were anesthetized with pentobarbital sodium and mounted on a stereotaxic device (RWD Life science Co., LTD). Intracranial injections of virus or siRNA into the CeM (AP -1.10 mm, ML ±2.85 mm, DV -4.60 mm from bregma), the PVN (AP -0.70 mm, ML ±0.22 mm, DV -4.9 mm from bregma) or the VTA (AP -3.15 mm, ML ±0.60 mm, DV -4.2 mm from bregma) were performed via a 1 μl neurosyringe (Hamilton) at a rate of 30 to 40 nL/min using a syringe pump (RWD). The needle was withdrawn 5-10 min after the infusion. The injection accuracy and the virus infectivity were confirmed by IHC or RNAscope. Optic fibers were implanted into the CeM (AP -1.10 mm, ML ±2.85 mm, DV -4.30 mm from bregma), the PVN (AP -0.70 mm, ML -0.10 mm, DV -4.5 mm from bregma, 2° angle), or the VTA (AP -3.15 mm, ML ±0.90 mm, DV -4.0 mm from bregma, ±2° angle) as needed. Cannulas were implanted into the CeM (AP -1.10 mm, ML ±2.80 mm, DV -4.30 mm from bregma) as needed.

For *V1aR* mRNA knockdown, C57 mice received bilateral injections of 300 nL *V1aR* siRNA or scrambled control into the CeM, PVN and VTA, respectively. Knockdown efficiency was validated within 9 days post-surgery.

For CeM^{V1aR} or PVN^{AVP} neuronal ablation, *V1aR*^{iCre} or *Avp*^{Cre} mice were bilaterally injected with 300 nL AAV9-Ef1α-flex-taCasp3 into the CeM and PVN, respectively.

For chemogenetic inhibition of CeM^{V1aR} neurons or PVN^{AVP} neurons, *V1aR*^{iCre} or *Avp*^{Cre} mice were bilaterally injected with 300 nL AAV9-hSyn-DIO-hM4Di-eYFP into the CeM and PVN, respectively.

For fiber photometry recording of CeM$^{\text{V1aR}}$ neurons, *V1aR*^{iCre} mice were unilaterally injected with 300 nL AAV9-hSyn-DIO-jGCaMP7s into the CeM. Optic fibers (⌀200-μm) were then implanted ~0.2 mm above the injection site.

For opto-activation of somas or CeM-projecting axonal terminals of PVN^{AVP} neurons, *Avp*^{Cre} mice were bilaterally injected with 300 nL AAV9-hSyn-DIO-hChR2-eYFP into the PVN. Optic fibers (⌀200-μm) were then implanted ~0.2 mm above the PVN or the CeM, respectively.

For opto-activation or opto-inhibition of CeM$^{\text{V1aR}}$ neurons, *V1aR*^{iCre} mice were bilaterally injected with 300 nL AAV9-hSyn-DIO-hChR2-eYFP or AAV9-hSyn-DIO-st*Gt*ACR2-eGFP into the CeM, respectively. Optic fibers (⌀200-μm) were then bilaterally implanted ~0.2 mm above the injection site.

For opto-activation or opto-inhibition of VTA-projecting axonal terminals of CeM$^{\text{V1aR}}$ neurons, *V1aR*^{iCre} mice were bilaterally injected with 300 nL AAV9-hSyn-DIO-hChR2-eYFP or AAV9-hSyn-DIO-eNpHR3.0-mCherry into the CeM, respectively. Optic fibers (⌀200-μm) were then bilaterally implanted ~0.2 mm above the VTA.

For opto-activation of VTA-projecting axonal terminals of CeM$^{\text{V1aR}}$ neurons with pre-delivering lidocaine into the CeA to avoid unwanted activation of collateral projections, *V1aR*^{iCre} mice were bilaterally injected with 300 nL AAV9-hSyn-DIO-hChR2-eYFP into the CeM. Optic fibers (⌀200-μm) and cannulas were then bilaterally implanted ~0.2 mm above the VTA and CeA, respectively.

For activating CeM projecting axonal terminals of PVN^{AVP} neurons during patch-clamp recording of CeM^{V1aR} neurons, $V1aR^{iCre}$ mice were bilaterally injected with 300 nL of AAV9-mAVP:Promotor-hChR2-eGFP and AAV9-hSyn-DIO-mCherry into PVN and CeM on the same day, respectively.

Chemogenetic experiments

Chemogenetic inhibition test was performed as described previously (90). On the test day, each observer was injected with clozapine (0.05 to 0.1 mg/kg, i.p.) and then acclimated in a transparent plastic testing box for 45 to 60 min. Each cagemate demonstrator with nape injection of melittin was then placed into the testing box. The entire 30-min dyadic social interaction was digitally recorded.

Optogenetic experiments

Opto-activation of ChR2 mice in somas and axonal terminals were performed using 473 nm blue laser at 10-20 Hz in 10 ms duration for around 17 s, with the intensity around 8-10 mW/mm^2. The effects on allogrooming/allolicking of soma and terminal activations were evaluated in the presence of naïve or melittin-treated cagemates. The effect on attack was evaluated only in the presence of melittin-treated cagemates. The effect on self-grooming was assessed in nonsocial context. For allogrooming/allolicking induction during opto-activation, laser stimulations were delivered when observers were attending to, approaching to or close to the demonstrators and in the periods without spontaneous allogrooming/allolicking. To count the probability of each specific behavior, trials from all mice were aligned to the onset of light delivery. The fraction of

trials showing that behavior at a given time point was defined as the probability of showing that behavior. Allogrooming/allolicking duration was defined as the actual time of evoked allogrooming/allolicking confined within laser illumination.

Opto-inhibition experiment was conducted as previously described (92, 93). Mice were connected to 470 or 580 nm laser (SLOC LASERS, Shanghai Laser & Optics Century Co.) via optical patch cords (⌀200-µm, Doric Lenses) and then placed into the testing box for 1 h acclimation. For opto-inhibition of VTA-projecting axonal terminals using eNpHR3.0, 580 nm laser was delivered continuously with 7 s on and 3 s off to the mice at final output powers of 8-10 mW/mm^2 during 30-min social interaction with melittin-treated demonstrators. For real-time suppression of allogrooming or allolicking using *Gt*ACR2, 10 s of 470 nm continuous illumination at 8-10 mW/mm^2 was triggered manually when mice were performing spontaneous allogrooming or allolicking. The social interactions with opto-inhibition were digitally recorded for behavioral analysis.

Real-time place preference (RTPP) test was performed as previously described (70). Custom-made two-chamber conditioning apparatus (70 cm × 35 cm × 30 cm) was randomly assigned with or without laser. *V1aR*^{iCre} mice bilaterally expressing ChR2 or eYFP were connected to a 473 nm laser via dual fiber optic patchcords (⌀200-µm) and then placed into the no-laser chamber at the onset of each experiment. Mice were allowed to freely explore between the two chambers for 10 min before test. Entry into and remaining in the laser-paired chamber triggered constant laser pulses at either 5 Hz, 10 Hz or 20 Hz (473 nm, 10 ms pulse width, 8 to 10 mW light power). Entry into the no-laser chamber ended the laser pulses. The chamber paired with

laser was counterbalanced across all mice. Time spent in each chamber within the entire 20-min trials were recorded via a digital camera (Canon) and measured using ANYMAZE 10 (Stoelting). RTPP was defined by the percentage of time spent in the laser-paired side on the total duration of each test.

The neural circuit underlying the anxiolysis in observer mice was studied in divider paradigm with opto-activation. On the test day, each observer mouse was connected to a 470 nm laser and acclimated in the right half of the testing box with a grid divider for 40-60 min. A cagemate demonstrator with nape melittin injection was then placed in the left half of the box. After a 5-min social interaction to induce stress state-matching, the observer mouse received 470 nm laser stimulation (30 s on/30 s off cycling, 8-10 mW/mm^2) for 20 min. Following the total 25-min social interaction, each observer mouse was anesthetized for immediate blood collection from cheek. After a 2-week recovery, each observer mouse connected to the optical cable was re-acclimated with a grid divider for 40-60 min and exposed to a naïve cagemate demonstrator for 25 min without laser stimulation. Blood samples from all observer mice were collected for further analysis. The concentration of plasma corticosterone (CORT) levels was measured using an ELISA kit (Enzo Life Sciences, USA), and data were analyzed using Prism 8.

Retrograde tracing of monosynaptic contacts

To examine direct innervations onto CeM^{V1aR} neurons by PVN^{AVP} neurons, rabies virus (RV) dependent monosynaptic retrograde tracing was used. $V1aR^{iCre}$ mice were first unilaterally injected with 300 nL of AAV9-Ef1α-DIO-RVG and AAV9-Ef1α-DIO-His-EGFP-2A-TVA at 1:1 volume ratio into the CeM, followed by the second injections of 300 nL RV-ENVA-ΔG-dsRed into the

same site 4 weeks later. One more week later, the mice were perfused and sectioned (3 sections of the PVN per mouse) for immunofluorescent experiments with AVP or OXT antibody for further identifications.

Fiber photometry recording

Fiber photometry recording was carried out using a commercial device (RWD Life Science) as previously described (94). Laser intensities for 470 nm and 410 nm beams were measured and adjusted to 30 μW (470 nm) and 15 μW (410 nm); the 410 nm wavelength band was used for motion control. Before the formal test, the GCaMP mice were connected to the LED lights for previewing and placed into the testing box for 40-60 min acclimation. For each formal test, a 5-min baseline was recorded, followed by introducing a naïve or pain-suffering demonstrator for another 10-20 min recording. GCaMP and control signals were collected by the camera at 20 Hz. The raw value F was compensated as F_{470}/F_{410}, and then used for calculating $\Delta F/F=(F-F_0)/F_0$, where F_0 is the median of raw signal. The dynamics of Ca^{2+} responses during social investigation, allogrooming, allolicking and self-grooming were analyzed for GCaMP mice respectively. The baseline signal was made using the median of the fluorescence values during the baseline period (-2 to -1 s relative to the onset of a specific behavior). To visualize the Ca^{2+} dynamics, the $\Delta F/F$ values of different behavioral states were heat plotted. To quantify the Ca^{2+} changes before and during a specific behavior, fluorescence values of the baseline period and the behavior period (0 to 3 s after the onset of allogrooming, allolicking and self-grooming; 0 to 5 s after the onset of social investigation) were comparatively analyzed, respectively.

Brain Slice Preparation and Whole-Cell Patch-Clamp Recording

Acute brain slices (400 µm thickness) were prepared from 3-5 months old mice of either sex in equal use. Slices were cut in ice-cold solution containing (in mM) (95): 92 NMDG, 2.5 KCl, 1.25 NaH_2PO_4, 30 $NaHCO_3$, 20 HEPES, 25 Glucose, 2 Thiourea, 5 Na-ascorbate, 3 Na-pyruvate, 0.5 $CaCl_2$ and 10 $MgCl_2{\cdot}6H_2O$ (pH 7.4) using a vibratome (DTK-1000, Dosaka EM. Co., Ltd, Japan). Slices were allowed to recover at room temperature for 30-60 min in a solution containing (in mM) (95): 92 NaCl, 2.5 KCl, 1.25 NaH_2PO_4, 30 $NaHCO_3$, 20 HEPES, 25 Glucose, 2 Thiourea, 5 Na-ascorbate, 3 Na-pyruvate, 2 $CaCl_2$ and 2 $MgCl_2{\cdot}6H_2O$ (pH 7.4), equilibrated with 95% O_2 and 5% CO_2. After recovery, slices were transferred to a recording chamber and incubated in artificial cerebrospinal fluid (ACSF) containing (in mM) (95): 119 NaCl, 2.5 KCl, 1.25 NaH_2PO_4, 24 $NaHCO_3$, 12.5 Glucose, 2 $CaCl_2$ and 2 $MgCl_2{\cdot}6H_2O$ (pH 7.4), equilibrated with 95% O_2 and 5% CO_2.

Whole-cell patch-clamp recordings were performed on CeM $V1aR^{\mathrm{mCherry}}$ neurons of $V1aR^{\mathrm{iCre}}$ mice (1 month for mCherry expression). $V1aR^{\mathrm{mCherry}}$ neurons were visualized using epifluorescence followed by infrared gradient contrast optical system combined with a monochrome CCD camera and monitor. During current clamp mode recordings, slices were continuously immersed in circulating oxygenated ASCF at a flow rate of 2.2 - 2.6 mL/min. Patch pipettes were pulled from borosilicate glass capillaries (outer diameter 1.5 mm, inner diameter 0.86 mm, SUTTER INSTRUMENT, Novato, CA) using a P-97 micropipette puller (SUTTER INSTRUMENT, Novato, CA) and had resistances ranging from 4 to 7 MΩ when filled with pipette solution containing (in mM): 120 K^+ gluconate, 18 KCl, 1 $CaCl_2$, 2 $MgCl_2$, 5 EGTA, 10 HEPES and 5 Mg-ATP (pH 7.3 with KOH; 290-300 mOsm). Pipettes were connected via an Ag-AgCl electrode to the headstage of an EPC-10 amplifier (HEKA, Lambrecht, Germany). AVP

(0.2 µM) (42), OXT (1 µM) (96), Manning (1 µM, selective V1aR antagonist) (42), and L-368899 (1 µM, selective OXTR antagonist) (44) were dissolved in ACSF and administered using gravity by flow pipe directed toward the recorded neuron or through bath application, respectively. AVP was applied at 15 min intervals between two recorded neurons in the same brain slice to avoid tachyphylaxis during recordings. Manning and L-368899 were continuously perfused for 10 min prior to subsequent drug applications.

To activate CeM projecting axonal terminals of PVN^{AVP} neurons during patch-clamp recording of CeM $V1aR^{mCherry}$ neurons, each $V1aR^{iCre}$ mouse was injected with AAV9-mAVP:Promotor-hChR2-eGFP into the PVN and AAV9-hSyn-DIO-mCherry into the CeM. 1-2 months later, the brain slices containing CeM were harvested for experiments. During the recording, the head of optical fiber was applied close to the edge of CeM with 20 degrees angle for laser delivery which allowed CeM illumination under a 40x water immersion objective simultaneously. After $V1aR^{mCherry}$ neurons were patched under current-clamp mode, CeM projecting axonal terminals were burst-like activated by 473 nm blue laser (five 4 ms pulses at 25 intra-burst frequency, repeatedly delivered at 1 Hz for 20 s, 5 mW) (97) and the firing responses of $V1aR^{mCherry}$ neurons were synchronously recorded. Manning (1 µM) (42), CNQX (20 µM) (46), and AP5 (50 µM) (46) were dissolved in ACSF and administered through bath application as needed.

Recordings in which the access resistance changed > 20% during the experiment and neurons with initial resting membrane potential (RMP) more depolarized than -55 mV were excluded from the analysis. Data were acquired with a HEKA 10.0 acquisition system using FitMaster software and further analyzed using MiniAnalysis (Synaptosoft, USA).

Reference

89. Gomez JL, Bonaventura J, Lesniak W, Mathews WB, Sysa-Shah P, Rodriguez LA, et al. Chemogenetics revealed: DREADD occupancy and activation via converted clozapine. *Science.* 2017;357(6350):503-7.
90. Kumar P, Wu H, McBride JL, Jung K-E, Kim MH, Davidson BL, et al. Transvascular delivery of small interfering RNA to the central nervous system. *Nature.* 2007;448(7149):39-43.
91. Meng QT, Liu XY, Liu XT, Liu J, Munanairi A, Barry DM, et al. BNP facilitates NMB-encoded histaminergic itch via NPRC-NMBR crosstalk. *Elife.* 2021;10.
92. Madisen L, Mao T, Koch H, Zhuo JM, Berenyi A, Fujisawa S, et al. A toolbox of Cre-dependent optogenetic transgenic mice for light-induced activation and silencing. *Nature neuroscience.* 2012;15(5):793-802.
93. Yang B, Karigo T, and Anderson DJ. Transformations of neural representations in a social behaviour network. *Nature.* 2022;608(7924):741-9.
94. Li YD, Luo YJ, Chen ZK, Quintanilla L, Cherasse Y, Zhang L, et al. Hypothalamic modulation of adult hippocampal neurogenesis in mice confers activity-dependent regulation of memory and anxiety-like behavior. *Nature neuroscience.* 2022;25(5):630-45.
95. Ting JT, Daigle TL, Chen Q, and Feng G. Acute brain slice methods for adult and aging animals: application of targeted patch clamp analysis and optogenetics. *Methods in molecular biology (Clifton, NJ).* 2014;1183:221-42.
96. Tang Y, Chen Z, Tao H, Li C, Zhang X, Tang A, et al. Oxytocin activation of neurons in ventral tegmental area and interfascicular nucleus of mouse midbrain. *Neuropharmacology.* 2014;77:277-84.
97. Pagani M, Albisetti GW, Sivakumar N, Wildner H, Santello M, Johannssen HC, et al. How Gastrin-Releasing Peptide Opens the Spinal Gate for Itch. *Neuron.* 2019;103(1):102-17.e5.

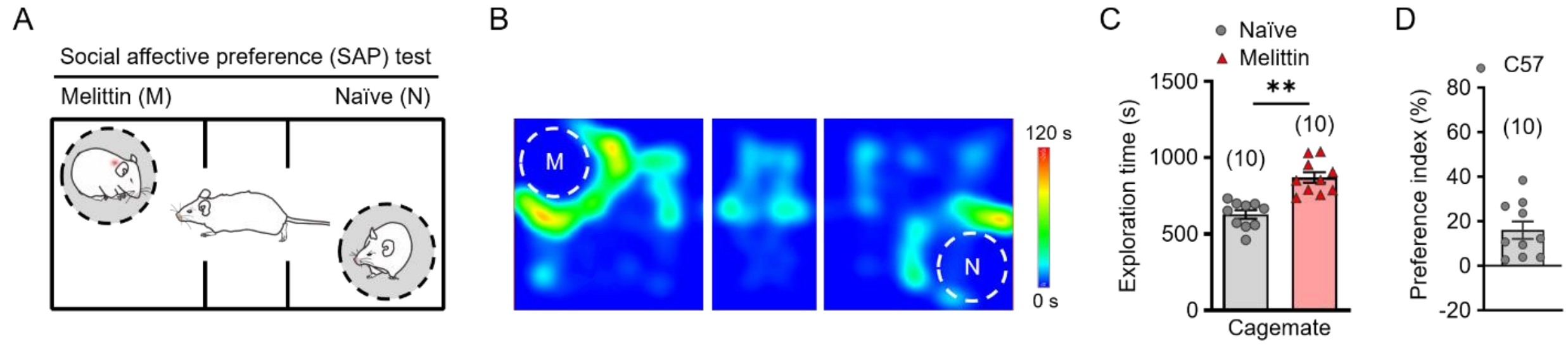

Supplemental Figure 1. Performances of male C57 mice in social affective preference (SAP) test. (**A**) Schematic of SAP test. (**B**-**D**) Heatmap of locomotor activity (B), exploration time (C) and preference index (D) of C57 male mice in SAP test. n = 10 mice for C and D. The statistical result is included in Table S3. Data are mean ± SEM. M, melittin; N, naïve.

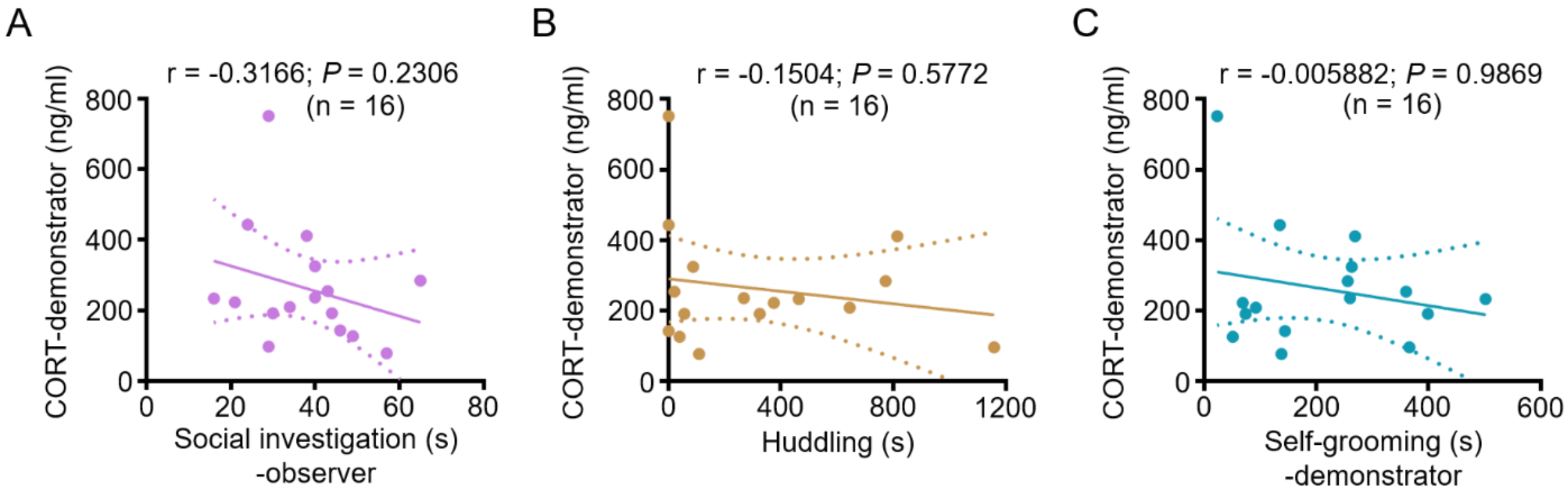

Supplemental Figure 2. Correlations between demonstrators' CORT levels and their own behaviors, and behaviors received from observers during the prosocial interaction. (A-C) Correlations between the demonstrators' plasma CORT levels and the duration of social investigation (A), huddling (B) and self-grooming (C) after 30-min prosocial interactions. A-C, Solid lines: linear regression; dashed lines: 95% confidence intervals. All mice are male. n = 16 observer-demonstrator pairs in A-C. Statistical results are included in Table S3.

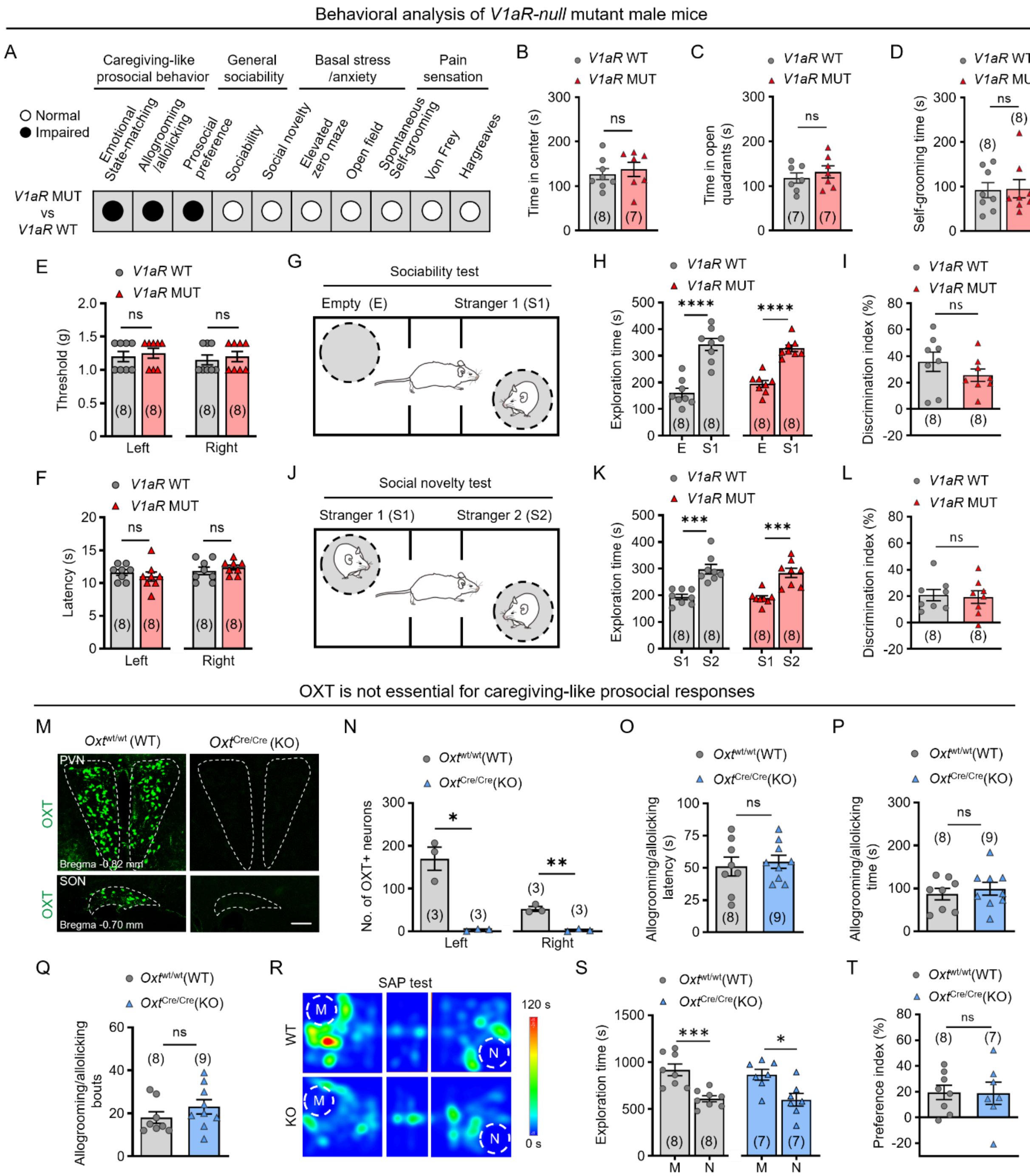

Supplemental Figure 3. Behavioral features of *V1aR-null* and *OXT-null* mutant male mice. (**A**) A form showing behavioral phenotypes of *V1aR-null* mutant mice. (**B**-**L**) Performances of *V1aR* WT and *V1aR* MUT mice in open field (B), elevated zero maze (C), spontaneous self-grooming in non-social context (D), acute pain (E, F), sociability (G-I) and social novelty tests (J-

L). (**M** and **N**) OXT expression (M) and quantifications (N) in both PVN and SON of *Oxt*$^{wt/wt}$ (WT) and *Oxt*$^{Cre/Cre}$ (KO) mice, respectively. (**O**-**Q**) Latency (O), duration (P) and bouts (Q) of allogrooming/allolicking toward melittin-treated cagemates in *Oxt*$^{wt/wt}$ (WT) and *Oxt*$^{Cre/Cre}$ (KO) mice. (**R**-**T**) Heatmaps of locomotor activity (R), exploration time (S) and preference index (T) of *Oxt*$^{wt/wt}$ (WT) and *Oxt*$^{Cre/Cre}$ (KO) mice in SAP test. Scale bar, 100 μm. Exact n values are indicated in the figure. All experimental mice are cagemates in M-T. Statistical results are included in Table S3. Data are mean ± SEM.

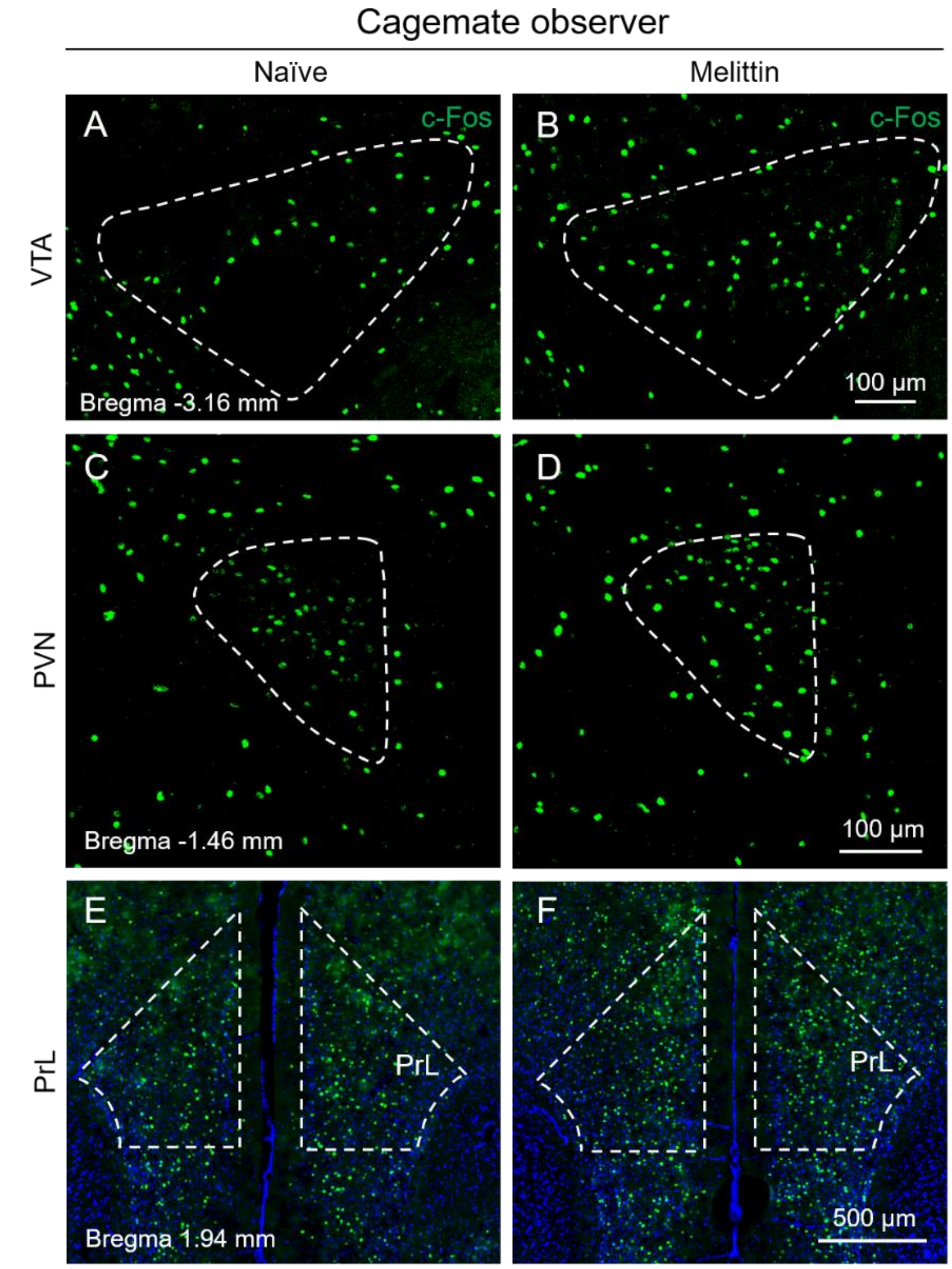

Supplemental Figure 4. c-Fos expressions in cagemate observers. (**A**-**F**) Representative images showing c-Fos expressions in the VTA, PVN and PrL of observer mice interacting with a naïve or melittin-treated cagemate, respectively. Scale bar, 100 µm in A-D; 500 µm in E, F.

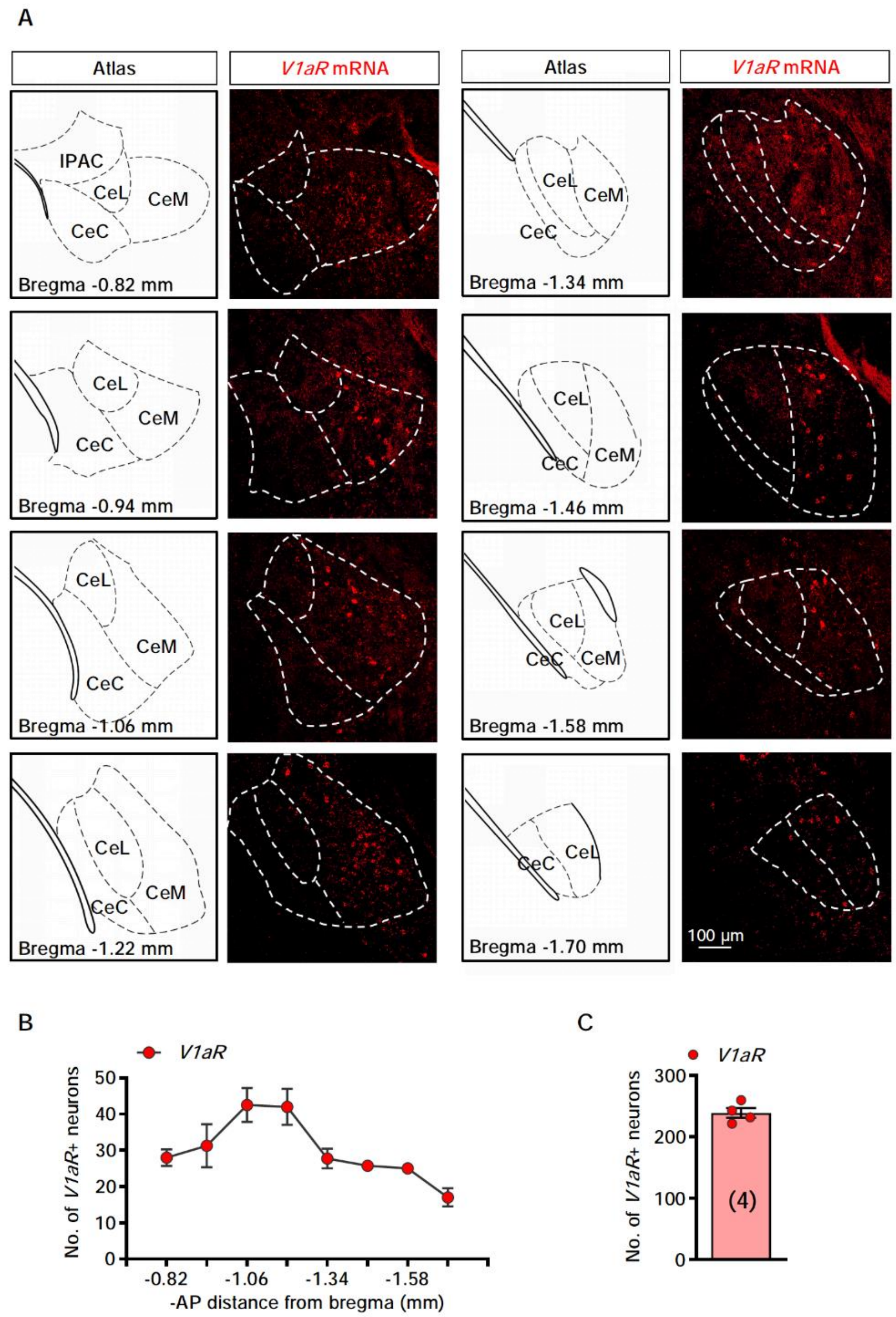

Supplemental Figure 5. *V1aR* mRNA distributions in the CeM of male C57 mice. (**A**) Representative images showing *V1aR* mRNA expression in CeM from rostral to caudal. (**B** and **C**) Mean number (B) and quantification (C) of *V1aR*-expressing neurons at each section containing CeM of male C57 mice. Scale bar, 100 μM. n = 4 mice for B and C. Data are mean ± SEM.

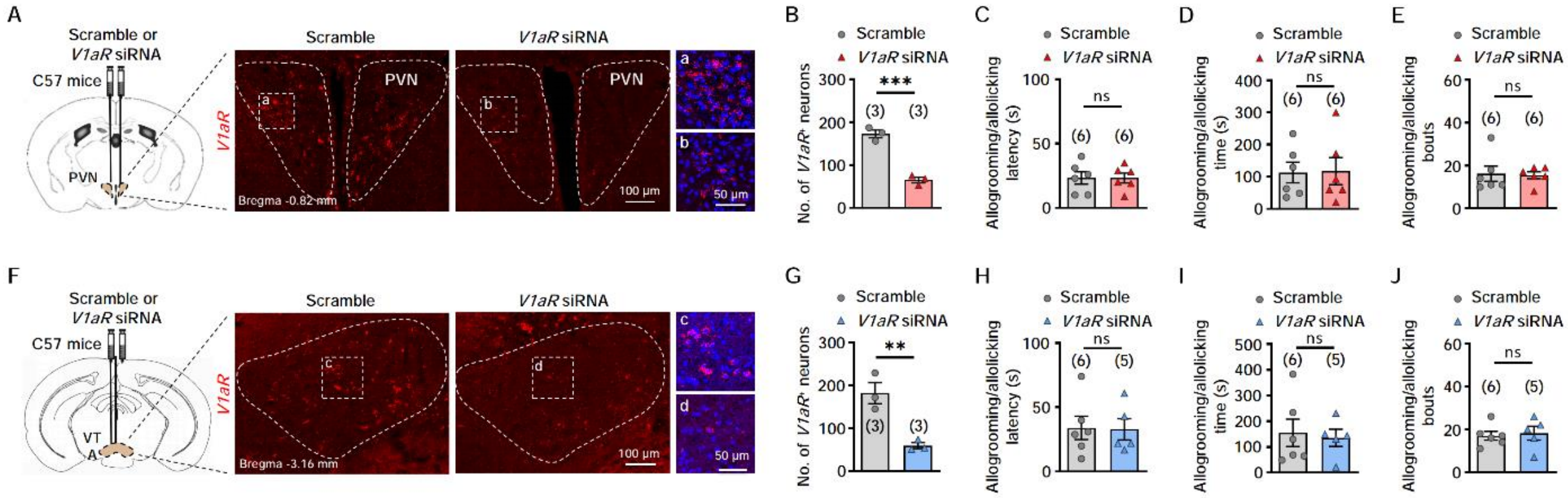

Supplemental Figure 6. V1aR signaling in the PVN or VTA is not required for caregiving-like behavior. (**A** and **B**) Schematic of PVN injections with scramble or *V1aR* siRNA (left), followed by measurements of *V1aR* mRNA expression (A) and statistics (B). (**C**-**E**) Latency (C), duration (D) and bouts (E) of allogrooming/allolicking toward pain-suffering cagemates of control and PVN *V1aR* knockdown observers. (**F** and **G**) Schematic of VTA injections with scramble or *V1aR* siRNA (left), followed by measurements of *V1aR* mRNA expression (F) and statistics (G). (**H**-**J**) Latency (H), duration (I) and bouts (J) of allogrooming/allolicking toward pain-suffering cagemates of control and VTA *V1aR* knockdown observers. Scale bars, 100 µm (original in A and F), 50 µm (magnified in A and F). Data are mean ± SEM. All mice are male. Exact n values are indicated in the figure. Statistical results are included in Table S3.

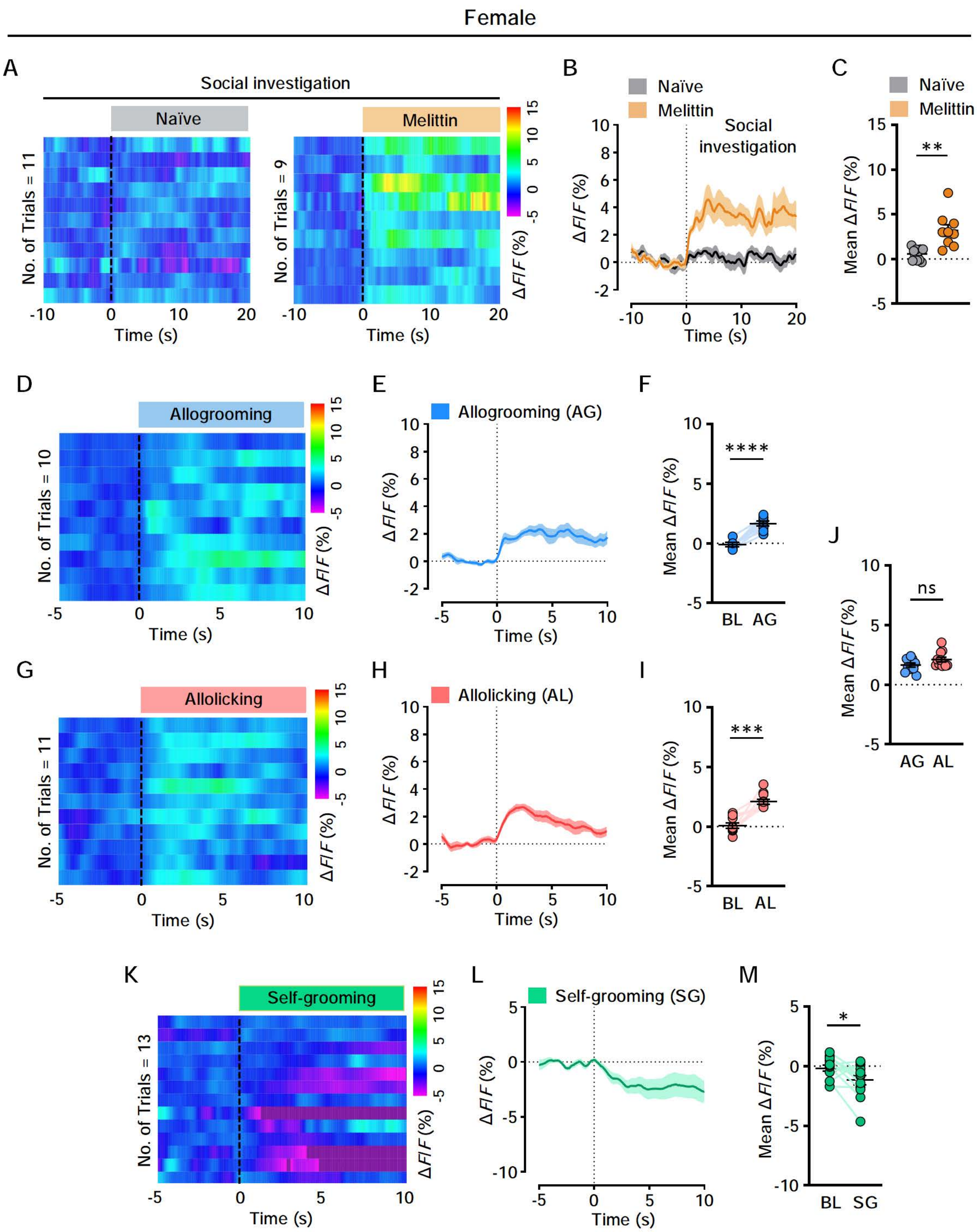

Supplemental Figure 7. Ca^{2+} responses of CeM^{V1aR} neurons during different behavioral states in females. (**A-C**) Heatmaps (A), average traces (B), and mean $\Delta F/F$ (C) showing Ca^{2+} dynamics of *V1aR*^{jGCaMP7s} neurons in response to naïve and melittin-treated cagemates. (**D-F** and

G-I) Heatmaps (D, G), average trace (E, H), and mean $\Delta F/F$ (F, I) showing Ca^{2+} dynamics of *V1aR*^{jGCaMP7s} neurons before (baseline) and during allogrooming (D-F) or allolicking (G-I) episodes. (**J**) Comparison of mean $\Delta F/F$ Ca^{2+} signals in *V1aR*^{jGCaMP7s} neurons during allogrooming and allolicking episodes. (**K-M**) Heatmap (K), average trace (L), and mean $\Delta F/F$ (M) showing Ca^{2+} dynamics of *V1aR*^{jGCaMP7s} neurons before (baseline) and during self-grooming episodes. Data are mean ± SEM. n = 9 or 11 trials from 4 mice in A-C; n = 10 trials from 3 mice in D-F; n = 11 trials from 3 mice in G-I; n = 13 trials from 3 mice in K-M. Statistical results are included in Table S3.

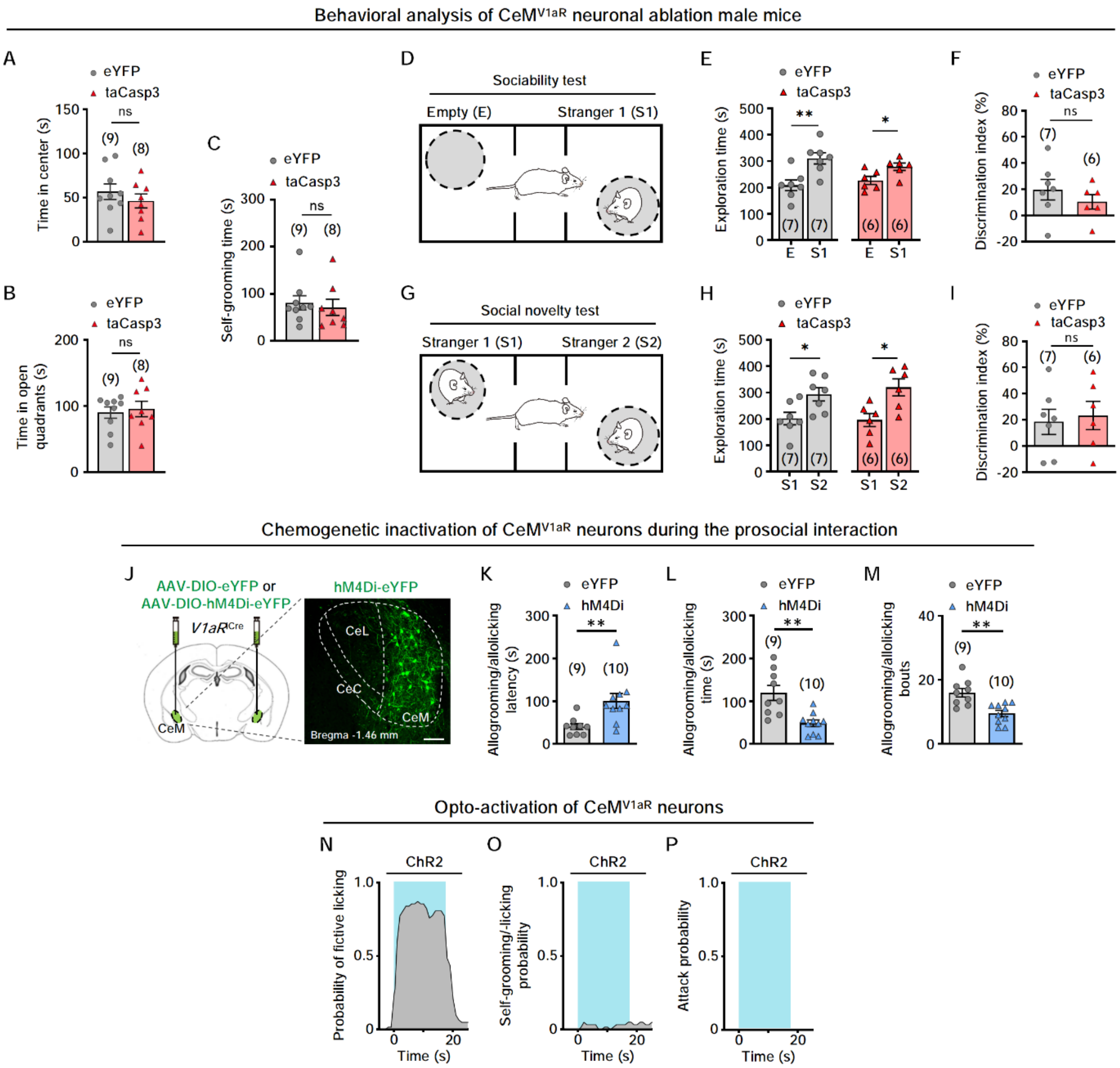

Supplemental Figure 8. Behavioral phenotypes of CeM^{V1aR} neuronal manipulations in males. (**A-I**) Performances of eYFP and taCasp3 mice in open field (A), elevated zero maze (B), spontaneous self-grooming in non-social context (C), sociability (D-F) and social novelty tests (G-I). (**J**) Schematic of CeM injections and a representative image showing hM4Di-eYFP expression. (**K-M**) Latency (K), duration (L) and bouts (M) of allogrooming/allolicking toward pain-suffering cagemates after clozapine injection in eYFP and hM4Di observers. (**N-P**) Behaviors induced by opto-activation (17 s) of CeM^{V1aR} neurons in ChR2 mice in non-social context (N, O) and social

context with melittin-treated cagemates (P). Scale bar, 100 μm. Exact n values of A-M are indicated in the figure; n = 62-77 trials from 4-5 mice for N-P. Statistical results are included in Table S3. Data are mean ± SEM.

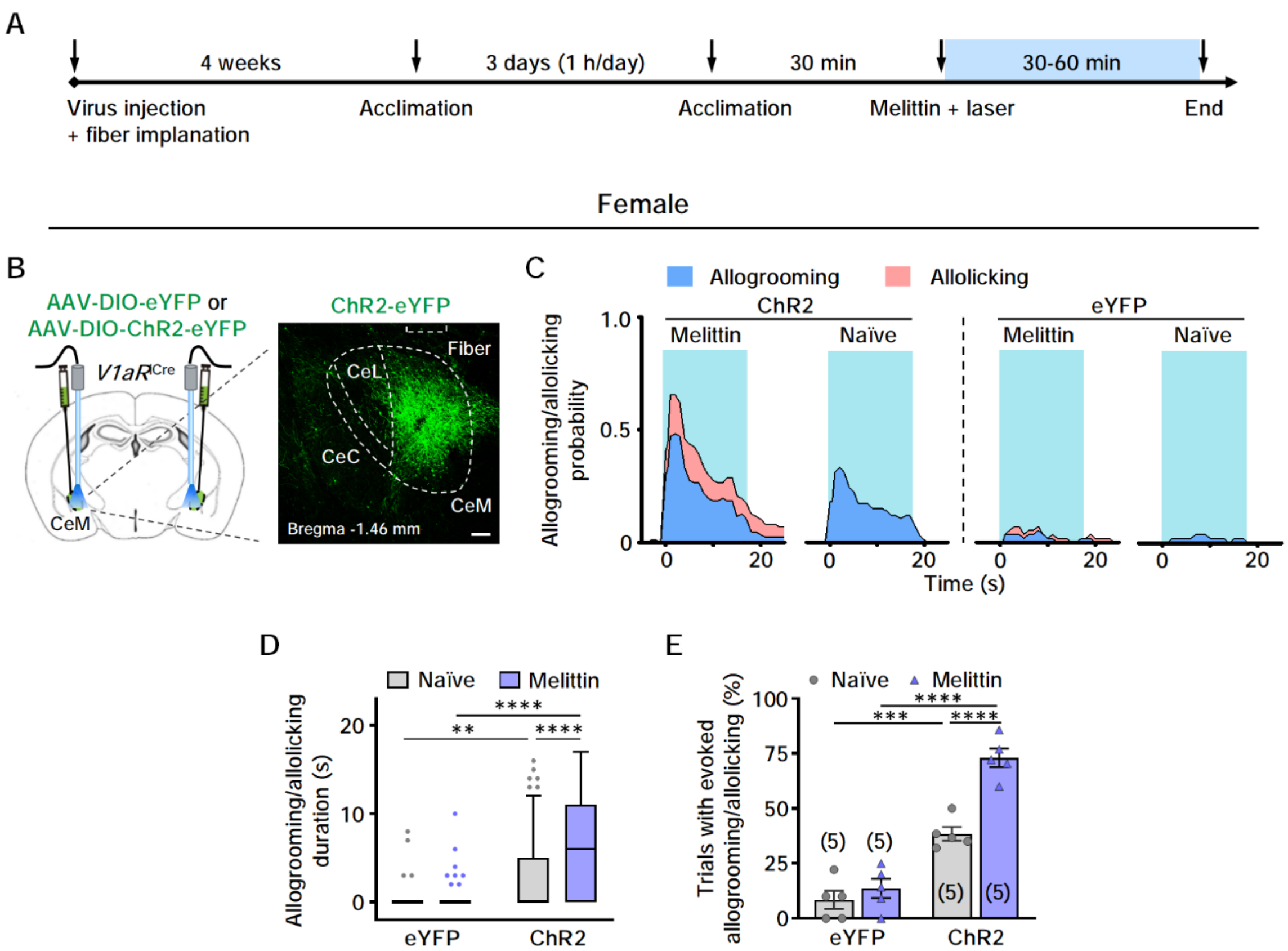

Supplemental Figure 9. Opto-activation of CeM^{V1aR} neurons drives caregiving-like behavior in females. (**A**) Timeline of optogenetic experiments. (**B**) Schematic of CeM injections and fiber implantations, and a representative image showing ChR2-eYFP expression. (**C** and **D**) Probability (C) and duration (D) of allogrooming/allolicking toward naïve or melittin-treated cagemates during opto-activation (17 s) in eYFP vs. ChR2 mice. (**E**) Percentage of trials in each eYFP and ChR2 mice with evoked allogrooming/allolicking during opto-activation. Scale bar, 100 µm. n = 50-93 trials from 5 females in B-E. Statistical results are included in Table S3. Mean ± SEM in E. Box and whiskers (center line: median; box limits: quartiles; whiskers: 1.5x interquartile range; points: outliers) in D.

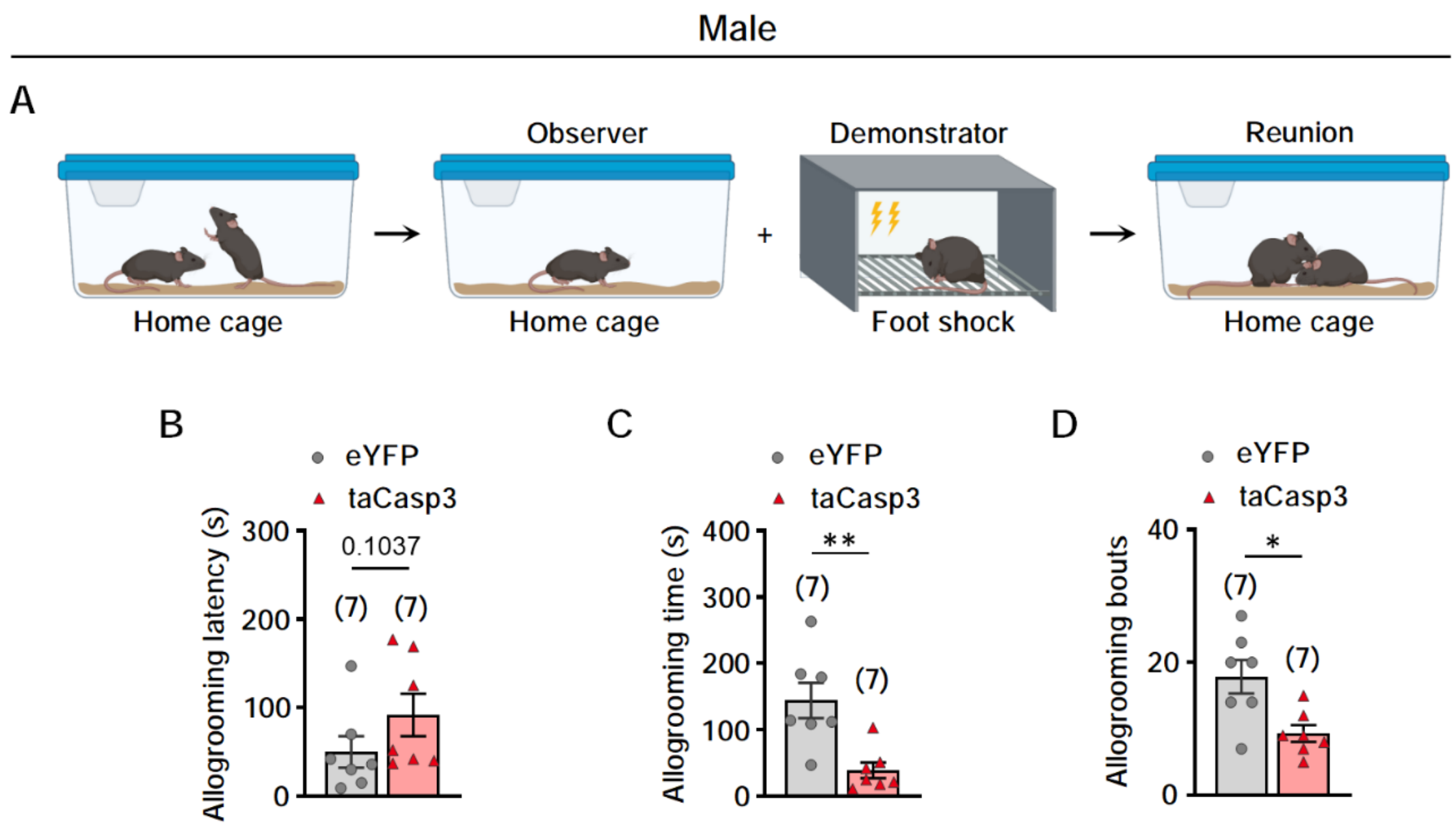

Supplemental Figure 10. Foot-shock induced allogrooming in male mice with CeM^{V1aR} neuronal ablation. (**A**) Schematic of the prosocial interaction assay. (**B-D**) Latency (B), duration (C) and bouts (D) of allogrooming towards foot-shocked cagemates in eYFP and taCasp3 mice. n = 7 mice in B-D. Statistical results are included in Table S3. Data are mean ± SEM.

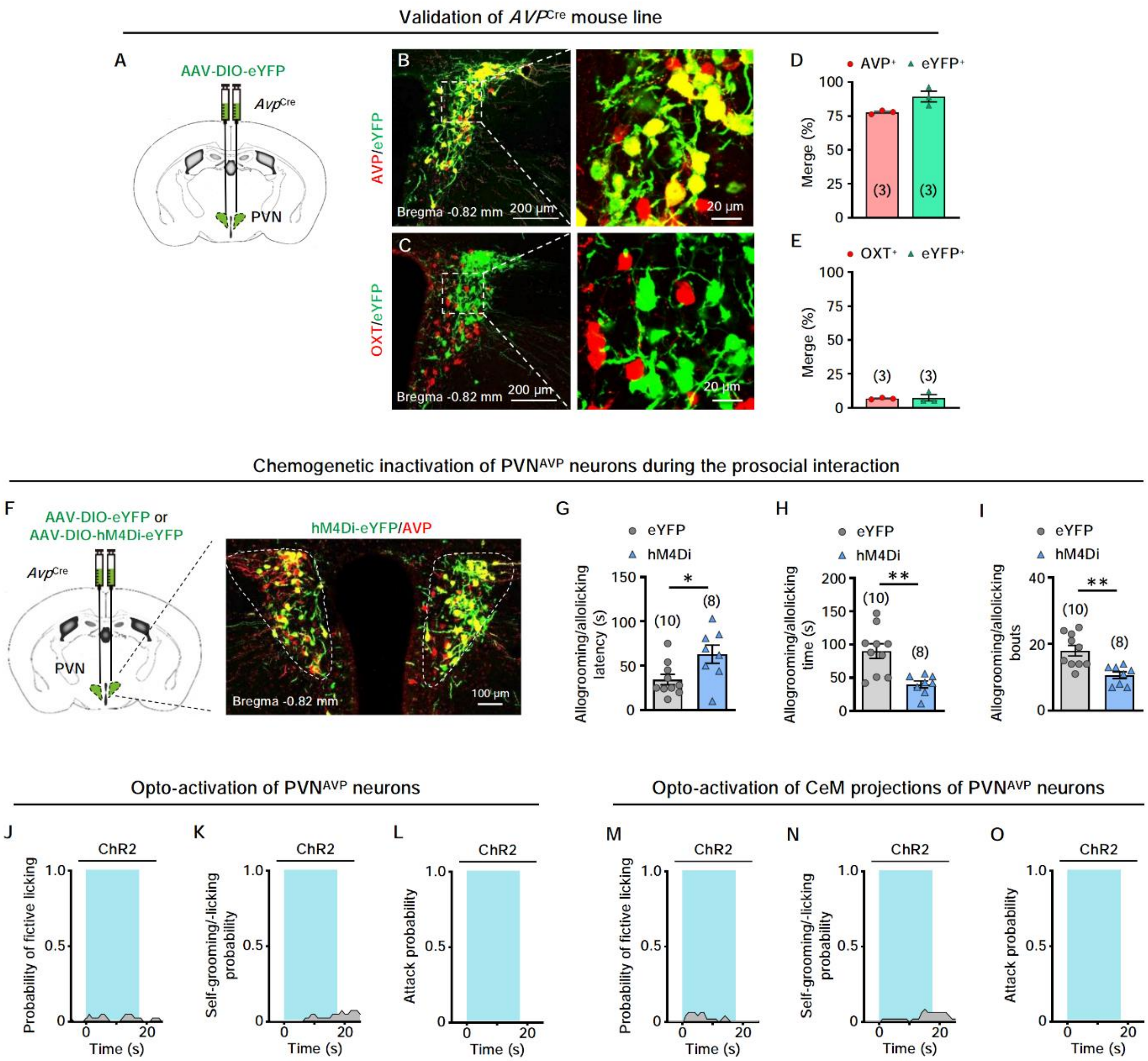

Supplemental Figure 11. Behavioral phenotypes of PVN^{AVP} neuronal manipulations in males. (**A**) Schematic of PVN injections. (**B**-**E**) Co-localizations and quantifications of eYFP^{+}/AVP^{+} (B, C) and eYFP^{+}/OXT^{+} (D, E) double-labeled cells in the PVN of *AVP*^{Cre} mice after virus injections. (**F**) Schematic of PVN injections and a representative image showing hM4Di-eYFP/AVP co-expression in the PVN. (**G**-**I**) Latency (G), duration (H) and bouts (I) of allogrooming/allolicking toward pain-suffering cagemates after clozapine injection in eYFP and hM4Di observers. (**J**-**L**) Behaviors induced by opto-activation (17 s) of PVN^{AVP} somas in non-social context (J, K) and

social context with melittin-treated cagemates (L). (**M-O**) Behaviors induced by opto-activation (17 s) of CeM projecting axonal terminals of PVN^{AVP} neurons in non-social context (M, N) and social context with melittin-treated cagemates (O). Scale bars, 20 μm (magnified, B and C), 100 μm (F), 200 μm (original, B and C). n = 40-90 trials from 4-5 mice for J-L. n = 46-80 trials from 4-5 mice for M-O. Statistical results are included in Table S3. Data are mean ± SEM.

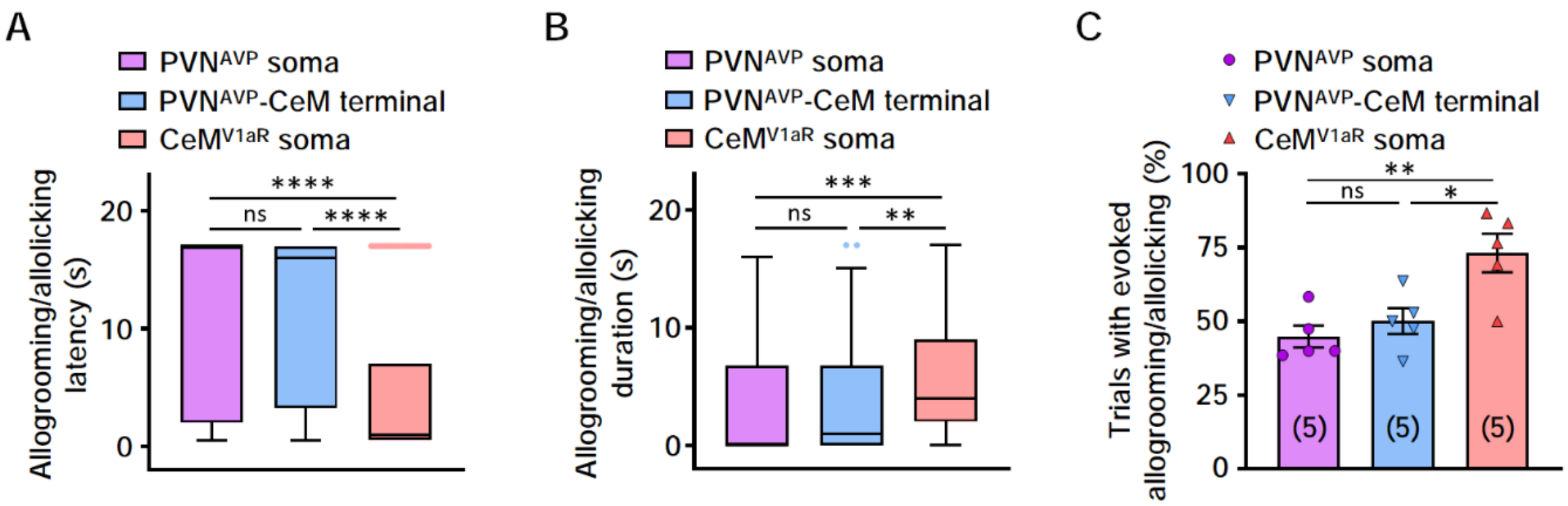

Supplemental Figure 12. Comparisons of evoked caregiving-like behavior by opto-activations of different components in PVN^{AVP}-CeM^{V1aR} circuitry. (**A** and **B**) Latency (A) and duration (B) of evoked allogrooming/allolicking toward melittin-treated cagemates during opto-activation (17 s) of PVN^{AVP} somas, PVN^{AVP}-CeM terminals and CeM^{V1aR} somas, respectively. (**C**) Percentage of trials in each ChR2 mice with evoked allogrooming/allolicking toward melittin-treated cagemates during opto-activation (17 s) of PVN^{AVP} somas, PVN^{AVP}-CeM terminals and CeM^{V1aR} somas, respectively. n = 80-93 trials from 5 mice for A-C. Statistical results are included in Table S3. Mean ± SEM in C. Box and whiskers (center line: median; box limits: quartiles; whiskers: 1.5x interquartile range; points: outliers) in A and B.

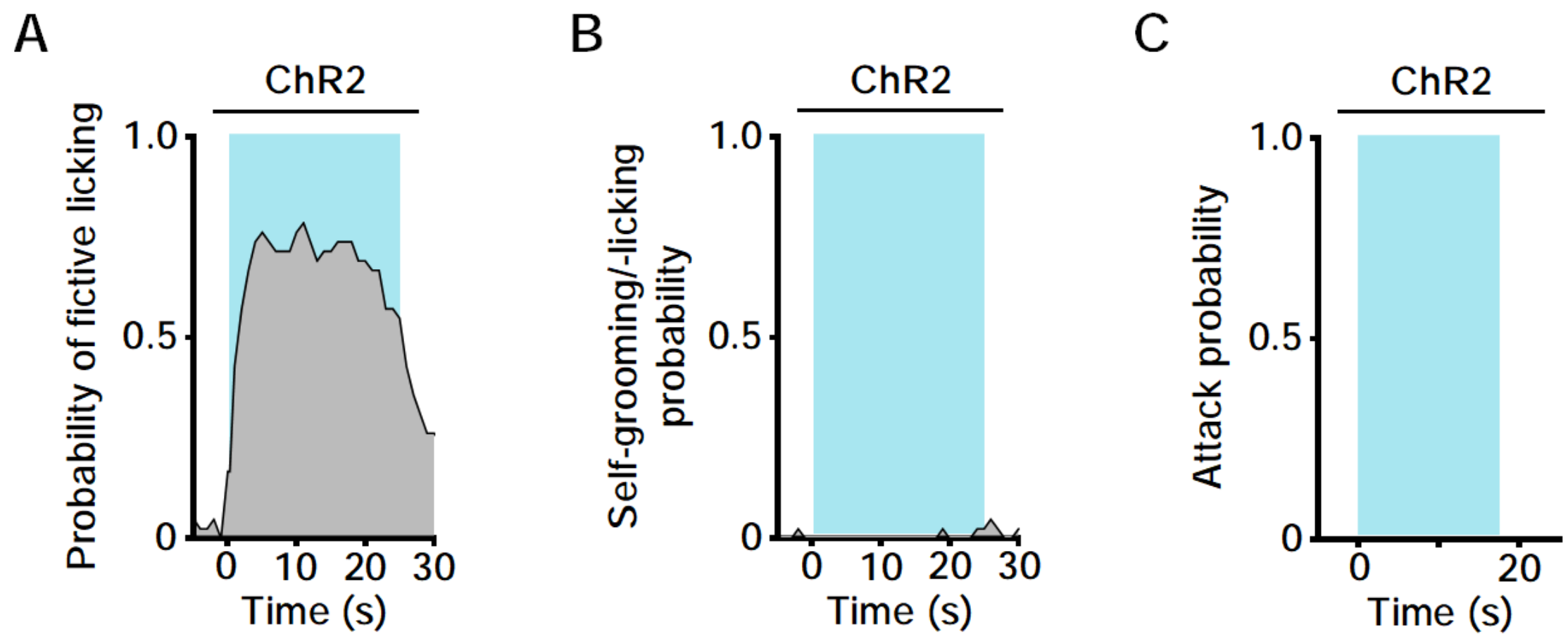

Supplemental Figure 13. Behavioral phenotypes by opto-activation of VTA projecting axonal terminals of CeM^{V1aR} neurons. (**A**-**C**) Behaviors induced by opto-activation (25 s in A and B, 17 s in C) of ChR2 mice in non-social context (A, B) and social context with melittin-treated cagemates (C). n = 42-113 trials from 4-5 mice for A-C.

Supplemental Video 1

An observer mouse grooms the body and licks the injured nape region of a melittin-treated cagemate.

Supplemental Video 2

Calcium dynamics in CeM^{V1aR} neurons of an observer mouse during social investigation toward a melittin-treated cagemate.

Supplemental Video 3

Calcium dynamics in CeM^{V1aR} neurons of an observer mouse during spontaneous allogrooming toward a melittin-treated cagemate.

Supplemental Video 4

Calcium dynamics in CeM^{V1aR} neurons of an observer mouse during spontaneous allolicking toward a melittin-treated cagemate.

Supplemental Video 5

Opto-activation of CeM^{V1aR} neurons in an observer mouse elicits robust, time-locked allogrooming/allolicking toward a melittin-treated cagemate.

Supplemental Video 6

Opto-inhibition of CeM^{V1aR} neurons in an observer mouse timely suppresses spontaneous allolicking toward injured nape of a melittin-treated cagemate.

Supplemental Video 7

Opto-activation of PVN^{AVP}-CeM projections in an observer mouse initiates robust, time-locked allolicking toward a melittin-treated cagemate.

Table S1. Rank of the normalized fold change in number of c-Fos+ neurons across brain regions in the observers interacting with cagemates in pain vs. naïve ones.

Brain region	Naïve (mean ± SEM)	Melittin (mean ± SEM)	Fold change	*p* value (two tailed)
CeA	253.33 ± 7.31	449.33 ± 4.37	1.7685	< 0.0001
VTA	67.67 ± 4.67	116.0 ± 11.27	1.7143	0.0166
PVN	48.67 ± 3.76	69.33 ± 4.67	1.421	0.0261
IC	434.7 ± 32.75	556.3 ± 91.24	1.279	0.2778
PrL	322.3 ± 14.11	406.0 ± 24.52	1.258	0.0416
NAc	224.3 ± 7.17	270.3 ± 21.4	1.201	0.1112
BNST	147.8 ± 6.89	176.5 ± 10.4	1.194	0.0830
ACC	343.0 ± 11.79	401.0 ± 32.51	1.174	0.1688
Hippo	1300.0 ± 128.6	1385.0 ± 62.89	1.079	0.5856
PAG	874.3 ± 87.83	762.7 ± 117.6	0.864	0.4892

Abbreviation: ACC, anterior cingulate cortex; BNST, bed nucleus of the stria terminalis; CeA, central amygdala; Hippo, hippocampus; IC, insular cortex; NAc, nucleus accumbens; PAG, periaqueductal gray; PrL, prelimbic cortex; PVN, hypothalamic paraventricular nucleus; VTA, ventral tegmental area.

Table S2. Statistical methods, and *P* values shown in Figures 1-8.

Figure	Sample size	Normality test	Equal variance test	Statistic method	*p* value (two tailed)	Post hoc multiple comparisons test
Figure 1C (1st 30 min)	n = 8 and 9 male mice in each group	passed	failed	Brown-Forsythe and Welch ANOVA tests with Dunnett's T3 multiple comparisons	$p < 0.0001$	Naive-1st vs. Saline-1st: $p = 0.0692$; Naive-1st vs. Melittin-1st: $p < 0.0001$; Saline-1st vs. Melittin-1st: $p = 0.0022$
Figure 1C (2nd 30 min)	n = 8 and 9 male mice in each group	failed		Kruskal-Wallis test with Dunn's multiple comparisons	$p = 0.0709$	Naive-2nd vs. Saline-2nd: $p = 0.2040$; Naive-2nd vs. Melittin-2nd: $p = 0.0243$; Saline-2nd vs. Melittin-2nd: $p > 0.9999$
Figure 1C (Melittin, 1st vs 2nd)	n = 9 male mice in melittin group	passed	passed	Paired *t* test	$p = 0.0004$	
Figure 1E	n = 9 male mice in each group	passed	passed	Unpaired *t* test	$p = 0.0003$	
Figure 1G	n = 4 and 5 male mice in each group	passed		Two-way ANOVA with Tukey's multiple comparisons		No divider/Naïve vs Divider/Naïve: $p = 0.9961$; No divider/Naïve vs No divider/Melittin: $p = 0.2581$; No divider/Naïve vs Divider/Melittin: $p < 0.0001$; Divider/Naïve vs No divider/Melittin: $p = 0.3531$; Divider/Naïve vs Divider/Melittin: $p < 0.0001$; No divider/Melittin vs Divider/Melittin: $p < 0.0001$;
Figure 1H	n = 9 and 10 male mice in each group	passed	failed	Unpaired *t* test with Welch's correction	$p = 0.8921$	
Figure 1I	n = 9 male mice in	passed	failed	Unpaired *t* test with	$p = 0.0396$	

	each group			Welch's correction		
Figure 1L	n = 8 male mice in each group	failed		Mann Whitney *U* test	$p = 0.8211$	
Figure 1M	n = 16 male mice	passed		Linear regression (Pearson correlation)	$p = 0.0210$	r = -0.5704
Figure.1N	n = 16 male mice	passed		Linear regression (Pearson correlation)	$p = 0.6598$	r = -0.1193
Figure 1O	n = 16 male mice	failed		Linear regression (Pearson correlation)	$p = 0.6813$	r = 0.1114
				Linear regression (Spearman correlation)	$p = 0.4908$	r = 0.1845
Figure 1P	n = 16 male mice	passed		Linear regression (Pearson correlation)	$p = 0.7885$	r = 0.07289
Figure 1Q	n = 16 male mice	failed		Linear regression (Pearson correlation)	$p = 0.0079$	r = -0.6376
				Linear regression (Spearman correlation)	$p = 0.0032$	r = -0.7029
Figure 1R	n = 16 male mice	failed		Linear regression (Pearson correlation)	$p = 0.0003$	r = 0.7872
				Linear regression (Spearman correlation)	$p = 0.0263$	r = 0.5592
Figure 2B	n = 3 male mice in each group	passed	passed	One-way ANOVA with Tukey test multiple comparisons	$p < 0.0001$	WT vs HET: $p < 0.0001$; WT vs MUT: $p < 0.0001$; HET vs MUT: $p < 0.0001$
Figure 2C	n = 3 male mice in each group	passed	passed	One-way ANOVA with Tukey test multiple comparisons	$p < 0.0001$	WT vs HET: $p < 0.0001$; WT vs MUT: $p < 0.0001$; HET vs MUT: $p = 0.0003$
Figure 2D	n = 8 male mice in each group	failed		Mann Whitney *U* test	$p = 0.0196$	
Figure 2E	n = 8 male mice in each group	passed	failed	Unpaired *t* test with Welch's correction	$p = 0.0083$	
Figure 2F	n = 8 male mice in	failed		Mann Whitney *U* test	$p = 0.0011$	

	each group					
Figure 2G	n = 4 and 5 male mice in each group	passed		Two-way ANOVA with Tukey's multiple comparisons		WT/Naïve vs MUT/Naïve: $p = 0.9979$; WT/Naïve vs WT/Melittin: $p = 0.0059$; WT/Naïve vs MUT/Melittin: $p = 0.9681$; MUT/Naïve vs WT/Melittin: $p = 0.0042$; MUT/Naïve vs MUT/Melittin: $p = 0.9209$; WT/Melittin vs MUT/Melittin: $p = 0.0142$
Figure 2I	n = 8 male mice in each group	passed		Paired *t* test	WT: $p = 0.0094$; MUT: $p = 0.0057$	
Figure 2J	n = 8 male mice in each group	passed	passed	Unpaired *t* test	$p = 0.0001$	
Figure 2N	n = 3 male mice in each group	passed	passed	Unpaired *t* test	$p < 0.0001$	
Figure 2P	n = 3 male mice in each group	passed	passed	Unpaired *t* test	$p = 0.0023$	
Figure 2Q	n = 8 male mice in each group	passed	failed	Unpaired *t* test with Welch's correction	$p = 0.0271$	
Figure 2R	n = 8 male mice in each group	passed	failed	Unpaired *t* test with Welch's correction	$p = 0.0140$	
Figure 2S	n = 8 male mice in each group	passed	passed	Unpaired *t* test	$p = 0.0020$	
Figure 2T	n = 5 male mice in each group	passed	passed	Unpaired *t* test	$p = 0.0026$	
Figure 2V	n = 7 male mice in each group	passed		Paired *t* test	Scramble: $p = 0.0033$; siRNA: $p = 0.6743$	
Figure 2W	n = 7 male mice in each group	passed	passed	Unpaired *t* test	$p = 0.0375$	
Figure 3J	n = 12 and 17 trials from 7 male mice in each group	failed		Mann Whitney *U* test	$p < 0.0001$	
Figure 3M	n = 25 trials from 5	failed		Wilcoxon	$p < 0.0001$	

	male mice			matched-pairs test		
Figure 3P	n = 11 trials from 5 male mice	passed		Paired *t* test	$p < 0.0001$	
Figure 3Q	n = 11 and 25 trials from 5 male mice	failed		Mann Whitney *U* test	$p = 0.4558$	
Figure 3T	n = 16 trials from 5 male mice	failed		Wilcoxon matched-pairs test	$p = 0.0008$	
Figure 4B	n = 3 male mice in each group	passed	passed	Unpaired *t* test	$p < 0.0001$	
Figure 4D	n = 8 and 9 male mice in each group	passed	passed	Unpaired *t* test	$p = 0.0071$	
Figure 4E	n = 8 and 9 male mice in each group	failed		Mann Whitney *U* test	$p = 0.0016$	
Figure 4F	n = 8 and 9 male mice in each group	passed	passed	Unpaired *t* test	$p = 0.0150$	
Figure 4H	n = 8 and 9 male mice in each group	passed		Paired *t* test	eYFP: $p = 0.0112$; taCasp3: $p = 0.1308$	
Figure 4I	n = 8 and 9 male mice in each group	passed	passed	Unpaired *t* test	$p = 0.0027$	

Figure 4L	n = 52 to 98 trials from 4 to 5 male mice in each group	failed		Two-way ANOVA with Tukey's multiple comparisons		eYFP/Naïve vs. eYFP/Melittin: $p = 0.9203$; eYFP/Naïve vs. ChR2/Naïve: $p = 0.0027$; eYFP/Naïve vs. ChR2/Melittin: $p < 0.0001$; eYFP/Melittin vs. ChR2/Naïve: $p = 0.0147$; eYFP/Melittin vs. ChR2/Melittin: $p < 0.0001$; ChR2/Naïve vs. ChR2/Melittin: $p < 0.0001$
				Kruskal-Wallis test with Dunn's multiple comparisons	$p < 0.0001$	eYFP/Naïve vs. eYFP/Melittin: $p > 0.9999$; eYFP/Naïve vs. ChR2/Naïve: $p = 0.0014$; eYFP/Naïve vs. ChR2/Melittin: $p < 0.0001$; eYFP/Melittin vs. ChR2/Naïve: $p = 0.0320$; eYFP/Melittin vs. ChR2/Melittin: $p < 0.0001$; ChR2/Naïve vs. ChR2/Melittin: $p < 0.0001$

Figure 4M	n = 4 and 5 male mice in each group	passed		Two-way ANOVA with Tukey's multiple comparisons		eYFP/Naïve vs. eYFP/Melittin: $p = 0.7756$;<br>eYFP/Naïve vs. ChR2/Naïve: $p = 0.0063$;<br>eYFP/Naïve vs. ChR2/Melittin: $p < 0.0001$;<br>eYFP/Melittin vs. ChR2/Naïve: $p = 0.0420$;<br>eYFP/Melittin vs. ChR2/Melittin: $p < 0.0001$;<br>ChR2/Naïve vs. ChR2/Melittin: $p = 0.0004$
Figure 4R	n = 52 and 54 trials from 4 to 5 male mice in each group	failed		Mann Whitney *U* test	$p < 0.0001$	
Figure 5H	n = 3 and 4 male mice in each group	passed	passed	Unpaired *t* test	$p < 0.0001$	
Figure 5J	n = 7 male mice in each group	passed	passed	Unpaired *t* test	$p = 0.0004$	
Figure 5K	n = 7 male mice in each group	passed	failed	Unpaired *t* test with Welch's correction	$p = 0.0079$	
Figure 5L	n = 7 male mice in each group	passed	passed	Unpaired *t* test	$p = 0.0046$	
Figure 5M	n = 6 male mice in each group	passed	passed	Unpaired *t* test	$p = 0.0174$	
Figure 5O	n = 7 male mice in each group	eYFP: failed; taCasp3: passed		eYFP: Wilcoxon matched-pairs test; taCasp3: Paired *t* test	eYFP: $p = 0.0156$; taCasp3: $p = 0.7559$	

Figure 5P	n = 7 male mice in each group	failed		Mann Whitney *U* test	$p = 0.0175$	
Figure 5S	n = 38 to 92 trials from 4 to 5 male mice in each group	failed		Two-way ANOVA with Tukey's multiple comparisons		eYFP/Naïve vs. eYFP/Melittin:<br>$p = 0.9259$;<br>eYFP/Naïve vs. ChR2/Naïve:<br>$p = 0.9108$;<br>eYFP/Naïve vs. ChR2/Melittin:<br>$p < 0.0001$;<br>eYFP/Melittin vs. ChR2/Naïve:<br>$p > 0.9999$;<br>eYFP/Melittin vs. ChR2/Melittin:<br>$p = 0.0013$;<br>ChR2/Naïve vs. ChR2/Melittin:<br>$p = 0.0005$
				Kruskal-Wallis test with Dunn's multiple comparisons	$p < 0.0001$	eYFP/Naïve vs. eYFP/Melittin:<br>$p > 0.9999$;<br>eYFP/Naïve vs. ChR2/Naïve:<br>$p > 0.9999$;<br>eYFP/Naïve vs. ChR2/Melittin:<br>$p = 0.0003$;<br>eYFP/Melittin vs. ChR2/Naïve:<br>$p > 0.9999$;<br>eYFP/Melittin vs. ChR2/Melittin:<br>$p = 0.0095$;<br>ChR2/Naïve vs. ChR2/Melittin:<br>$p = 0.0397$
Figure 5T	n = 4 and 5 male mice in each group	passed		Two-way ANOVA with Tukey's multiple comparisons		eYFP/Naïve vs. eYFP/Melittin:<br>$p = 0.7400$;<br>eYFP/Naïve vs. ChR2/Naïve:<br>$p = 0.2000$;<br>eYFP/Naïve vs. ChR2/Melittin:<br>$p = 0.0005$;

						eYFP/Melittin vs. ChR2/Naïve:<br>$p = 0.7378$;<br>eYFP/Melittin vs. ChR2/Melittin:<br>$p = 0.0034$;<br>ChR2/Naïve vs. ChR2/Melittin:<br>$p = 0.0164$
Figure 5W	n = 37 to 80 trials from 4 to 5 male mice in each group	failed		Two-way ANOVA with Tukey's multiple comparisons		eYFP/Naïve vs. eYFP/Melittin:<br>$p = 0.9898$;<br>eYFP/Naïve vs. ChR2/Naïve:<br>$p = 0.2609$;<br>eYFP/Naïve vs. ChR2/Melittin:<br>$p < 0.0001$;<br>eYFP/Melittin vs. ChR2/Naïve:<br>$p = 0.2873$;<br>eYFP/Melittin vs. ChR2/Melittin:<br>$p < 0.0001$;<br>ChR2/Naïve vs. ChR2/Melittin:<br>$p = 0.0028$
				Kruskal-Wallis test with Dunn's multiple comparisons	$p < 0.0001$	eYFP/Naïve vs. eYFP/Melittin:<br>$p > 0.9999$;<br>eYFP/Naïve vs. ChR2/Naïve:<br>$p = 0.1967$;<br>eYFP/Naïve vs. ChR2/Melittin:<br>$p < 0.0001$;<br>eYFP/Melittin vs. ChR2/Naïve:<br>$p = 0.2263$;<br>eYFP/Melittin vs. ChR2/Melittin:<br>$p < 0.0001$;<br>ChR2/Naïve vs. ChR2/Melittin:<br>$p = 0.0394$
Figure 5X	n = 4 and 5 male mice in each group	passed		Two-way ANOVA with Tukey's multiple comparisons		eYFP/Naïve vs. eYFP/Melittin:<br>$p = 0.9654$;<br>eYFP/Naïve vs. ChR2/Naïve:

						$p = 0.0634$;<br>eYFP/Naïve vs. ChR2/Melittin:<br>$p = 0.0002$;<br>eYFP/Melittin vs. ChR2/Naïve:<br>$p = 0.1142$;<br>eYFP/Melittin vs. ChR2/Melittin:<br>$p = 0.0003$;<br>ChR2/Naïve vs. ChR2/Melittin:<br>$p = 0.0334$
Figure 6G (AVP)	n = 26 recorded cells from 5 male and 3 female mice	failed		Wilcoxon matched-pairs test	$p < 0.0001$	
Figure 6G (OXT)	n = 26 recorded cells from 4 male and 3 female mice	passed		Paired *t* test	$p = 0.3613$	
Figure 6G (Manning+AVP)	n = 20 recorded cells from 2 male and 3 female mice	passed		Paired *t* test	$p = 0.4502$	
Figure 6G (L368899+AVP)	n = 17 recorded cells from 3 male and 1 female mice	failed		Wilcoxon matched-pairs test	$p < 0.0001$	
Figure 6H (AVP)	n = 26 recorded cells from 5 male and 3 female mice	failed		Wilcoxon matched-pairs test	$p < 0.0001$	
Figure 6H (OXT)	n = 26 recorded cells from 4 male and 3 female mice	failed		Wilcoxon matched-pairs test	$p = 0.5781$	
Figure 6H (Manning+AVP)	n = 20 recorded cells from 2 male and 3 female mice	failed		Paired *t* test	$p = 0.1644$	
				Wilcoxon matched-pairs test	$p = 0.1016$	
Figure 6H (L368899+AVP)	n = 17 recorded cells from 3 male and 1 female mice	failed		Wilcoxon matched-pairs test	$p < 0.0001$	

Figure 6L	n = 7 male mice	passed		Paired *t* test	$p = 0.0519$	
Figure 6M	n = 7 male mice	failed		Wilcoxon matched-pairs test	$p = 0.0156$	
Figure 6N	n = 7 male mice	passed		Paired *t* test	$p = 0.0038$	
Figure 6O	n = 6 male mice	passed		Paired *t* test	$p = 0.1103$	
Figure 6P	n = 6 male mice	passed		Paired *t* test	$p = 0.7667$	
Figure 6Q	n = 6 male mice	passed		Paired *t* test	$p = 0.2423$	
Figure 7E	n = 56 recorded cells from 6 male and 3 female mice	failed		Wilcoxon matched-pairs test	$p < 0.0001$	
Figure 7F	n = 18 recorded cells showing firing increase from 3 male and 3 female mice	failed		Wilcoxon matched-pairs test	$p < 0.0001$	
Figure 7H	n = 33 recorded cells from 1 male and 2 female mice	failed		Wilcoxon matched-pairs test	$p = 0.4038$	
Figure 7I	n = 33 recorded cells from 1 male and 2 female mice	failed		Wilcoxon matched-pairs test	$p = 0.0801$	
Figure 7K	n = 47 recorded cells from 3 male and 4 female mice	failed		Wilcoxon matched-pairs test	$p < 0.0001$	
Figure 7L	n = 17 recorded cells showing firing increase from 3 male and 4 female mice	failed		Wilcoxon matched-pairs test	$p < 0.0001$	
Figure 8D	n = 7 male mice in each group	passed	failed	Unpaired *t* test with Welch's correction	$p = 0.0175$	
Figure 8E	n = 7 male mice in each group	passed	passed	Unpaired *t* test	$p = 0.0149$	

Figure 8F	n = 7 male mice in each group	passed	passed	Unpaired *t* test	$p = 0.0034$	
Figure 8I	n = 48 to 114 trials from 4 to 5 male mice in each group	failed		Two-way ANOVA with Tukey's multiple comparisons		eYFP/Naïve vs. eYFP/Melittin: $p = 0.9479$;<br>eYFP/Naïve vs. ChR2/Naïve: $p = 0.0046$;<br>eYFP/Naïve vs. ChR2/Melittin: $p < 0.0001$;<br>eYFP/Melittin vs. ChR2/Naïve: $p = 0.0290$;<br>eYFP/Melittin vs. ChR2/Melittin: $p < 0.0001$;<br>ChR2/Naïve vs. ChR2/Melittin: $p < 0.0001$
				Kruskal-Wallis test with Dunn's multiple comparisons	$p < 0.0001$	eYFP/Naïve vs. eYFP/Melittin: $p > 0.9999$;<br>eYFP/Naïve vs. ChR2/Naïve: $p = 0.0007$;<br>eYFP/Naïve vs. ChR2/Melittin: $p < 0.0001$;<br>eYFP/Melittin vs. ChR2/Naïve: $p = 0.0254$;<br>eYFP/Melittin vs. ChR2/Melittin: $p < 0.0001$;<br>ChR2/Naïve vs. ChR2/Melittin: $p = 0.0019$

Figure 8J	n = 4 to 5 male mice in each group	passed		Two-way ANOVA with Tukey's multiple comparisons		eYFP/Naïve vs. eYFP/Melittin: *p* = 0.5487; eYFP/Naïve vs. ChR2/Naïve: *p* = 0.0158; eYFP/Naïve vs. ChR2/Melittin: *p* < 0.0001; eYFP/Melittin vs. ChR2/Naïve: *p* = 0.2008: eYFP/Melittin vs. ChR2/Melittin: *p* = 0.0009; ChR2/Naïve vs. ChR2/Melittin: *p* = 0.0311
Figure 8M	n = 104 and 65 trials from 5 male mice in each group	failed		Wilcoxon matched-pairs test	*p* = 0.8041	
Figure 8N	n = 5 male mice	passed		Paired *t* test	*p* = 0.9404	
Figure 8O	n = 5 male mice	passed		Paired *t* test	*p* = 0.0471	
Figure 8R	n = 6 male mice in each group	passed	passed	Unpaired *t* test	5 Hz: *p* = 0.3611; 10 Hz: *p* = 0.0289; 20 Hz: *p* = 0.0028	
Figure 8T	n = 6 and 7 male mice in each group	passed	passed	**Unpaired *t* test**: (a) eYFP/Melittin/Laser vs ChR2/Melittin/Laser; (b) eYFP/Naïve/No laser vs ChR2/Naïve/No laser. **Paired *t* test**: (c) ChR2/Melittin/Laser vs ChR2/Naïve/No laser	(a): *p* = 0.0002; (b): *p* = 0.5523; (c): *p* = 0.3891	

Table S3. Statistical methods, and *P* values shown in Supplemental Figures 1-12.

Figure	Sample size	Normality test	Equal variance test	Statistic method	*p* value (two tailed)	Post hoc multiple comparisons test
Supplemental Figure 1C	n = 10 male mice	passed		Paired *t* test	$p = 0.0030$	
Supplemental Figure 2A	n = 16 male mice	failed		Linear regression (Pearson correlation)	$p = 0.2906$;	r = -0.2817
				Linear regression (Spearman correlation)	$p = 0.2306$;	r = -0.3166
Supplemental Figure 2B	n = 16 male mice	failed		Linear regression (Pearson correlation)	$p = 0.4767$;	r = -0.1918
				Linear regression (Spearman correlation)	$p = 0.5772$;	r = -0.1504
Supplemental Figure 2C	n = 16 male mice	failed		Linear regression (Pearson correlation)	$p = 0.4142$;	r = -0.2194
				Linear regression (Spearman correlation)	$p = 0.9869$;	r = -0.005882
Supplemental Figure 3B	n = 8 and 7 male mice in each group	passed	passed	Unpaired *t* test	$p = 0.5960$	
Supplemental Figure 3C	n = 7 male mice in each group	passed	passed	Unpaired *t* test	$p = 0.4588$	
Supplemental Figure 3D	n = 8 male mice in each group	failed		Mann Whitney *U* test	$p = 0.9591$	
Supplemental Figure 3E	n = 8 male mice in each group	failed		Mann Whitney *U* test	Left: $p > 0.9999$; Right: $p > 0.9999$	
Supplemental Figure 3F	n = 8 male mice in each group	passed	passed	Unpaired *t* test	Left: $p = 0.4599$; Right: $p = 0.4813$	
Supplemental Figure 3H	n = 8 male mice in each group	passed	passed	Unpaired *t* test	WT: $p < 0.0001$ MUT: $p < 0.0001$	
Supplemental Figure 3I	n = 8 male mice in each group	passed	passed	Unpaired *t* test	$p = 0.2648$	

Supplemental Figure 3K	n = 8 male mice in each group	passed	passed	Unpaired *t* test	WT: $p = 0.0002$<br>MUT: $p = 0.0003$	
Supplemental Figure 3L	n = 8 male mice in each group	passed	passed	Unpaired *t* test	$p = 0.8248$	
Supplemental Figure 3N	n = 3 male mice in each group	passed		PVN: Unpaired *t* test with Welch's correction;<br>SON: Unpaired *t* test	PVN: $p = 0.0252$;<br>SON: $p = 0.0010$	
Supplemental Figure 3O	n = 8 and 9 male mice in each group	passed	passed	Unpaired *t* test	$p = 0.6819$	
Supplemental Figure 3P	n = 8 and 9 male mice in each group	passed	passed	Unpaired *t* test	$p = 0.5441$	
Supplemental Figure 3Q	n = 8 and 9 male mice in each group	failed		Mann Whitney *U* test	$p = 0.1900$	
Supplemental Figure 3S	n = 8 and 7 male mice in each group	passed	passed	Unpaired *t* test	WT: $p = 0.0006$<br>KO: $p = 0.0106$	
Supplemental Figure 3T	n = 8 and 7 male mice in each group	passed	passed	Unpaired *t* test	$p = 0.9458$	
Supplemental Figure 6B	n = 3 male mice in each group	passed	passed	Unpaired *t* test	$p = 0.0006$	
Supplemental Figure 6C	n = 6 male mice in each group	passed	passed	Unpaired *t* test	$p > 0.9999$	
Supplemental Figure 6D	n = 6 male mice in each group	passed	passed	Unpaired *t* test	$p = 0.9336$	
Supplemental Figure 6E	n = 6 male mice in each group	failed		Mann Whitney *U* test	$p = 0.5281$	
Supplemental Figure 6G	n = 3 male mice in each group	passed	passed	Unpaired *t* test	$p = 0.0090$	
Supplemental Figure 6H	n = 6 and 5 male mice in each group	passed	passed	Unpaired *t* test	$p = 0.9379$	
Supplemental Figure 6I	n = 6 and 5 male mice in each group	passed	passed	Unpaired *t* test	$p = 0.7764$	

Supplemental Figure 6J	n = 6 and 5 male mice in each group	passed	passed	Unpaired *t* test	$p = 0.7534$	
Supplemental Figure 7C	n = 11 and 9 trials from 4 female mice in each group	passed	failed	Unpaired *t* test with Welch's correction	$p = 0.0032$	
Supplemental Figure 7F	n = 10 trials from 3 female mice	passed		Paired *t* test	$p < 0.0001$	
Supplemental Figure 7I	n = 11 trials from 3 female mice	failed		Wilcoxon matched-pairs test	$p = 0.001$	
Supplemental Figure 7J	n = 10 and 11 trials from 3 female mice in each group	failed		Mann Whitney *U* test	$p = 0.1971$	
Supplemental Figure 7M	n = 13 trials from 3 female mice	passed		Paired *t* test	$p = 0.0284$	
Supplemental Figure 8A	n = 9 and 8 male mice in each group	passed	passed	Unpaired *t* test	$p = 0.3931$	
Supplemental Figure 8B	n = 9 and 8 male mice in each group	failed		Mann Whitney *U* test	$p = 0.8884$	
Supplemental Figure 8C	n = 9 and 8 male mice in each group	failed		Mann Whitney *U* test	$p = 0.3839$	
Supplemental Figure 8E	n = 7 and 6 male mice in each group	passed	passed	Unpaired *t* test	eYFP: $p = 0.0044$<br>taCasp3: $p = 0.0300$	
Supplemental Figure 8F	n = 7 and 6 male mice in each group	passed	passed	Unpaired *t* test	$p = 0.3695$	
Supplemental Figure 8H	n = 7 and 6 male mice in each group	passed	passed	Unpaired *t* test	eYFP: $p = 0.0206$<br>taCasp3: $p = 0.0121$	
Supplemental Figure 8I	n = 7 and 6 male mice in each group	passed	passed	Unpaired *t* test	$p = 0.7428$	
Supplemental Figure 8K	n = 9 and 10 male mice in each group	failed		Mann Whitney *U* test	$p = 0.0027$	

Supplemental Figure 8L	n = 9 and 10 male mice in each group	passed	failed	Unpaired *t* test with Welch's correction	$p = 0.0035$	
Supplemental Figure 8M	n = 9 and 10 male mice in each group	passed	passed	Unpaired *t* test	$p = 0.0011$	
Supplemental Figure 9D	n = 50 to 93 trials from 5 female mice in each group	failed		Two-way ANOVA with Tukey's multiple comparisons		eYFP/Naïve vs. eYFP/Melittin: $p = 0.9968$; eYFP/Naïve vs. ChR2/Naïve: $p = 0.0110$; eYFP/Naïve vs. ChR2/Melittin: $p < 0.0001$; eYFP/Melittin vs. ChR2/Naïve: $p = 0.0161$; eYFP/Melittin vs. ChR2/Melittin: $p < 0.0001$; ChR2/Naïve vs. ChR2/Melittin: $p < 0.0001$
				Kruskal-Wallis test with Dunn's multiple comparisons	$p < 0.0001$	eYFP/Naïve vs. eYFP/Melittin: $p > 0.9999$; eYFP/Naïve vs. ChR2/Naïve: $p = 0.0062$; eYFP/Naïve vs. ChR2/Melittin: $p < 0.0001$; eYFP/Melittin vs. ChR2/Naïve: $p = 0.0205$; eYFP/Melittin vs. ChR2/Melittin: $p < 0.0001$; ChR2/Naïve vs. ChR2/Melittin: $p < 0.0001$
Supplemental Figure 9E	n = 5 female mice in each group	passed		Two-way ANOVA with Tukey's multiple comparisons		eYFP/Naïve vs. eYFP/Melittin: $p = 0.7867$; eYFP/Naïve vs. ChR2/Naïve: $p = 0.0003$; eYFP/Naïve vs. ChR2/Melittin: $p < 0.0001$; eYFP/Melittin vs. ChR2/Naïve: $p = 0.0022$; eYFP/Melittin vs. ChR2/Melittin: $p < 0.0001$; ChR2/Naïve vs. ChR2/Melittin: $p < 0.0001$
Supplemental Figure 10B	n = 7 male mice in each group	failed		Mann Whitney *U* test	$p = 0.1037$	
Supplemental Figure 10C	n = 7 male mice in each group	passed	passed	Unpaired *t* test	$p = 0.0035$	
Supplemental Figure 10D	n = 7 male mice in each group	passed	passed	Unpaired *t* test	$p = 0.0101$	

Supplemental Figure 11G	n = 10 and 8 male mice in each group	passed	passed	Unpaired *t* test	$p = 0.0215$	
Supplemental Figure 11H	n = 10 and 8 male mice in each group	passed	failed	Unpaired *t* test with Welch's correction	$p = 0.0014$	
Supplemental Figure 11I	n = 10 and 8 male mice in each group	passed	passed	Unpaired *t* test	$p = 0.0020$	
Supplemental Figure 12A	n = 80 to 93 trials from 5 male mice in each group	failed		Kruskal-Wallis test with Dunn's multiple comparisons	$p < 0.0001$	PVN^{AVP} soma vs. PVN^{AVP}-CeM terminal: $p > 0.9999$; PVN^{AVP} soma vs. CeM^{V1aR} soma: $p < 0.0001$; PVN^{AVP}-CeM terminal vs. CeM^{V1aR} soma: $p < 0.0001$
Supplemental Figure 12B	n = 80 to 93 trials from 5 male mice in each group	failed		Kruskal-Wallis test with Dunn's multiple comparisons	$p < 0.0001$	PVN^{AVP} soma vs. PVN^{AVP}-CeM terminal: $p > 0.9999$; PVN^{AVP} soma vs CeM^{V1aR} soma: $p = 0.0001$; PVN^{AVP}-CeM terminal vs. CeM^{V1aR} soma: $p = 0.0050$
Supplemental Figure 12C	n = 5 male mice in each group	passed	passed	One-way ANOVA with Tukey test multiple comparisons	$p = 0.0041$	PVN^{AVP} soma vs. PVN^{AVP}-CeM terminal: $p = 0.7441$; PVN^{AVP} soma vs CeM^{V1aR} soma: $p = 0.0047$; PVN^{AVP}-CeM terminal vs. CeM^{V1aR} soma: $p = 0.0177$
