# Supplementary material for: Hypothalamic-amygdala gating of caregiving-like prosocial behavior in mice
Source: J Clin Invest. 2026 Oct 1;136(19):e204317. doi: 10.1172/JCI204317 (PMC13626834; doi:10.1172/JCI204317)
Supplement: Supplementary file 2 [file jci-136-204317-s010.pdf]

Figure 2A-Genotyping
V1aR null mutant mouse line

Figure 3B-Genotyping
V1aR^{iCre} mouse line

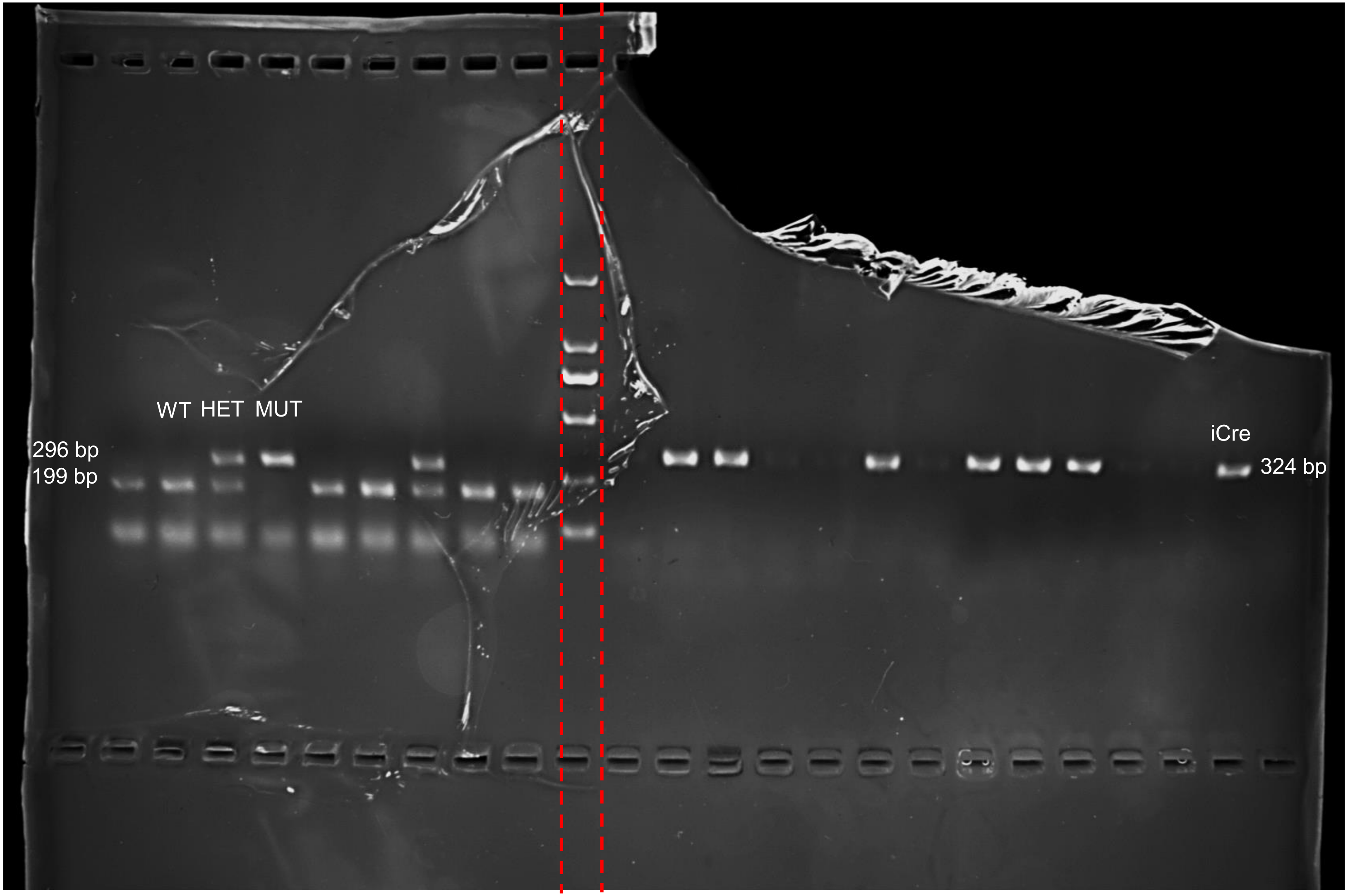

Marker band
